# Validity and reliability of inertial measurement units on gait, static balance and functional mobility performance among community-dwelling older adults: a systematic review and meta-analysis

**DOI:** 10.1530/EOR-2024-0088

**Published:** 2025-04-01

**Authors:** Lulu Yin, Xin Xu, Ruiyan Wang, Feifei Li, Yushan Wang, Lin Wang

**Affiliations:** ^1^ Key Laboratory of Exercise and Health Sciences (Shanghai University of Sport), Ministry of Education, Shanghai, China; ^2^ Shanghai Normal University Tianhua College, Shanghai, China; ^3^ School of Rehabilitation Science, Shanghai University of Traditional Chinese Medicine, Shanghai, China; ^4^ Guangdong OPPO Mobile Telecommunications Co. Ltd, Shenzhen, Guangdong, China; ^5^ Sports Medicine and Rehabilitation Center, Shanghai University of Sport, Shanghai, China; ^6^ Shanghai Shangti Orthopaedic Hospital, Shanghai, China

**Keywords:** validity, reliability, inertial measurement units, postural control, older adults

## Abstract

**Purpose:**

- This systematic review and meta-analysis investigated validity and test-retest reliability of inertial measurement units (IMUs) in gait metrics, static balance and functional mobility performance in community-dwelling older adults.

**Methods:**

- Spatiotemporal/biomechanical outcomes were meta-analyzed using intraclass correlation coefficients (ICCs) or Pearson correlation coefficients (r) for validity and reliability, respectively.

**Results:**

- In our systematic review of 56 articles and meta-analysis of 38 articles, the included studies varied in quality from low-to-moderate. During validity analysis, IMU-derived metrics, including walking speed, cadence, step/stride time, step time variability, step/stride length and duration of sit-to-stand (STS) test/timed up, and go test (TUGT) exhibited excellent (ICCs) or good-to-excellent (r values) agreement with gold standards. In terms of reliability, excellent test-retest consistency was found for walking speed, cadence, step/stride time, stance/swing time, step/stride length during gait, individual STS duration, TUGT duration and walking speed during the 6-min walk test.

**Conclusions:**

- Due to consistently high levels of validity and reliability, the present study supported the use of IMUs for measuring gait spatiotemporal outcomes. However, caution was advised when applying spatiotemporal variability and symmetry metrics. In addition, characterized by moderate-to-good validity and reliability, current review provides evidence of a neutral nature regarding the utilization of IMUs for static balance and functional mobility performance.

## Introduction

Population aging is a growing global concern, with the number of individuals aged 65 and older projected to rise from 761 million in 2021 to 1.6 billion by 2050 (1). This demographic shift brings increased health challenges, particularly falls, which contribute to injuries, fractures, functional impairments and psychological consequences such as the fear of falling (2). Falls result from a complex interaction of biological, behavioral, environmental and socioeconomic factors (3). Postural control, involving the intricate coordination among these – encompassing the coordination of sensory, neural and motor systems – emerges as a key biological risk factor. Understanding the mechanisms underlying postural control and employing accurate assessment methods is essential for addressing fall risk and enhancing the quality of life in older adults (4, 5, 6).

Postural control is commonly assessed using observational methods, questionnaire-based assessments and instrumented measurements (7, 8, 9). While observational and questionnaire approaches are convenient for clinical settings, their reliability and validity can be affected by subjective bias (10, 11). In contrast, instrumented measurements offer greater precision but are limited by high costs, complexity and limited portability (12). Given these limitations, there is a need for innovative methods to accurately assess postural control in older adults. Wearable technologies have emerged as promising alternatives, providing both accuracy and portability for daily gait analysis (13, 14).

Inertial measurement units (IMUs), consisting of accelerometers, gyroscopes and magnetometers, are commonly used to measure postural control by capturing real-time motion data from key body segments. Despite their widespread use, the reliability and validity of IMUs in postural control measurement remain uncertain due to technical challenges such as cumulative errors, positional drift and the need for recalibration. Kobsar *et al.* (15) conducted investigation on the validity and reliability of wearable inertial sensors in the context of healthy adult walking. Zeng *et al.* (16) investigated the validity and reliability of IMUs in lower-extremity kinematics during running. In the elderly population, some systematic reviews have focused on the validation and reliability of IMUs for people with diseases, such as stroke (17), multiple sclerosis (18) and Parkinson diseases (19); few studies have summarized the application situations of IMUs for elderly people in communities. Nonetheless, a notable gap persists in the form of the dearth of systematic reviews and meta-analyses aimed at quantifying the reliability and validity of IMUs concerning gait, postural control and functional mobility in community-dwelling elderly populations. Therefore, evidence supporting the recommendation for the use of IMUs in conducting postural control tests for community-dwelling elderly individuals is currently lacking.

Therefore, the purpose of this review was to undertake a systematic review and subsequent meta-analysis to ascertain the levels of test-retest reliability and concurrent validity provided by IMUs in the assessment of gait, postural control and functional mobility performance among community-dwelling older adults. By integrating qualitative and quantitative analyses, this study would provide sufficient evidence-based evidence and valuable recommendations for real-world use of IMUs during elderly individuals.

## Materials and methods

The protocol was registered in the International Prospective Register of Systematic Reviews (PROSPERO) (registration number: CRD42023459956) and followed the preferred reporting items for systematic reviews and meta-analysis (PRISMA) guidelines (https://www.prisma-statement.org/) (20).

### Search strategy

PubMed, Embase, Scopus, Cochrane library, Ovid MEDLINE and web of science electronic databases were searched from inception until Nov 30, 2024. The search terms and strategies included: (Wearable Electronic Devices (Mesh) OR Micro-Electrical-Mechanical Systems (Mesh) OR Smartphone (Mesh) OR wearable sensor* OR inertial sensor* OR inertial motion capture OR IMU* OR IMU OR IMUs OR acceleromet* OR gyroscop* OR magnetomet* ) AND (spatio-temporal analysis (Mesh) OR postural balance (Mesh) OR gait* OR cadence OR step frequency OR stride frequency OR step time OR stride time OR cycle time OR contact time OR swing time OR step length OR stride length OR sit-to-stand (STS) OR chair-stand OR chair-rise OR timed up and go OR timed-up-and-go OR timed-up and go OR timed up & go OR timed-up-&-go OR Timed up-and-go OR timed up-&-go OR get up and go OR TUG OR TUGT OR six min walk OR six min walk OR six min walk OR six min walk OR 6 min walk OR 6 min walk OR 6 min walk OR 6 min walk OR 6 MW OR 6 MWT (6 min walk test) OR 6-MW OR 6-MWT) AND (aging OR elder* OR older adult*) AND (Reproducibility of Results (Mesh) OR data accuracy (Mesh) OR validity OR reliability OR feasibility OR repeatability OR consistency). Minor adjustments were made to different databases. The full search strategy for each database is described in Supplementary Information 1 (see section on Supplementary materials given at the end of the article).

### Inclusion and exclusion criteria

Articles that met the following criteria were included in this systematic review: i) evaluated the validity or reliability of IMUs; ii) measured specific gait spatiotemporal parameters, static postural balance and functional mobility test outcomes including STS test, timed up and go test (TUGT) and 6 MWT; iii) compared the measurements captured by IMUs with those obtained using reference systems; iv) collected data among community-dwelling older adults; and v) were published in English. Studies that only measured event detection or energy expenditure were excluded from this review. Additional details on the inclusion and exclusion criteria and definitions of spatiotemporal parameters are provided in Supplementary Information 2.

### Study selection

After duplicate articles were removed, two independent reviewers screened titles and abstracts according to the eligibility criteria. Full-text screening of potentially eligible articles was performed by one author and rechecked by a second author. All reference lists and bibliographies of the retrieved studies were reviewed if relevant studies were missing in the electronic search. Disagreements were resolved by a third reviewer. According to the standards for systematic review, qualitative synthesis and quantitative synthesis were performed (21).

### Qualitative analysis

#### Assessment of risk of bias

Risk of bias was assessed using a modified version of the Critical Appraisal of Study Design for Psychometric Articles (22). This checklist contains 12 items that assess the methodological quality of five domains: the study question, study design, measurements, analyses and recommendations. Each item comprises three descriptors. The maximum score was 24. Initially, two assessors independently reviewed two articles. For articles with any disagreements, they discussed the scoring and interpretation together to reach a consensus before proceeding with the evaluation of the remaining articles. The assessors were blinded to any identifiable information related to the studies to avoid bias in the quality assessment. Furthermore, agreement between the two assessors was calculated using the Cohen’s kappa coefficient, with a 95% confidence interval (95% CI). Cohen’s kappa coefficients of <0.40, 0.40–0.75 or >0.75 were regard as poor, fair-to-good or excellent, respectively, (23).

#### Assessment of quality

To grade the quality of the study, a previously described classification scheme was applied, namely, high quality (HQ) with 85–100% scores, moderate quality (MQ) with 70–85% scores, low quality (LQ) with 50–70% scores and very low quality (VLQ) with <50% scores. Quality assessment scoring was used to determine the strength of the recommendations (24).

#### Assessment of heterogeneity

Heterogeneity was examined using Tau^2^, Chi^2^ and *I*^2^ statistics, where, Tau^2^ = 0 suggested no heterogeneity; *I*^2^ values of <25%, 26%–50% and >75% indicated low, moderate and high heterogeneity, respectively; and a significant Chi^2^ indicates heterogeneity.

#### Data extraction

Data extraction was completed by two authors using a predefined form. The data consisted of: i) study identification information; ii) participant characteristics: sample size, sex, age, height and weight; iii) IMUs’ specifications: name, manufacturer, composition, number used, placement and sample frequency; iv) reference systems used; v) study design: walking speed or distance and different conditions during postural balance; vi) specific parameters; and vii) reported statistical outcomes.

### Quantitative analysis

#### Statistical outcomes for reliability and validity

For both reliability and validity analyses, statistical outcomes included Pearson correlation coefficient (r), coefficient of determination (*r*^2^), coefficient of multiple correlation, intraclass correlation coefficient (ICC) with 95% CI, root mean square error (RMSE), bias (mean difference), limits of agreement (LoA), coefficient of variation (CV) and standard error of the mean (SEM) (25). Outcomes shown graphically without specific values were excluded. Data pooling was pre-defined to focus on ICCs, r and sample size for validity and reliability.

#### Data pooling and dichotomization

Validity and reliability were dichotomized for data pooling, with further division based on specific outcome parameters (e.g. walking speed and step time). Data were not pooled by sensor type (e.g., accelerometer vs gyroscope) or algorithm. A single study may contribute to multiple independent pools based on validity, statistical outcomes and measured parameters. ICCs were categorized as poor (<0.500), moderate (0.500–0.749), good (0.750–0.899) or excellent (≥0.900) (26), and r as no correlation (<0.250), fair (0.250–0.500), moderate-to-good (0.500–0.750) or good-to-excellent (≥0.750) (27).

#### Subgroup and sensitivity analysis

Subgroup analyses were performed when at least two studies were available, exploring heterogeneity by movement speed (slow, preferred or fast), visual conditions during static balance (eyes open/closed) and IMU placement (back, shank or ankle/foot). Sensitivity analyses were conducted by sequentially removing one study at a time and recalculating summary correlation coefficients, weighted by sample size. Fisher’s z-transformation was applied to stabilize variance due to the non-normality of ICCs and r (28):

$${\text{Fisher}}^{\prime }s\;Z_{\text{ICC}}=0.5\times \ln\frac{1+\text{ICC}}{1-\text{ICC}}$$

$${\text{Fisher}}^{\prime }s\;Z_{r}=0.5\times \ln\frac{1+r}{1-r}$$

$${\text{SE}}_{\text{ICC}}=\frac{1}{\sqrt{n-3/2}}$$

$${\text{SE}}_{r}=\sqrt{\frac{1}{n-3}}$$

$$\text{Summary}\;\text{ICC}/r=\frac{e^{2z}-1}{e^{2z}+1}$$

where, ‘*n*’ represents sample sizes, ‘SE’ represents the standard error and ‘*Z*’ signifies the summary Fisher’s *Z* value (29). The data were then transformed back to ICCs or r for reporting.

#### Random effects model

The review manager (RevMan 5.4) was used for the meta-analysis (https://training.cochrane.org/revman). A random effects model was used for the meta-analysis due to the heterogeneity of the experimental conditions and population (30). Statistical significance was *P* < 0.05.

#### Meta-analysis interpretation and level of evidence

The results of the meta-analysis were interpreted by using the agreement metrics outlined above. Statistical results that were not included in the quantitative analysis were included in the qualitative analysis to support the interpretation. An adapted rating system from the Cochrane collaboration back-review group was used to determine the level of evidence for each parameter (Table 1) (24).

**Table 1:** Definitions of levels of evidence.

Level of evidence	Criteria
Strong evidence	Consistent results in HQ studies (*n* ≥ 2)
Moderate evidence	Consistent results among multiple MQ studies (*n* ≥ 2)
Limited evidence	Consistent results among multiple LQ studies (*n* ≥ 2)
Conflicting evidence	Inconsistent results among multiple studies
Very limited evidence	Only one LQ or MQ study or multiple VLQ studies

HQ, high quality; MQ, moderate quality; LQ, low quality; VLQ, very low quality.

## Results

### Search results

**Figure 1:**
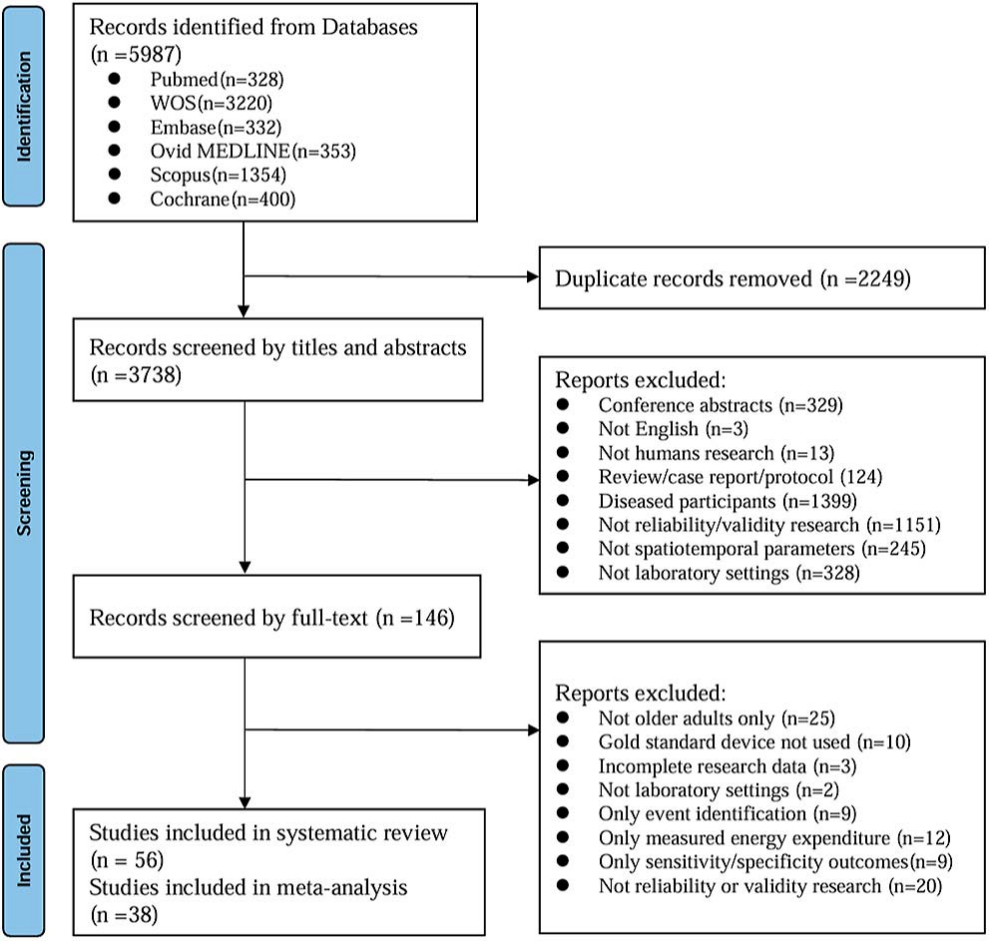
Flowchart of the screen outline of this systematic review and meta-analysis.

The search strategy outline was recommended by the PRISMA (Fig. 1) A total of 5987 articles were identified. Following the removal of duplicate literature, screening of titles and abstracts and full-text readings, 56 articles were deemed eligible for the scope of this systematic review. 38 articles were included in the meta-analysis due to the availability of sufficient data.

### Methodological quality

As shown in Table 2, 36 articles were rated as MQ (31, 32, 34, 35, 36, 37, 38, 40, 41, 42, 43, 45, 47, 49, 50, 55, 56, 57, 59, 60, 62, 64, 65, 66, 68, 72, 73, 74, 75, 76, 77, 78, 80, 81, 83, 85) and 20 articles as LQ (33, 39, 44, 46, 48, 51, 52, 53, 54, 58, 61, 63, 67, 69, 70, 71, 79, 82, 84, 86). Notably, no articles received HQ or VLQ ratings. The agreement between the two raters reached a good/excellent level (Cohen’s kappa = 0.914, 95% CI = 0.90, 0.93). The items generally rated higher score were ‘Q1 background and research question’, ‘Q4 scope/design’ and ‘Q12 conclusions/recommendations’. In contrast, most of the studies performed a sample size calculation process (Q5), and a few articles described participant retention (Q6) and estimates of variance (Q11).

**Table 2:** Quality assessment scoring of 56 included studies.

Reference	Q1	Q2	Q3	Q4	Q5	Q6	Q7	Q8	Q9	Q10	Q11	Q12	Total	%	Quality
Adamowicz *et al.* (31)	2	2	0	2	1	NA	2	2	2	2	1	2	18/24	75%	MQ
Alqahtani *et al.* (32)	2	2	0	2	1	2	1	2	2	2	1	2	19/24	79.16%	MQ*
Álvarez *et al.* (33)	2	2	1	0	1	NA	1	2	1	1	1	2	14/24	58.33%	LQ*
Bäcklund *et al.* (34)	2	2	1	2	1	2	1	2	2	2	1	2	20/24	83.33%	MQ
Bautmans *et al.* (35)	2	2	1	2	1	NA	1	2	1	2	2	2	18/24	75%	MQ*
Bochicchio *et al.* (36)	2	2	2	2	1	NA	2	2	1	2	2	2	20/24	83.33%	MQ*
Burton *et al.* (37)	2	2	1	2	1	NA	1	2	2	2	1	2	18/24	75%	MQ
Byun *et al.* (38)	2	2	2	2	1	NA	1	2	2	2	1	2	19/24	79.16%	MQ*
Byun *et al.* (39)	2	2	1	2	1	NA	1	2	2	1	2	0	16/24	66.67%	LQ*
Cerrito *et al.* (40)	2	2	1	2	1	NA	1	2	1	2	2	2	18/24	75%	MQ*
Chan *et al.* (41)	2	2	2	2	1	NA	1	2	1	2	1	1	17/24	70.83%	MQ*
Cole *et al.* (42)	2	2	1	2	0	NA	1	2	1	2	2	2	17/24	70.83%	MQ
Contreras *et al.* (43)	2	2	1	2	2	NA	2	2	2	2	1	2	20/24	83.33%	MQ*
De Groote *et al.* (44)	2	1	0	2	1	NA	1	2	2	1	2	2	16/24	66.76%	LQ*
Digo *et al.* (45)	2	2	1	2	0	NA	2	2	1	2	1	2	17/24	70.83%	MQ*
Donath *et al.* (46)	1	1	1	2	1	NA	1	2	1	2	1	2	15/24	62.50%	LQ*
Ensink *et al.* (47)	2	2	2	2	1	NA	1	2	2	2	2	2	20/24	83.33%	MQ
Ferrari *et al.* (48)	2	2	2	2	0	NA	1	2	2	1	1	2	17/24	70.83%	LQ
Foster *et al.* (49)	2	2	1	2	1	NA	1	2	1	2	2	2	18/24	75%	MQ
Fudickar *et al.* (50)	2	2	1	2	2	NA	2	2	2	2	1	2	20/24	83.33%	MQ*
Greene *et al.* (51)	2	1	1	2	1	NA	2	2	2	1	0	2	16/24	66.67%	LQ*
Grimpampi *et al.* (52)	2	2	1	2	2	NA	2	2	2	1	0	1	17/24	70.83%	LQ
Hamacher *et al.* (53)	1	1	0	2	0	NA	2	2	1	2	0	1	13/24	54.17%	LQ*
Hartmann *et al.* (54)	2	1	1	2	1	NA	1	2	1	2	1	2	16/24	66.67%	LQ*
Hartmann *et al.* (55)	2	1	1	2	1	NA	2	2	2	2	2	2	19/24	79.16%	MQ*
Hellmers *et al.* (56)	2	2	1	2	1	NA	1	2	2	2	1	2	18/24	75%	MQ*
Kobsar *et al.* (57)	2	2	2	2	1	NA	1	2	1	2	1	2	18/24	75%	MQ
Koose *et al.* (58)	2	2	0	1	1	NA	1	1	2	1	2	2	15/24	62.50%	LQ*
Kuntapun *et al.* (59)	2	2	2	2	1	NA	1	2	2	1	0	2	17/24	70.83%	MQ*
Maganja *et al.* (60)	2	2	1	2	2	NA	1	2	2	2	2	2	20/24	83.33%	MQ
Maggio *et al.* (61)	2	2	1	2	1	NA	0	2	1	1	0	2	14/24	58.33%	LQ*
Magistro *et al.* (62)	2	2	2	1	1	2	1	2	1	1	1	2	18/24	75%	MQ*
Mancini *et al.* (63)	2	1	2	2	0	NA	1	2	2	1	1	2	16/24	66.67%	LQ*
Marques *et al.* (64)	2	2	2	2	1	NA	1	2	1	2	2	2	19/24	79.16%	MQ*
Matikainen-Tervola *et al.* (65)	2	2	2	2	0	1	1	2	2	2	2	2	20/24	83.33%	MQ*
Micó-Amigo *et al.* (66)	2	2	2	2	1	NA	1	2	2	1	1	2	18/24	75%	MQ*
Motti Ader *et al.* (67)	2	2	1	2	1	NA	2	2	2	1	0	2	17/24	70.83%	LQ*
Orange *et al.* (68)	2	2	0	2	1	NA	1	2	2	2	2	2	18/24	75%	MQ*
Ozinga *et al.* (69)	2	2	0	2	0	NA	1	2	2	1	2	0	14/24	58.33%	LQ
Pedrero-Sánchez *et al.* (70)	2	2	0	1	1	NA	1	2	2	1	0	2	14/24	58.33%	LQ
Peller *et al.* (71)	2	2	0	1	1	NA	0	1	2	1	0	2	12/24	50%	LQ
Phillips *et al.* (72)	2	1	1	2	1	NA	1	2	1	2	2	2	17/24	70.83%	MQ
Pooranawatthanakul *et al.* (73)	2	2	1	2	2	NA	2	2	2	2	1	2	20/24	83.33%	MQ*
Rantalainen *et al.* (74)	2	2	1	2	1	NA	1	2	1	2	2	2	18/24	75%	MQ*
Rantalainen *et al.* (75)	2	2	1	2	1	NA	1	2	1	2	2	2	18/24	75%	MQ*
Regterschot *et al.* (76)	2	2	2	2	2	NA	1	2	2	2	1	2	20/24	83.33%	MQ*
Regterschot *et al.* (77)	2	2	2	2	1	NA	1	2	2	2	2	2	20/24	83.33%	MQ
Rogan *et al.* (78)	2	2	1	2	1	NA	1	2	2	2	1	2	18/24	75%	MQ*
Rüdiger *et al.* (79)	2	2	1	2	1	NA	1	2	2	1	0	2	16/24	66.67%	LQ
Rudisch *et al.* (80)	2	2	1	2	1	NA	1	2	2	2	2	2	19/24	79.16%	MQ*
Saunders *et al.* (81)	2	2	2	2	1	NA	1	2	1	2	1	2	18/24	75%	MQ*
Smith *et al.* (82)	2	2	1	2	0	NA	1	2	1	2	1	2	16/24	66.67%	LQ*
Song *et al.* (83)	2	2	2	2	1	NA	1	2	1	2	1	2	18/24	75%	MQ
Walgaard *et al.* (84)	2	1	1	2	1	NA	1	2	2	1	1	2	16/24	66.67%	LQ
Werner *et al.* (85)	2	2	1	2	2	2	0	2	2	2	2	2	21/24	87.5%	MQ*
Zhang *et al.* (86)	2	2	1	2	1	NA	1	2	1	2	0	2	16/24	66.67%	LQ*

* Studies included in the meta-analysis.

NA, not mentioned; LQ, low quality; MQ, medium quality.

### Characteristics of included studies

The detailed characteristics of the 56 articles are presented in Supplementary Information 3. A total of 2658 community-dwelling adults were included, comprising 1013 males and 1562 females (gender composition was not reported in Mancini *et al.* (63), Pedrero-Sánchez *et al.* (70) and Rogan *et al.* (78)). The sample size of most studies ranged from 12 to 197 participants. Some studies focused specifically on subgroups, such as healthy older adults or elderly individuals with chronic diseases. To minimize confounding factors of diseases, this study only included data from healthy older adults. The studies spanned from 2009 to 2024, reflecting the recent applications of IMU technology in gait and balance assessment. The study designs primarily consisted of cross-sectional studies and cohort studies.

The most common applications of IMU systems are as follows: smartphones (*n* = 6) (41, 44, 59, 68, 73, 87), Dynaport (*n* = 5) (35, 54, 55, 66, 84), followed by Xsens (*n* = 4) (45, 47, 53, 63), Shimmer (*n* = 3) (51, 67, 82), Fitbit (*n* = 3) (37, 60, 72), ADXL (*n* = 3) (32, 57, 62) and Apple devices (*n* = 3) (58, 69, 83).

Of these studies, 21 exclusively utilized the accelerometer (32, 35, 37, 49, 54, 55, 57, 58, 59, 60, 61, 62, 63, 64, 72, 74, 75, 79, 81, 83, 86), while 12 explicitly mentioned employing both accelerometer and gyroscope components in their data analysis (38, 39, 44, 51, 66, 67, 69, 70, 78, 82, 84). Furthermore, one study utilized a gyroscope and magnetometer (36) and another study employed a triaxial accelerometer and a rotation vector sensor (40). In contrast, the remaining 19 studies either did not provide explicit information on the components used (31, 33, 34, 41, 42, 47, 52, 53, 68, 71, 73, 80, 85, 87, 88) or implied they used all accelerometer, gyroscope and magnetometer components for the data analysis (45, 46, 50, 56, 76, 77, 89).

Across these studies, the most common sampling frequency was sequentially 100 Hz (*n* = 12) (35, 42, 47, 50, 54, 55, 56, 57, 66, 69, 70, 84, 87), 50 Hz (*n* = 9) (31, 45, 59, 62, 63, 73, 76, 77, 86) and 500 Hz (*n* = 5) (36, 44, 46, 78, 88). Most of the studies employed either one (*n* = 34) (32, 35, 36, 38, 39, 41, 42, 44, 50, 52, 53, 54, 55, 56, 57, 58, 61, 63, 64, 66, 68, 69, 70, 71, 73, 74, 76, 79, 81, 83, 84, 85, 86, 88) or two (*n* = 15) IMU sensors (34, 37, 40, 46, 51, 59, 62, 66, 67, 75, 77, 78, 80, 82, 87).

The most commonly utilized inertial wearable locations were the back (*n* = 30) (31, 32, 35, 38, 39, 40, 42, 44, 45, 47, 50, 52, 54, 55, 56, 57, 58, 59, 61, 63, 66, 69, 70, 72, 75, 81, 84, 88, 89), predominantly located at the level of the 3rd–5th lumbar vertebra, varying locations of chest (31, 40, 41, 47, 51, 68, 73, 77, 86), wrist (31, 37, 49, 60, 62, 64, 72), arm (62, 79), thigh (36, 49, 51, 59, 60, 76, 77, 83, 85, 87), shank (34, 45, 67, 82, 89), ankle (45, 72, 74, 75, 78), foot (33, 47, 53, 66, 80) and shoes (46).

### Qualitative and quantitative synthesis

#### Validity

As shown in Supplementary Table 1 presented in Supplementary Information 5, during gait-related articles, a total of 18 spatiotemporal outcomes and one biomechanical outcome were extracted from 21 articles (33, 34, 38, 39, 42, 45, 49, 54, 59, 60, 61, 62, 66, 72, 74, 75, 78, 79, 80, 87, 89), which assessed the validity of IMUs. Ten spatiotemporal outcomes had sufficient statistical qualifications for data pooling (Table 3). Walking speed, cadence, step/stride time, step time variability, stance time and step/stride length during gait exhibited excellent (ICCs) or good-to-excellent (r values) agreement with gold standards. Notably, subgroup analysis results showed significant effect based on walking speed on validity of walking speed (*P* < 0.001), step time (*P* < 0.001) and step length (*P* < 0.001). However, moderate-to-good agreement for stride time SD (standard deviation) and poor agreement for swing time were observed during walking gait. Forest plots of quantitative analysis are presented in Supplementary Information 4 Figs 1, 2, 3, 4, 5, 6, 7, 8.

**Table 3:** Quantitative pooling results for validity of outcomes derived from IMUs.

Outcomes	Articles			Results		*I* ^2^	Subgroup analysis, *P*^†^		Sensitivity analysis
	QTY	*n*	Reference	Summary	ICC/r (95%CI)		Locations	Speed	
Gait									
Walking speed	MQ	5	(37, 73, 77, 79, 88)	Excellent	0.954 (0.903,0.978)	98%	0.59	**<0.001**	Stable
	LQ	2	(38, 53)						
	LQ	1	(60)	Good-to-excellent	0.869 (0.744, 0.937)*	88%	NA	NA	Stable
	MQ	3	(37, 58, 86)						
Cadence	MQ	3	(37, 77, 79)	Excellent	0.992 (0.947, 0.999)	98%	0.57	0.11	Stable
	LQ	1	(53)						
	LQ	1	(37)	Good-to-excellent	0.993 (0.848,0.999)*	98%	NA	NA	Stable
	MQ	2	(58, 86)						
Step time	MQ	4	(37, 65, 77, 88)	Excellent	0.960 (0.854,0.990)	97%	0.75	**<0.001**	NA
	LQ	1	(53)						
	MQ	3	(37, 44, 58)	Good-to-excellent	0.965 (0.903, 0.987)*	93%	0.19	0.90	Stable
Step time variability	MQ	2	(37, 53)	Excellent	0.998 (0.995, 0.999)	74%	NA	NA	NA
Stride time	MQ	4	(73, 74, 79, 88)	Excellent	0.997 (0.990, 0.999)	89%	NA	NA	Stable
Stride time SD	MQ	2	(73, 74)	Moderate	0.565 (−0.327, 0.925)	94%	NA	NA	NA
Stance time	MQ	2	(73, 88)	Excellent	0.973 (−0.808, 0.999)	99%	NA	NA	NA
Swing time	MQ	2	(73, 88)	Poor	0.310 (0.040, 0.537)	100%	NA	NA	NA
Step length	MQ	4	(37, 77, 79, 88)	Excellent	0.960(0.854, 0.990)	97%	0.39	**<0.001**	Stable
	LQ	1	(53)						
	MQ	3	(37, 58, 86)	Good-to-excellent	0.834 (0.774,0.879)*	0%	NA	NA	Unstable
Stride length	MQ	1	(73)	Excellent	0.992 (0.964, 0.998)	93%	NA	NA	NA
	LQ	1	(53)						
STS test									
Individual STS duration	MQ	2	(35, 39)	Good-to-excellent	0.989 (0.982, 0.993)*	23%	NA	NA	NA
STS mean power	MQ	2	(35, 67)	Moderate-to-good	0.726 (0.523, 0.854)*	55%	NA	NA	NA
STS mean velocity	MQ	2	(35, 67)	Moderate-to-good	0.749 (0.641, 0.831)*	0%	NA	NA	NA
Timed up-and-go test									
TUGT duration	MQ	2	(49, 55)	Good-to-excellent	0.959 (0.793, 0.992)*	99%	NA	NA	NA

IMUs, inertial measurement units; SD, standard deviation; STS, sit to stand test; TUGT, timed up and go test; MQ, moderate quality; LQ, low quality; ICCs, intraclass correlation coefficients; r, Pearson correlation coefficients; NA, no subgroup/sensitivity analysis was carried out due to insufficient data; QTY, quality.

* Indicates r (95% CI) values;

^†^ Statistically significant values are in bold.

In static balance-related articles, as shown in Supplementary Table 2  presented as Supplementary Information 5, seven biomechanical outcomes were extracted from three articles (44, 69, 73), which assessed the IMU validity. The validity of the measurements varied, with some showing strong agreement (*r* > 0.9), such as ellipsoid volume and RMS (root mean square) COM (center ofmass), while others, such as mean acceleration and RMS acceleration, exhibited moderate validity (r between 0.7 and 0.9). No outcomes could be pooled because of the lack of consistency among the outcomes.

During the STS test, as shown in Supplementary Table 3 during Supplementary Information 5, nine biomechanical outcomes were extracted from nine articles (31, 36, 40, 41, 50, 64, 68, 77, 83) that assessed IMU validity. The validity of measurements varied from moderate-to-excellent. Duration of STS test exhibited excellent (ICCs) or good-to-excellent (r values) agreement with gold standards (Table 3). However, moderate-to-good agreement was observed for mean power and mean velocity of STS (Table 3). Forest plots of quantitative analysis are presented in Supplementary Information 4 Fig. 9.

During TUGT, as shown in Supplementary Table 4 presented in Supplementary Information 5, six biomechanical outcomes were extracted from five articles (41, 50, 56, 62, 84), which assessed the validity. Only TUGT duration exhibited excellent (ICCs) or good-to-excellent (r values) agreement with gold standards (Table 3). Forest plots of quantitative analysis are presented in Supplementary Information 4 Fig. 9.

During the 6 MWT, as shown in Supplementary Table 5 presented in Supplementary Information 5, five spatiotemporal outcomes were extracted from three articles (37, 46, 57) that assessed validity. No outcomes could be pooled because of the lack of consistency among the outcomes.

Meta-analysis was not possible for other outcomes due to the limited quantity or consistency in data reporting; many studies only reported RMSE or even a simple bias. Articles that were not eligible for data pooling are qualitatively summarized in Supplementary Information 5.

#### Reliability

As shown in Supplementary Table 6 presented in Supplementary Information 5, during gait-related articles, a total of 28 spatiotemporal outcomes were extracted from 12 articles (34, 35, 38, 55, 56, 59, 60, 62, 67, 74, 75, 87) that assessed IMU reliability during different walking conditions. Fourteen spatiotemporal outcomes showed sufficient statistical qualifications for data pooling (Table 4) Excellent test-retest consistency was found for walking speed, cadence, step/stride time, stance/swing time and step/stride length during gait; good consistency was observed for swing time variability; and moderate consistency was noted for step/stride time variability, step time asymmetry and stride time SD during gait. Notably, subgroup analysis results showed significant effect based on walking speed on validity of walking speed (*P* < 0.001). Forest plots of quantitative analysis are presented in Supplementary Information 4 Figs 10, 11, 12, 13, 14, 15, 16.

**Table 4:** Quantitative pooling results for reliability of outcomes derived from IMUs.

Outcomes	Articles			Results		*I* ^2^	Subgroup analysis, *P^†^*	Sensitivity analysis
	QTY	*n*	Reference	Summary	ICC (95%CI)			
Gait								
Walking speed	MQ	6	(34, 37, 54, 58, 73, 86)	Excellent	0.962 (0.935, 0.977)	90%	LS: **0.001**	Stable
Cadence	MQ	4	(37, 54, 58, 86)	Excellent	0.953 (0.917, 0.973)	62%	NA	Unstable
Step time	MQ	3	(37, 54, 58)	Excellent	0.941 (0.872, 0.973)	90%	NA	Stable
	LQ	1	(66)					
Step time variability	MQ	2	(37, 54)	Moderate	0.653 (0.578, 0716)	78%	NA	Stable
	LQ	1	(66)					
Step time asymmetry	MQ	2	(34, 37)	Moderate	0.647 (0.397, 0.808)	90%	NA	Stable
	LQ	1	(66)					
Stride time	MQ	2	(73, 74)	Excellent	0.971 (0.915, 0.990)	91%	NA	Unstable
	LQ	1	(66)					
Stride time SD	MQ	2	(73, 74)	Moderate	0.515 (0.327, 0.664)	94%	NA	NA
Stride time variability	MQ	1	(74)	Moderate	0.716 (0.438, 0869)	89%	NA	Stable
	LQ	2	(52, 66)					
Stance time	MQ	1	(73)	Excellent	0.972 (0.917, 0991)	92%	NA	Stable
	LQ	2	(52, 66)					
Stance time variability	LQ	2	(52, 66)	Good	0.757 (0.501, 0892)	84%	NA	NA
Swing time	MQ	1	(73)	Excellent	0.978 (0.937, 0992)	91%	NA	Stable
	LQ	2	(52, 66)					
Swing time variability	LQ	2	(52, 66)	Good	0.818 (0.558,0933)	89%	NA	NA
Step length	MQ	4	(37, 54, 58, 86)	Excellent	0.947 (0.881, 0.977)	89%	NA	Unstable
Stride length	MQ	2	(73, 86)	Excellent	0.976 (0.938, 0.991)	91%	NA	Stable
	LQ	2	(52, 66)					
Static balance test								
RMS acceleration during double leg stance	MQ	3	(31, 72, 80)	Good	0.774 (0.711, 0.824)	75%	VS: 0.68	Unstable
	LQ	3	(43, 57, 62)					
RMS acceleration during semi-tandem stance	MQ	1	(31)	Moderate	0.749 (0.611, 0.843)	79%	NA	Stable
	LQ	2	(43, 57)					
Sit to stand test								
Individual STS duration	MQ	2	(39, 75)	Good	0.770 (0.647, 0.856)	82%	MS: 0.44	Stable
	LQ	2	(50, 85)					
Total STS duration	MQ	1	(75)	Good	0.856 (0.814, 0.890)	37%	NA	Stable
	LQ	2	(50, 85)					
Stand-up duration	MQ	1	(63)	Good	0.778 (0.354, 0.938)	97%	NA	NA
	LQ	1	(50)					
STS maximal velocity	MQ	1	(75)	Good	0.869 (0.811, 0.909)	0%	MS: 0.37	NA
	LQ	1	(85)					
STS peak power	MQ	2	(35, 67)	Moderate	0.887 (0.762, 0.948)	79%	MS: 0.07	NA
STS maximal jerk	MQ	1	(75)	Moderate	0.716 (0.572, 0.814)	37%	MS: 0.05	NA
	LQ	1	(85)					
TUGT								
TUGT duration	MQ	1	(75)	Excellent	0.935 (0.793, 0.980)	88%	MS: 0.98	Stable
	LQ	2	(81, 85)					
6 MWT								
Walking speed	MQ	1	(84)	Excellent	0.989 (0.971, 0.996)	89%	MS: 0.26	Stable
	LQ	1	(45)					
Stride length	MQ	1	(84)	Good	0.894 (0.831, 0.935)	63%	MS: 0.82	Stable
	LQ	1	(45)					

IMUs, inertial measurement units; 6 MWT, 6 min walk test; RMS, root mean square; SD, standard deviation; STS, sit to stand test; TUGT, timed op and go test; MQ, moderate quality; LQ, low quality; ICCs, intraclass correlation coefficients; *r*, Pearson correlation coefficients; NA, no subgroup/sensitivity analysis was carried out due to insufficient data; LS, locations subgroup; VS, visual subgroup; MS, movement speed subgroup.

^†^ Statistically significant values (*P* < 0.05) are in bold.

In static balance-related articles, as shown in Supplementary Table 7 presented in Supplementary Information 5, a total of ten biomechanical outcomes were extracted from eight articles (32, 44, 58, 63, 70, 71, 73, 81) that assessed IMU reliability during different balance measurement conditions. These biomechanical outcomes provided sufficient statistical qualifications for data pooling (Table 4). Good consistency was observed for RMS acceleration during double-leg stance, while moderate consistency was noted for RMS acceleration during semi-tandem stance. Forest plots of quantitative analysis are presented in Supplementary Information 4 Figs 17 and 18.

During the STS test, as shown in Supplementary Table 8 presented in Supplementary Information 5, twelve biomechanical outcomes were extracted from five articles (40, 51, 64, 76, 86) that assessed IMU reliability. Six biomechanical outcomes showed sufficient statistical qualification for data pooling (Table 4). Excellent test-retest consistency was found for individual STS duration; good consistency was observed for total STS duration, stand-up duration and STS maximal velocity; and moderate consistency was noted for STS maximal jerk and STS peak power. Forest plots of quantitative analysis are presented in Supplementary Information 4 Figs 19 and 20.

During TUGT, as shown in Supplementary Table 9 ptesented in Supplementary Information 5, five biomechanical outcome was extracted from three articles (62, 76, 82, 86) that assessed reliability. The TUGT duration was used for data pooling (Table 4). Excellent test-retest consistency was found for TUGT duration. Forest plots of quantitative analysis are presented in Supplementary Information 4 Fig. 21.

During the 6 MWT, as shown in Supplementary Table 10 presented in Supplementary Information 5, five spatiotemporal outcomes and one biomechanical outcome were extracted from four articles (37, 46, 52, 85), which assessed reliability. Walking speed and stride length had sufficient statistical qualifications for data pooling (Table 4). Excellent test-retest consistency was found for walking speed during the 6 MWT, while good consistency was observed for stride length during the 6 MWT. Forest plots of quantitative analysis are presented in Supplementary Information 4 Figs 22 and 23.

## Discussion

This review aimed to determine the concurrent validity and test-retest reliability of gait analysis, static balance and functional mobility performance derived from IMUs in community-dwelling older adults. 56 articles, examining spatiotemporal parameters and biomechanical outcomes during gait, static balance test, STS test, TUGT and 6 MWT, were included in this review. Among them, 38 articles were included in the quantitative data synthesis.

### Qualitative analysis

#### Study quality and reporting limitations

Of the included studies, 20 were rated as LQ, 36 as MQ and none as HQ or VLQ. Only a few studies performed sample size calculations (Q5), described sensor calibration procedures (Q7) or provided variance estimates (Q11). The main factors contributing to LQ were unjustified sample sizes and the lack of multilevel statistical parameters. A few studies (47, 50, 52, 60, 73, 76, 85) provided detailed sample size calculations. Notably, the calibration process, critical for IMU data accuracy, was rarely reported across various tests (gait (45, 53, 55, 67), static balance (51), STS (31, 36, 51), TUGT (50) and 6 MWT (52)). The lack of high-quality studies and detailed reporting on sample size calculations and calibration procedures significantly limits the ability to draw firm conclusions.

Furthermore, many studies reported only relative reliability (ICCs) but lacked absolute reliability measures (SEM or LoA). Most studies presented only confidence intervals for variance estimates, often without comparison to appropriate benchmarks or standards (22). In line with Kobsar *et al.* (57), we recommend including sample size power estimation, calibration procedures and comprehensive statistical analyses. Both relative and absolute reliability should be assessed, and additional metrics such as SEM, r and CV could provide a more comprehensive evaluation of IMU validity and reliability.

#### Strengths and limitations of IMU applications

Most studies have used standardized IMU devices to measure gait parameters, which enhance the comparability of results. However, most studies failed to report detailed IMU calibration processes, which may have affected the accuracy of the measurements. Although there are currently no guidelines in the field of sports science regarding the use of IMUs, several key factors have been recommended for inclusion in the checklist, including sensor mass and dimension, sampling frequency, sensor capacity, sensor placement, etc. (90). In addition, different studies have differences in the selection of parameters. Some studies only focus on walking speed or steps count, while other studies include multiple parameters such as step frequency and step length. This difference has led to inconsistencies between studies, especially in terms of gait variability.

#### Selection bias and generalizability

The included studies may have had selection bias in participant recruitment, as they only selected healthy older adults who were able to complete all the tests and excluded individuals with reduced mobility or cognitive impairments. This selection bias may have led to overly optimistic results, underestimating the applicability of IMUs to the wider population. Therefore, the results of these studies should be interpreted with caution regarding their potential to generalize to the wider population.

We found that studies with larger sample sizes generally reported more consistent results, especially in terms of walking speed and stride frequency. In contrast, studies with smaller sample sizes had more fluctuating results in terms of gait variability, possibly due to statistical instability due to insufficient sample sizes. In addition, there are differences in the results of gait spatiotemporal parameters reported by institutes using different IMU brands, which may be related to the accuracy and calibration methods of the devices. Therefore, the effect of sample size and device selection on the results should be taken into account when interpreting these results.

Most studies primarily focused on community-dwelling older adults, making their results highly applicable to this population. However, the applicability of these findings is limited when it comes to hospitalized older adults or those with severe chronic conditions, restricting the generalizability of the results. In addition, the studies included in this review were conducted in controlled laboratory settings, which may not fully reflect how IMUs perform in everyday life. Therefore, when applying these results to clinical practice, the limitations of the study setting should be taken into account and the effects of IMUs in the real world should be further validated.

### Qualitative analysis

#### Validity analysis

Given the heterogeneity of test conditions, we conducted subgroup analyses on walking speed, IMU placement and visual conditions during static balance tests to assess their impact on reliability and validity. For most parameters, movement speed did not affect validity. However, at slower walking speeds, IMUs showed better agreement with gold standards for walking speed, step time and step length, likely due to reduced movement artifacts and signal noise, consistent with previous research (91). Subgroup analysis of test-retest reliability did not reveal significant differences across movement speeds. Regarding IMU placement, the locations did not generally impact validity. IMUs placed on the back showed higher agreement than those on the foot/ankle, except for step time validity. Surprisingly, the reliability of IMUs placed on the back was lower than those on the shank. These inconsistencies may arise from insufficient quantitative synthesis across studies to clarify the effects of IMU placement on gait and mobility outcomes. Previous studies (92, 93) suggest that foot, tibia and lumbar spine placements are suitable for reliable stride data, indicating that placement may not be a critical factor. Further research is needed to better understand the impact of IMU placement on data accuracy.

Twenty-one studies (34, 38, 39, 42, 45, 49, 54, 59, 60, 61, 62, 66, 72, 74, 75, 78, 79, 80, 87, 89) assessed the validity of IMUs for measuring spatiotemporal and biomechanical gait parameters. Of these, ten spatiotemporal outcomes met the statistical criteria for data pooling. The high heterogeneity (*I*^2^ = 0–98%) observed in gait spatiotemporal outcomes could be attributed to variability in test conditions, including differences in IMU brand, sensor placement and walking conditions (speed, distance and surface type). For the spatiotemporal outcomes – walking speed, cadence, step time, step time variability, stride time, step length and stride length – IMUs demonstrated excellent (summary ICCs ≥0.954) to good-to-excellent (summary r ≥ 0.869) agreement with gold standard measurements. These results, supported by generally limited to moderate evidence, suggest that IMUs may serve as a viable alternative to motion capture systems for accurately measuring gait parameters in older adults (57).

Four studies (44, 69, 73, 88) assessed the validity of IMUs for static balance outcomes, but no data pooling was possible due to outcome inconsistencies. Moderate-to-strong validity was observed for IMUs in static balance measurement. De Groote *et al.* (44) reported moderate correlations between smartphone and force plate parameters with mean and RMS acceleration during postural stability tests. Pooranawatthanakul *et al.* (73) found moderate-to-excellent validity for RMS acceleration during double-leg stance. Ozinga *et al.* (69) showed good-to-excellent correlations between an iPad and motion capture systems for postural stability. Although no quantitative synthesis was performed, existing evidence supports the potential of IMUs for static balance testing in older adults. Further high-quality studies are needed to confirm these findings.

Nine studies (31, 36, 40, 41, 50, 64, 68, 77, 83) assessed the validity of IMUs during the STS test. Three biomechanical outcomes showed sufficient statistical qualifications for data pooling, with low-to-moderate heterogeneity (*I*^2^ = 0–55%). For the TUGT, six outcomes were extracted from five studies (41, 50, 56, 62, 84), with good-to-excellent agreement for duration (summary *r* = 0.989 for STS, *r* = 0.959 for TUGT), but moderate-to-good agreement for STS mean power and velocity, likely due to differences in calculation methods between IMUs and gold standards. In the 6 MWT, five spatiotemporal outcomes were extracted from three studies (37, 46, 57), but no data pooling was possible due to outcome inconsistencies. Despite limited evidence, existing analysis indicated good-to-excellent agreement for most outcomes, suggesting that IMUs could be useful for monitoring of older adults’ functional activity during daily life.

#### Reliability analysis

A total of 28 spatiotemporal outcomes were extracted from eleven studies (34, 35, 38, 55, 56, 59, 60, 62, 67, 74, 75) assessing IMU reliability across different walking conditions. Fourteen outcomes met the criteria for data pooling. Walking speed, cadence, step time, stride time, stance time, swing time, step length and stride length showed excellent test-retest reliability (summary ICCs ≥0.941). Swing/stance time variability demonstrated good reliability (summary ICCs ≥0.757), while step time variability, step time asymmetry, stride time SD and stride time variability showed moderate reliability (summary ICCs ≥0.501). Consistent with previous research (57), time/length-related outcomes exhibited better consistency than variance-related outcomes. This may be due to mean-based gait measures being more stable and robust than variance estimates, supporting the recommendation by Lord *et al.* (94) to collect at least 12 steps.

Ten biomechanical outcomes were extracted from eight studies (32, 44, 58, 63, 70, 71, 73, 81) assessing IMU reliability during various balance tasks. Two outcomes met the criteria for data pooling. RMS acceleration during double-leg stance showed good agreement (summary ICC = 0.774), while semi-tandem stance showed moderate agreement (summary ICC = 0.749). Traditionally, center of pressure (force plates) and center of mass (motion capture) are used for postural control measurement. IMU measurement errors are influenced by sensor algorithms, unit specifications (e.g., amplitude/frequency), placement, orientation and movement types (95). Despite these limitations, IMUs remain a rapidly advancing and valuable tool for monitoring postural control.

Twelve biomechanical outcomes from five studies (40, 51, 64, 76, 86) assessed IMU reliability during the STS test. Six outcomes met the criteria for data pooling. The individual and total STS duration, stand-up duration and maximal velocity showed good agreement (summary ICCs ≥0.770), while maximal jerk and peak power showed moderate agreement (summary ICCs ≥0.716). For the TUGT, TUGT duration from three studies (62, 76, 82, 86) demonstrated excellent agreement (summary ICC = 0.935). In the 6 MWT, walking speed and stride length showed excellent or good agreement across five outcomes from four studies (37, 46, 52, 85). Despite the generally high-reliability of IMU measures, discrepancies between IMU-derived data and gold standards (e.g. force plates or motion capture systems) in functional tests such as STS and TUGT highlight the need for standardized protocols and calibration to ensure consistent results.

In conclusion, while IMUs demonstrate strong potential for gait analysis, balance and functional mobility assessment, more robust and standardized studies are needed to optimize their application. Future research should prioritize large, well-powered studies, standardization of IMUs placement and calibration and further validation in real-world settings to ensure that these devices can be confidently utilized in clinical practice.

### Limitations

This study has some limitations. First, it did not include the identification of key gait events, such as initial contact and toe-off, which are important for interpreting the results. While this review did not delve into the specifics of IMU algorithms, future studies should explore the impact of different sensor algorithms on reliability and validity, as variations in data processing approaches may substantially affect outcomes. Future research could focus on analyzing algorithmic differences between IMUs to better understand their mechanisms in sports science. Given the inconsistencies across biomechanical measures in functional mobility tests (e.g. STS and TUGT), it is important for future research to standardize outcome measures and test conditions to ensure more reliable comparisons.

## Conclusion

This systematic review and meta-analysis demonstrated that IMUs exhibit excellent validity and reliability for measuring mean spatiotemporal outcomes during gait. However, caution was warranted when using IMUs to assess spatiotemporal variability and asymmetry metrics. In the context of static balance and functional mobility tests, while biomechanical outcomes did not consistently achieve high validity and reliability, the evidence supported the use of IMUs for assessing static balance and functional mobility performance in older adults.

It was important to note that these conclusions are based on evidence from studies that are limited in number and highly heterogeneous. Therefore, factors such as IMU placement and the specific tasks being measured should be carefully considered when interpreting the results. Future research should aim to refine IMU application based on the recommendations of this review. Such efforts could improve the detection and early warning of mobility issues in real-world settings and provide valuable insights for clinicians and sports health practitioners.

## Supplementary materials



## ICMJE Statement of interest

No author has any other financial, personal or professional relationships that could be perceived as a potential conflict of interest.

## Funding Statement

This work was funded by the Research and Innovation Grant for Graduate Students, Shanghai University of Sporthttps://doi.org/10.13039/501100002397 (project no. YJSCX-2023-016).

## Author contribution statement

Conceptualization was done by LY and XX. Methodology was given by RW, FL, YW and YL. LY helped with software. Validation and formal analysis was done by XX and FL. Data curation was done by LY. LY helped in writing the original manuscript. All authors have read and agreed to the published version of the manuscript.
