# Supplementary material for: Validity and reliability of inertial measurement units on gait, static balance and functional mobility performance among community-dwelling older adults: a systematic review and meta-analysis
Source: EFORT Open Rev. 2025 Apr 1;10(4):172–85. doi: 10.1530/EOR-2024-0088 (PMC12002737; doi:10.1530/EOR-2024-0088)
Supplement: Supplementary file 1 [file supplementary_materials.pdf]

PubMed:

(((((((Wearable Electronic Devices[MeSH Terms]) OR (((((((((((Device, Wearable Electronic[Title/Abstract]) OR (Electronic Device, Wearable[Title/Abstract])) OR (Wearable Electronic Device[Title/Abstract])) OR (Wearable Technology[Title/Abstract])) OR (Technology, Wearable[Title/Abstract])) OR (Wearable Technologies[Title/Abstract])) OR (Wearable Devices[Title/Abstract])) OR (Device, Wearable[Title/Abstract])) OR (Wearable Device[Title/Abstract])) OR (Electronic Skin[Title/Abstract])) OR (Skin, Electronic[Title/Abstract]))) OR ((Micro-Electrical-Mechanical Systems[MeSH Terms]) OR ((((((((((((microelectromechanical system(Embase[Title/Abstract])) OR (Micro Electrical Mechanical Systems[Title/Abstract])) OR (Micro-Electrical-Mechanical System[Title/Abstract])) OR (System, Micro-Electrical-Mechanical[Title/Abstract])) OR (Systems, Micro-Electrical-Mechanical[Title/Abstract])) OR (MEMS[Title/Abstract])) OR (Micro-Electro-Mechanical Systems[Title/Abstract])) OR (Micro Electro Mechanical Systems[Title/Abstract])) OR (Micro-Electro-Mechanical System[Title/Abstract])) OR (System, Micro-Electro-Mechanical[Title/Abstract])) OR (Systems, Micro-Electro-Mechanical[Title/Abstract])) OR (BioMEMS[Title/Abstract])))) OR ((Smartphone[MeSH Terms]) OR ((((Smartphones[Title/Abstract]) OR (Smart Phones[Title/Abstract])) OR (Smart Phone[Title/Abstract])) OR (Phones, Smart[Title/Abstract])))) OR (((((((((wearable sensor*[Title/Abstract]) OR (inertial sensor*[Title/Abstract])) OR (inertial motion capture[Title/Abstract])) OR (inertial measurement unit*[Title/Abstract])) OR (IMU[Title/Abstract])) OR (IMUs[Title/Abstract])) OR (acceleromet*[Title/Abstract])) OR (gyroscop*[Title/Abstract])) OR (magnetomet*[Title/Abstract]))) AND ((((Spatio-Temporal Analysis[MeSH Terms]) OR ((((((((((((((((((Analyses, Spatio-Temporal[Title/Abstract]) OR (Analysis, Spatio-Temporal[Title/Abstract])) OR (Spatio Temporal Analysis[Title/Abstract])) OR (Spatio-Temporal Analyses[Title/Abstract])) OR (Spatiotemporal Analysis[Title/Abstract])) OR (Analyses, Spatiotemporal[Title/Abstract])) OR (Analysis, Spatiotemporal[Title/Abstract])) OR (Spatiotemporal Analyses[Title/Abstract])) OR (Spatial Temporal Analysis[Title/Abstract])) OR (Analyses, Spatial Temporal[Title/Abstract])) OR (Analysis, Spatial Temporal[Title/Abstract])) OR (Spatial Temporal Analyses[Title/Abstract])) OR (Temporal Analyses, Spatial[Title/Abstract])) OR (Temporal Analysis, Spatial[Title/Abstract])) OR (Space-Time Geography[Title/Abstract])) OR (Geographies, Space-Time[Title/Abstract])) OR (Geography, Space-Time[Title/Abstract])) OR (Space Time Geography[Title/Abstract])) OR (Space-Time Geographies[Title/Abstract]))) OR ((Postural Balance[MeSH Terms]) OR (((((((((((((((((body equilibrium[Title/Abstract]) OR (Posture Equilibrium[Title/Abstract])) OR (Equilibrium, Posture[Title/Abstract])) OR (Posture Equilibriums[Title/Abstract])) OR (Balance, Postural[Title/Abstract])) OR (Postural Equilibrium[Title/Abstract])) OR (Equilibrium, Postural[Title/Abstract])) OR (Posture Balance[Title/Abstract])) OR (Balance, Posture[Title/Abstract])) OR (Posture Balances[Title/Abstract])) OR (Musculoskeletal Equilibrium[Title/Abstract])) OR (Equilibrium, Musculoskeletal[Title/Abstract])) OR (Postural Control[Title/Abstract])) OR (Control, Postural[Title/Abstract])) OR (Postural Controls[Title/Abstract])) OR (Posture Control[Title/Abstract])) OR (Control, Posture[Title/Abstract])) OR (Posture Controls[Title/Abstract])))) OR ((((((((((((((((((((((((((((((((((((gait*[Title/Abstract]) OR (cadence[Title/Abstract])) OR (step

frequency[Title/Abstract])) OR (stride frequency[Title/Abstract])) OR (step time[Title/Abstract])) OR (stride time[Title/Abstract])) OR (cycle time[Title/Abstract])) OR (contact time[Title/Abstract])) OR (swing time[Title/Abstract])) OR (step length[Title/Abstract])) OR (stride length[Title/Abstract])) OR (sit-to-stand[Title/Abstract])) OR (chair-stand[Title/Abstract])) OR (chair-rise[Title/Abstract])) OR ("timed up and go"[Title/Abstract])) OR (Timed-Up-and-Go[Title/Abstract])) OR ("Timed-up and Go"[Title/Abstract])) OR (Timed Up & Go[Title/Abstract])) OR (Timed-Up-&-Go[Title/Abstract])) OR (Timed Up-and-Go[Title/Abstract])) OR (Timed Up-&-Go[Title/Abstract])) OR ("Get Up and Go"[Title/Abstract])) OR (TUG[Title/Abstract])) OR (TUGT[Title/Abstract])) OR (six min walk[Title/Abstract])) OR (six minute walk[Title/Abstract])) OR (six-min walk[Title/Abstract])) OR (six-minute walk[Title/Abstract])) OR (6 min walk[Title/Abstract])) OR (6 minute walk[Title/Abstract])) OR (6-min walk[Title/Abstract])) OR (6-minute walk[Title/Abstract])) OR (6MW[Title/Abstract])) OR (6MWT[Title/Abstract])) OR (6-MW[Title/Abstract])) OR (6-MWT[Title/Abstract])))) AND ((((aged[Title/Abstract]) OR (aging[Title/Abstract])) OR (elder*[Title/Abstract])) OR (older adult*[Title/Abstract]))) AND ((((Reproducibility of Results[MeSH Terms]) OR ((((((((((((((((((((((((((((reproducibility[Title/Abstract]) OR (Reproducibility of Findings[Title/Abstract])) OR (Reproducibility Of Result[Title/Abstract])) OR (Of Result, Reproducibility[Title/Abstract])) OR (Of Results, Reproducibility[Title/Abstract])) OR (Result, Reproducibility Of[Title/Abstract])) OR (Results, Reproducibility Of[Title/Abstract])) OR (Reproducibility of Finding[Title/Abstract])) OR (Finding Reproducibilities[Title/Abstract])) OR (Finding Reproducibility[Title/Abstract])) OR (Reliability of Results[Title/Abstract])) OR (Reliability of Result[Title/Abstract])) OR (Result Reliabilities[Title/Abstract])) OR (Result Reliability[Title/Abstract])) OR (Reliability (Epidemiology[Title/Abstract]))) OR (Validity (Epidemiology[Title/Abstract]))) OR (Validity of Results[Title/Abstract])) OR (Validity of Result[Title/Abstract])) OR (Result Validities[Title/Abstract])) OR (Result Validity[Title/Abstract])) OR (Face Validity[Title/Abstract])) OR (Validity, Face[Title/Abstract])) OR (Reliability[Title/Abstract] AND Validity[Title/Abstract])) OR (Validity[Title/Abstract] AND Reliability[Title/Abstract])) OR (Test-Retest Reliability[Title/Abstract])) OR (Reliabilities, Test-Retest[Title/Abstract])) OR (Reliability, Test-Retest[Title/Abstract])) OR (Test Retest Reliability[Title/Abstract]))) OR ((Data Accuracy[MeSH Terms]) OR (((((((Accuracies, Data[Title/Abstract]) OR (Accuracy, Data[Title/Abstract])) OR (Data Accuracies[Title/Abstract])) OR (Data Quality[Title/Abstract])) OR (Data Qualities[Title/Abstract])) OR (Qualities, Data[Title/Abstract])) OR (Quality, Data[Title/Abstract])))) OR (((((validity[Title/Abstract]) OR (reliability[Title/Abstract])) OR (feasibility[Title/Abstract])) OR (repeatability[Title/Abstract])) OR (consistency[Title/Abstract])))

Embase:

#26. #8 AND #16 AND #24 AND #25

#25. 'aged':ab,ti OR 'aging':ab,ti OR 'elder*':ab,ti OR 'older adult':ab,ti

#24. #19 OR #22 OR #23

#23. 'validity':ab,ti OR 'reliability':ab,ti OR 'feasibility':ab,ti OR 'repeatability':ab,ti OR 'consistency':ab,ti OR 'accuracy':ab,ti

#22. #20 OR #21

#21. 'accuracies, data':ab,ti OR 'accuracy, data':ab,ti OR 'data accuracies':ab,ti OR 'data quality':ab,ti OR 'data qualities':ab,ti OR 'qualities, data':ab,ti OR 'quality, data':ab,ti

#20. 'data accuracy'/exp

#19. #17 OR #18

#18. 'reproducibility of results':ab,ti OR 'reproducibility of findings':ab,ti OR 'reproducibility of result':ab,ti OR 'of result, reproducibility':ab,ti OR 'of results, reproducibility':ab,ti OR 'result, reproducibility of':ab,ti OR 'results, reproducibility of':ab,ti OR 'reproducibility of finding':ab,ti OR 'finding reproducibilities':ab,ti OR 'finding reproducibility':ab,ti OR 'reliability of results':ab,ti OR 'reliability of result':ab,ti OR 'result reliabilities':ab,ti OR 'result reliability':ab,ti OR 'reliability (epidemiology)':ab,ti OR 'validity (epidemiology)':ab,ti OR 'validity of results':ab,ti OR 'validity of result':ab,ti OR 'result validities':ab,ti OR 'result validity':ab,ti OR 'face validity':ab,ti OR 'validity, face':ab,ti OR 'reliability and validity':ab,ti OR 'validity and reliability':ab,ti OR 'test-retest reliability':ab,ti OR 'reliabilities, test-retest':ab,ti OR 'reliability, test-retest':ab,ti OR 'test retest reliability':ab,ti

#17. 'reproducibility'/exp

#16. #11 OR #14 OR #15

#15. 'gait*':ab,ti OR 'cadence':ab,ti OR 'step frequency':ab,ti OR 'stride frequency':ab,ti OR 'step time':ab,ti OR 'cycle time':ab,ti OR 'contact time':ab,ti OR 'swing time':ab,ti OR 'step length':ab,ti OR 'stride length':ab,ti OR 'sit-to-stand':ab,ti OR 'chair-stand':ab,ti OR 'chair-rise':ab,ti OR 'timed up and go':ab,ti OR 'timed-up-and-go':ab,ti OR 'timed-up and go':ab,ti OR 'timed up & go':ab,ti OR 'timed-up-&-go':ab,ti OR 'timed up-and-go':ab,ti OR 'timed up-&-go':ab,ti OR 'get up and go':ab,ti OR 'tug':ab,ti OR 'tugt':ab,ti OR 'six min walk':ab,ti OR 'six minute walk':ab,ti OR 'six-min walk':ab,ti OR 'six-minute walk':ab,ti OR '6 min walk':ab,ti OR '6 minute walk':ab,ti OR '6-min walk':ab,ti OR '6-minute walk':ab,ti OR '6mw':ab,ti OR '6mwt':ab,ti OR '6-mw':ab,ti OR '6-mwt':ab,ti

#14. #12 OR #13

#13. 'postural balance':ab,ti OR 'posture equilibrium':ab,ti OR 'equilibrium, posture':ab,ti OR 'posture equilibriums':ab,ti OR 'balance, postural':ab,ti OR 'postural equilibrium':ab,ti OR 'equilibrium, postural':ab,ti OR 'posture balance':ab,ti OR 'balance, posture':ab,ti OR 'posture balances':ab,ti OR 'musculoskeletal equilibrium':ab,ti OR 'equilibrium, musculoskeletal':ab,ti OR 'postural control':ab,ti OR 'control, postural':ab,ti OR 'postural controls':ab,ti OR 'posture control':ab,ti OR 'control, posture':ab,ti OR 'posture controls':ab,ti

#12. 'body equilibrium'/exp

#11. #9 OR #10

#10. 'spatio-temporal analysis':ab,ti OR 'analyses, spatio-temporal':ab,ti OR 'analysis, spatio-temporal':ab,ti OR 'spatio temporal analysisspatiotemporal analysis':ab,ti OR 'spatio-temporal analyses':ab,ti OR 'spatiotemporal analysis':ab,ti OR 'analyses, spatiotemporal':ab,ti OR 'analysis, spatiotemporal':ab,ti OR 'spatiotemporal analyses':ab,ti OR 'spatial temporal analysis':ab,ti OR 'analyses, spatial temporal':ab,ti OR 'analysis, spatial temporal':ab,ti OR 'spatial temporal analyses':ab,ti OR 'temporal analyses, spatial':ab,ti OR 'temporal analysis, spatial':ab,ti OR 'space-time geography':ab,ti OR 'geographies, space-time':ab,ti OR 'geography, space-time':ab,ti OR 'space time geography':ab,ti OR 'space-time geographies':ab,ti

#9. 'spatiotemporal analysis'/exp

#8. #3 OR #6 OR #7

#7. 'wearable electronic devices':ab,ti OR 'device, wearable electronic':ab,ti OR 'electronic device, wearable':ab,ti OR 'wearable electronic device':ab,ti OR 'wearable technology':ab,ti OR 'technology, wearable':ab,ti OR 'wearable technologies':ab,ti OR 'wearable devices':ab,ti OR 'device, wearable':ab,ti OR 'wearable device':ab,ti OR 'electronic skin':ab,ti OR 'skin, electronic':ab,ti OR 'wearable sensor*':ab,ti OR 'inertial sensor*':ab,ti OR 'inertial motion capture':ab,ti OR 'inertial measurement unit*':ab,ti OR 'imu':ab,ti OR 'imus':ab,ti OR 'acceleromet*':ab,ti OR 'gyroscop*':ab,ti OR 'magnetomet*':ab,ti

#6. #4 OR #5

#5. 'smartphones':ab,ti OR 'smart phones':ab,ti OR 'smart phone':ab,ti OR 'phones, smart':ab,ti

#4. 'smartphone'/exp

#3. #1 OR #2

#2. 'micro-electrical-mechanical systems':ab,ti OR 'micro electrical mechanical systems':ab,ti OR 'micro-electrical-mechanical system':ab,ti OR 'system, micro-electrical-mechanical':ab,ti OR 'systems, micro-electrical-mechanical':ab,ti OR 'mems':ab,ti OR 'micro-electro-mechanical systems':ab,ti OR 'micro electro mechanical systems':ab,ti OR 'micro-electro-mechanical system':ab,ti OR 'system, micro-electro-mechanical':ab,ti OR 'systems, micro-electro-mechanical':ab,ti OR 'biomems':ab,ti

#1. 'microelectromechanical system'/exp

Scopus:

((TITLE-ABS-KEY ("wearable electronic devices" OR "device, wearable electronic" OR "electronic device, wearable" OR "wearable electronic device" OR "wearable technology" OR "technology, wearable" OR "wearable technologies" OR "wearable devices" OR "device, wearable" OR "wearable device" OR "electronic skin" OR "skin, electronic") OR TITLE-ABS-KEY ("wearable sensor*" OR "inertial sensor*" OR "inertial motion capture" OR "inertial measurement unit*" OR "imu" OR "imus" OR "acceleromet*" OR "gyroscop*" OR "magnetomet*") OR TITLE-ABS-KEY ("micro-electrical-mechanical systems" OR "micro electrical mechanical systems" OR "micro-electrical-mechanical system" OR "system, micro-electrical-mechanical" OR "systems, micro-electrical-mechanical" OR "mems" OR "micro-electro-mechanical systems" OR "micro electro mechanical systems" OR "micro-electro-mechanical system" OR "system, micro-electro-mechanical" OR "systems, micro-electro-mechanical" OR "biomems") OR TITLE-ABS-KEY ("smartphone" OR "smartphones" OR "smart phones" OR "smart phone" OR "phones, smart"))) AND ((TITLE-ABS-KEY ("spatio-temporal analysis" OR "analyses, spatio-temporal" OR "analysis, spatio-temporal" OR "spatio temporal analysisspatiotemporal analysis" OR "spatio-temporal analyses" OR "spatiotemporal analysis" OR "analyses, spatiotemporal" OR "analysis, spatiotemporal" OR "spatiotemporal analyses" OR "spatial temporal analysis" OR "analyses, spatial temporal" OR "analysis, spatial temporal" OR spatial AND temporal AND analyses "temporal analyses, spatial" OR "temporal analysis, spatial" OR "space-time geography" OR "geographies, space-time" OR "geography, space-time" OR "space time geography" OR "space-time geographies") OR TITLE-ABS-KEY ("postural balance" OR "posture equilibrium" OR "equilibrium, posture" OR "posture equilibriums" OR "balance, postural" OR "postural equilibrium" OR "equilibrium, postural" OR "posture balance" OR "balance, posture" OR "posture balances" OR "musculoskeletal equilibrium" OR "equilibrium, musculoskeletal" OR "postural control" OR "control, postural" OR "postural controls" OR "posture

control" OR "control, posture") OR TITLE-ABS-KEY ("gait*" OR "cadence" OR "step frequency" OR "stride frequency" OR "step time" OR "cycle time" OR "contact time" OR "swing time" OR "step length" OR "stride length" OR "sit-to-stand" OR "chair-stand" OR "chair-rise" OR "timed up and go" OR "timed-up-and-go" OR "timed-up and go" OR "timed up & go" OR "timed-up-&-go" OR "timed up-and-go" OR "timed up-&-go" OR "get up and go" OR "tug" OR "tugt" OR "six min walk" OR "six minute walk" OR "six-min walk" OR "six-minute walk" OR "6 min walk" OR "posture controls" OR "6 minute walk" OR "6-min walk" OR "6-minute walk" OR "6mw" OR "6mwt" OR "6-mw" OR "6-mwt"))) AND ((TITLE-ABS-KEY ("reproducibility of results" OR "reproducibility of findings" OR "reproducibility of result" OR "of result, reproducibility" OR "of results, reproducibility" OR "result, reproducibility of" OR "results, reproducibility of" OR "reproducibility of finding" OR "finding reproducibilities" OR "finding reproducibility" OR "reliability of results" OR "reliability of result" OR "result reliability" OR "reliability (epidemiology)" OR "validity (epidemiology)" OR "validity of results" OR "validity of result" OR "result validities" OR "result validity" OR "face validity" OR "validity, face" OR "reliability and validity" OR "validity and reliability" OR "test-retest reliability" OR "reliabilities, test-retest" OR "reliability, test-retest" OR "test retest reliability") OR TITLE-ABS-KEY ("data accuracy" OR "accuracies, data" OR "accuracy, data" OR "data accuracies" OR "data quality" OR "data qualities" OR "qualities, data" OR "quality, data") OR TITLE-ABS-KEY ("validity" OR "reliability" OR "feasibility " OR "repeatability" OR "consistency" OR "accuracy"))) AND (TITLE-ABS-KEY ("aged" OR "aging" OR "elder*" OR "older adult*"))

Cochrane library:

#1 MeSH descriptor: [Wearable Electronic Devices] explode all trees

#2 (Wearable Electronic Devices):ti,ab,kw OR (Device, Wearable Electronic):ti,ab,kw OR (Electronic Device, Wearable):ti,ab,kw OR (Wearable Electronic Device):ti,ab,kw OR (Wearable Technology):ti,ab,kw OR (Technology, Wearable):ti,ab,kw OR (Wearable Technologies):ti,ab,kw OR (Wearable Devices):ti,ab,kw OR (Device, Wearable):ti,ab,kw OR (Wearable Device):ti,ab,kw OR (Electronic Skin):ti,ab,kw OR (Skin, Electronic):ti,ab,kw OR (wearable sensor*):ti,ab,kw OR (inertial sensor*):ti,ab,kw OR (inertial motion capture):ti,ab,kw OR (inertial measurement unit*):ti,ab,kw OR (IMU):ti,ab,kw OR (IMUs):ti,ab,kw OR (acceleromet*):ti,ab,kw OR (gyroscop*):ti,ab,kw OR (magnetomet*):ti,ab,kw

#3 MeSH descriptor: [Micro-Electrical-Mechanical Systems] explode all trees

#4 (Micro Electrical Mechanical Systems):ti,ab,kw OR (Micro-Electrical-Mechanical System):ti,ab,kw OR (System, Micro-Electrical-Mechanical):ti,ab,kw OR (Systems, Micro-Electrical-Mechanical):ti,ab,kw OR (MEMS):ti,ab,kw OR (Micro-Electro-Mechanical Systems):ti,ab,kw OR (Micro Electro Mechanical Systems):ti,ab,kw OR (Micro-Electro-Mechanical System):ti,ab,kw OR (System, Micro-Electro-Mechanical):ti,ab,kw OR (Systems, Micro-Electro-Mechanical):ti,ab,kw OR (BioMEMS):ti,ab,kw

#5 MeSH descriptor: [Smartphone] explode all trees

#6 (Smartphones):ti,ab,kw OR (Smart Phones):ti,ab,kw OR (Smart Phone):ti,ab,kw OR (Phones, Smart):ti,ab,kw

#7 #1 OR #2 OR #3 OR #4 OR #5 OR #6

#8 MeSH descriptor: [Spatio-Temporal Analysis] explode all trees

#9 (Analyses, Spatio-Temporal):ti,ab,kw OR (Analysis, Spatio-Temporal):ti,ab,kw OR (Spatio Temporal Analysisspatiotemporal analysis):ti,ab,kw OR (Spatio-Temporal Analyses):ti,ab,kw OR (Spatiotemporal Analysis):ti,ab,kw OR (Analyses, Spatiotemporal):ti,ab,kw OR (Analysis, Spatiotemporal):ti,ab,kw OR (Spatiotemporal Analyses):ti,ab,kw OR (Spatial Temporal Analysis):ti,ab,kw OR (Analyses, Spatial Temporal):ti,ab,kw OR (Analysis, Spatial Temporal):ti,ab,kw OR (Spatial Temporal Analyses):ti,ab,kw OR (Temporal Analyses, Spatial):ti,ab,kw OR (Temporal Analysis, Spatial):ti,ab,kw OR (Space-Time Geography):ti,ab,kw OR (Geographies, Space-Time):ti,ab,kw OR (Geography, Space-Time):ti,ab,kw OR (Space Time Geography):ti,ab,kw OR (Space-Time Geographies):ti,ab,kw

#10 #8 OR #9

#11 MeSH descriptor: [Postural Balance] explode all trees

#12 (Postural Balance):ti,ab,kw OR (Posture Equilibrium):ti,ab,kw OR (Equilibrium, Posture):ti,ab,kw OR (Posture Equilibriums):ti,ab,kw OR (Balance, Postural):ti,ab,kw OR (Postural Equilibrium):ti,ab,kw OR (Equilibrium, Postural):ti,ab,kw OR (Posture Balance):ti,ab,kw OR (Balance, Posture):ti,ab,kw OR (Posture Balances):ti,ab,kw OR (Musculoskeletal Equilibrium):ti,ab,kw OR (Equilibrium, Musculoskeletal):ti,ab,kw OR (Postural Control):ti,ab,kw OR (Control, Postural):ti,ab,kw OR (Postural Controls):ti,ab,kw OR (Posture Control):ti,ab,kw OR (Control, Posture):ti,ab,kw

#13 #11 OR #12

#14 (gait*):ti,ab,kw OR (cadence):ti,ab,kw OR (step frequency):ti,ab,kw OR (stride frequency):ti,ab,kw OR (step time):ti,ab,kw OR (cycle time):ti,ab,kw OR (contact time):ti,ab,kw OR (swing time):ti,ab,kw OR (step length):ti,ab,kw OR (stride length):ti,ab,kw OR (sit-to-stand):ti,ab,kw OR (chair-stand):ti,ab,kw OR (chair-rise):ti,ab,kw OR (timed up and go):ti,ab,kw OR (Timed-up and Go):ti,ab,kw OR (Timed Up & Go):ti,ab,kw OR (Get Up and Go):ti,ab,kw OR (TUG):ti,ab,kw OR (TUGT):ti,ab,kw OR (six min walk):ti,ab,kw OR (six minute walk):ti,ab,kw OR (six-min walk):ti,ab,kw OR (six-minute walk):ti,ab,kw OR (6 min walk):ti,ab,kw OR (6 minute walk):ti,ab,kw OR (6MW):ti,ab,kw OR (6MWT):ti,ab,kw

#15 #10 OR #13 OR #14

#16 (aged):ti,ab,kw OR (aging):ti,ab,kw OR (elder*):ti,ab,kw OR (older adult):ti,ab,kw

#17 MeSH descriptor: [Reproducibility of Results] explode all trees

#18 (Reproducibility of Findings):ti,ab,kw OR (Reproducibility Of Result):ti,ab,kw OR (Of Result, Reproducibility):ti,ab,kw OR (Of Results, Reproducibility):ti,ab,kw OR (Result, Reproducibility Of):ti,ab,kw OR (Results, Reproducibility Of):ti,ab,kw OR (Reproducibility of Finding):ti,ab,kw OR (Finding Reproducibilities):ti,ab,kw OR (Finding Reproducibility):ti,ab,kw OR (Reliability of Results):ti,ab,kw OR (Reliability of Result):ti,ab,kw OR (Result Reliabilities):ti,ab,kw OR (Result Reliability):ti,ab,kw OR (Reliability (Epidemiology)):ti,ab,kw OR (Validity (Epidemiology)):ti,ab,kw OR (Validity of Results):ti,ab,kw OR (Validity of Result):ti,ab,kw OR (Result Validities):ti,ab,kw OR (Result Validity):ti,ab,kw OR (Face Validity):ti,ab,kw OR (Validity, Face):ti,ab,kw OR (Reliability and Validity):ti,ab,kw OR (Validity and Reliability):ti,ab,kw OR (Test-Retest Reliability):ti,ab,kw OR (Reliabilities, Test-Retest):ti,ab,kw OR (Reliability, Test-Retest):ti,ab,kw OR (Test Retest Reliability):ti,ab,kw

#19 #17 OR #18

#20 MeSH descriptor: [Data Accuracy] explode all trees

#21 (Accuracies, Data):ti,ab,kw OR(Accuracy, Data):ti,ab,kw OR (Data Accuracies):ti,ab,kw OR (Data Quality):ti,ab,kw OR (Data Qualities):ti,ab,kw OR (Qualities, Data):ti,ab,kw OR(Quality, Data):ti,ab,kw

#22 #20 OR #21

#23 (validity):ti,ab,kw OR (reliability):ti,ab,kw OR (feasibility):ti,ab,kw OR(repeatability):ti,ab,kw OR(consistency):ti,ab,kw OR (accuracy):ti,ab,kw

#24 #19 OR #22 OR #23

#25 #7 AND #15 AND #16 AND #24

Ovid MEDLINE:

1 (Wearable Electronic Devices or Device, Wearable Electronic or Electronic Device, Wearable or Wearable Electronic Device or Wearable Technology or Technology, Wearable or Wearable Technologies or Wearable Devices or Wearable Device or Electronic Skin or Skin, Electronic).ti,ab,kw.

2 (wearable sensor* or inertial sensor* or inertial motion capture or inertial measurement unit* or IMU or IMUs or acceleromet* or gyroscop* or magnetomet*).ti,ab,kw.

3 (Micro-Electrical-Mechanical Systems or Micro Electrical Mechanical Systems or Micro-Electrical-Mechanical System or System, Micro-Electrical-Mechanical or Systems, Micro-Electrical-Mechanical or MEMS or Micro-Electro-Mechanical Systems or Micro Electro Mechanical Systems or Micro-Electro-Mechanical System or System, Micro-Electro-Mechanical or Systems, Micro-Electro-Mechanical or BioMEMS).ti,ab,kw.

4 (Smartphone or Smartphones or Smart Phones or Smart Phone or Phones, Smart).ti,ab,kw.

5 1 or 2 or 3 or 4

6 (Spatio-Temporal Analysis or Analyses, Spatio-Temporal or Analysis, Spatio-Temporal or Spatio Temporal Analysisspatiotemporal analysis or Spatio-Temporal Analyses or Spatiotemporal Analysis or Analyses, Spatiotemporal or Analysis, Spatiotemporal or Spatiotemporal Analyses or Spatial Temporal Analysis or Analyses, Spatial Temporal or Analysis, Spatial Temporal or Spatial Temporal Analyses or Temporal Analyses, Spatial or Temporal Analysis, Spatial or Space-Time Geography or Geographies, Space-Time or Geography, Space-Time or Space Time Geography or Space-Time Geographies).ti,ab,kw.

7 (Postural Balance or Posture Equilibrium or Equilibrium, Posture or Posture Equilibriums or Balance, Postural or Postural Equilibrium or Equilibrium, Postural or Posture Balance or Balance, Posture or Posture Balances or Musculoskeletal Equilibrium or Equilibrium, Musculoskeletal or Postural Control or Control, Postural or Postural Controls or Posture Control or Control, Posture).ti,ab,kw.

8 (((((((gait* or cadence or step frequency or stride frequency or step time or cycle time or contact time or swing time or step length or stride length or sit-to-stand or chair-stand or chair-rise or timed up) and go) or Timed-Up-and-Go or Timed-up) and Go) or Timed Up & Go or Timed-Up-&-Go or Timed Up-and-Go or Timed Up-&-Go or Get Up) and Go) or TUG or TUGT or six min walk or six minute walk or six-min walk or six-minute walk or 6 min walk or Posture Controls or 6 minute walk or 6-min walk or 6-minute walk or 6MW or 6MWT or 6-MW or 6-MWT).ti,ab,kw.

9 (((((((gait* or cadence or step frequency or stride frequency or step time or cycle time or contact time or swing time or step length or stride length or sit-to-stand or chair-stand or chair-rise or timed up) and go) or Timed-Up-and-Go or Timed-up) and Go) or Timed Up & Go or Timed-Up-&-Go or Timed Up-and-Go or Timed Up-&-Go or Get Up) and Go) or TUG or TUGT or six min walk or six minute

walk or six-min walk or six-minute walk or 6 min walk or Posture Controls or 6 minute walk or 6-min walk or 6-minute walk or 6MW or 6MWT or 6-MW or 6-MWT).ti,ab,kw.

10 6 or 7 or 8

11 (((((Reproducibility of Results or Reproducibility of Findings or Reproducibility Of Result or Of Result, Reproducibility or Of Results, Reproducibility or Result, Reproducibility Of or Results, Reproducibility Of or Reproducibility of Finding or Finding Reproducibilities or Finding Reproducibility or Reliability of Results or Reliability of Result or Result ReliabilitiesResult Reliability or Validity of Results or Validity of Result or Result Validities or Result Validity or Face Validity or Validity, Face or Reliability) and Validity) or Validity) and Reliability) or Test-Retest Reliability or Reliabilities, Test-Retest or Reliability, Test-Retest or Test Retest Reliability).ti,ab,kw.

12 (Data Accuracy or Accuracies, Data or Accuracy, Data or Data Accuracies or Data Quality or Data Qualities or Qualities, Data or Quality, Data).ti,ab,kw.

13 (validity or reliability or feasibility or repeatability or consistency or accuracy).ti,ab,kw.

14 10 or 11 or 12

15 (aged or aging or elder* or older adult*).ti,ab,kw.

16 5 and 9 and 13 and 14

WOS

#1 ((((((((((TS=(Wearable Electronic Devices)) OR TS=(Device, Wearable Electronic)) OR TS=(Electronic Device, Wearable)) OR TS=(Wearable Electronic Device)) OR TS=(Wearable Technology)) OR TS=(Technology, Wearable)) OR TS=(Wearable Technologies)) OR TS=(Wearable Devices)) OR TS=(Device, Wearable)) OR TS=(Wearable Device)) OR TS=(Electronic Skin)) OR TS=(Skin, Electronic) **and** Preprint Citation Index **(Exclude – Database)**

#2 ((((((((((TS=(Micro-Electrical-Mechanical Systems)) OR TS=(Micro Electrical Mechanical Systems)) OR TS=(Micro-Electrical-Mechanical System)) OR TS=(System, Micro-Electrical-Mechanical)) OR TS=(Systems, Micro-Electrical-Mechanical)) OR TS=(MEMS)) OR TS=(Micro-Electro-Mechanical Systems)) OR TS=(Micro Electro Mechanical Systems)) OR TS=(Micro-Electro-Mechanical System)) OR TS=(System, Micro-Electro-Mechanical)) OR TS=(Systems, Micro-Electro-Mechanical)) OR TS=(BioMEMS) **and** Preprint Citation Index **(Exclude – Database)**

#3 ((((TS=(Smartphone)) OR TS=(Smartphones)) OR TS=(Smart Phones)) OR TS=(Smart Phone)) OR TS=(Phones, Smart) **and** Preprint Citation Index **(Exclude – Database)**

#4 (((((((((((((TS=(Smartphone)) OR TS=(Smartphones)) OR TS=(Smart Phones)) OR TS=(Smart Phone)) OR TS=(Phones, Smart)) OR TS=(wearable sensor*)) OR TS=(inertial sensor*)) OR TS=(inertial motion capture)) OR TS=(inertial measurement unit*)) OR TS=(IMU)) OR TS=(IMUs)) OR TS=(MEMS)) OR TS=(acceleromet*)) OR TS=(gyroscop*)) OR TS=(magnetomet*) **and** Preprint Citation Index **(Exclude – Database)**

#5 #1 OR #2 OR #3 OR #4 **and** Preprint Citation Index **(Exclude – Database)**

#6 ((((((((((((((((((TS=(Spatio-Temporal Analysis)) OR TS=(Analyses, Spatio-Temporal)) OR TS=(Analysis, Spatio-Temporal)) OR TS=(Spatio Temporal Analysis)) OR TS=(Spatio-Temporal Analyses)) OR TS=(Spatiotemporal Analysis)) OR TS=(Analyses, Spatiotemporal)) OR TS=(Analysis,

Spatiotemporal)) OR TS=(Spatiotemporal Analyses)) OR TS=(Spatial Temporal Analysis)) OR TS=(Analyses, Spatial Temporal)) OR TS=(Analysis, Spatial Temporal)) OR TS=(Spatial Temporal Analyses)) OR TS=(Temporal Analyses, Spatial)) OR TS=(Temporal Analysis, Spatial)) OR TS=(Space-Time Geography)) OR TS=(Geographies, Space-Time)) OR TS=(Geography, Space-Time)) OR TS=(Space Time Geography)) OR TS=(Space-Time Geographies) **and** Preprint Citation Index **(Exclude – Database)**

#7 (((((((((((((((TS=(Postural Balance)) OR TS=(Posture Equilibrium)) OR TS=(Equilibrium, Posture)) OR TS=(Posture Equilibriums)) OR TS=(Balance, Postural)) OR TS=(Postural Equilibrium)) OR TS=(Equilibrium, Postural)) OR TS=(Posture Balance)) OR TS=(Balance, Posture)) OR TS=(Posture Balances)) OR TS=(Musculoskeletal Equilibrium)) OR TS=(Equilibrium, Musculoskeletal)) OR TS=(Postural Control)) OR TS=(Control, Postural)) OR TS=(Postural Controls)) OR TS=(Posture Control)) OR TS=(Control, Posture)) OR TS=(Posture Controls) **and** Preprint Citation Index **(Exclude – Database)**

#8 ((((((((((((((((((((((((((((((((((((TS=(gait*)) OR TS=(cadence)) OR TS=(step frequency)) OR TS=(stride frequency)) OR TS=(step time)) OR TS=(stride time)) OR TS=(cycle time)) OR TS=(contact time)) OR TS=(swing time)) OR TS=(step length)) OR TS=(stride length)) OR TS=(spatiotemporal)) OR TS=(Spatio-Temporal)) OR TS=(sit-to-stand)) OR TS=(chair-stand)) OR TS=(chair-rise)) OR TS=("timed up and go")) OR TS=(Timed-Up-and-Go)) OR TS=("Timed-up and Go")) OR TS=(Timed Up & Go)) OR TS=(Timed-Up-&-Go)) OR TS=(Timed Up-and-Go)) OR TS=(Timed Up-&-Go)) OR TS=("Get Up and Go")) OR TS=(TUG)) OR TS=(TUGT)) OR TS=(six min walk)) OR TS=(six minute walk)) OR TS=(six-min walk)) OR TS=(six-minute walk)) OR TS=(6 min walk)) OR TS=(6 minute walk)) OR TS=(6-min walk)) OR TS=(6-minute walk)) OR TS=(6MW)) OR TS=(6MWT)) OR TS=(6-MW)) OR TS=(6-MWT) **and** Preprint Citation Index **(Exclude – Database)**

#9 #8 OR #7 OR #6 **and** Preprint Citation Index **(Exclude – Database)**

#10 (((TS=(aged)) OR TS=(aging)) OR TS=(elder*)) OR TS=(older adult*) **and** Preprint Citation Index **(Exclude – Database)**

#11 ((((((((((((((((((((((((((TS=(Reproducibility of Results)) OR TS=(Reproducibility of Findings)) OR TS=(Reproducibility Of Result)) OR TS=(Of Result, Reproducibility)) OR TS=(Of Results, Reproducibility)) OR TS=(Result, Reproducibility Of)) OR TS=(Results, Reproducibility Of)) OR TS=(Reproducibility of Finding)) OR TS=(Finding Reproducibilities)) OR TS=(Finding Reproducibility)) OR TS=(Reliability of Results)) OR TS=(Reliability of Result)) OR TS=(Result Reliabilities)) OR TS=(Result Reliability)) OR TS=(Reliability (Epidemiology))) OR TS=(Validity (Epidemiology))) OR TS=(Validity of Results)) OR TS=(Validity of Result)) OR TS=(Result Validities)) OR TS=(Result Validity)) OR TS=(Face Validity)) OR TS=(Validity, Face)) OR TS=(Reliability and Validity)) OR TS=(Validity and Reliability)) OR TS=(Test-Retest Reliability)) OR TS=(Reliabilities, Test-Retest)) OR TS=(Reliability, Test-Retest)) OR TS=(Test Retest Reliability) **and** Preprint Citation Index **(Exclude – Database)**

#12 (((((((TS=(Data Accuracy)) OR TS=(Accuracies, Data)) OR TS=(Accuracy, Data)) OR TS=(Data Accuracies)) OR TS=(Data Quality)) OR TS=(Data Qualities)) OR TS=(Qualities, Data)) OR TS=(Quality, Data) **and** Preprint Citation Index **(Exclude – Database)**

#13 ((((((TS=(validity)) OR TS=(reliability)) OR TS=(feasibility)) OR TS=(repeatability)) OR TS=(consistency)) OR TS=(accuracy)) OR TS=(reproducibility) **and** Preprint Citation Index **(Exclude – Database)**

#14 #11 OR #12 OR #13 **and** Preprint Citation Index **(Exclude – Database)**

#15 #14 AND #9 AND #10 AND #5 **and** Preprint Citation Index **(Exclude – Database)**

APPENDIX A

Complete Inclusion/Exclusion Criteria

<u>For a study to be included in the review it must either:</u>

i) report the **concurrent validity** (i.e., simultaneous collection) of outcomes measured by IMUs and compared to a gold standard**,
ii) report the **test-retest reliability** (i.e., between-day, within-day, or between-tester; involving the same measure/device/placement with removal between session) of outcomes measured by IMUs.

<u>Additionally, studies must have included:</u>

iii) measured specific parameters it must either:
  a. gait spatiotemporal: walking speed, cadence, stance time, swing time, step time, stride time, step length, stride length, step frequency and above spatiotemporal variability and symmetry,
  b. static postural balance,
  c. STS, TUGT or 6MWT,
  d. during laboratory settings.
iv) older adults between 60+ years of age, living in community or nurse home, ADL., with no neurological diseases (such as stroke, spinal cord injury, multiple sclerosis, Parkinson's disease, etc.), musculoskeletal diseases (such as hip and knee replacement, severe fracture of lower limbs, etc.), cognitive dysfunction (such as Alzheimer's disease), etc., which seriously affect their mobility function.
v) be published in English

<u>Definitions:</u>

****Inertial sensor*** was defined as a wearable sensor that utilizes any combination of accelerometer, gyroscope, and/or magnetometer signals.

*****Gold standard for variables include***: Commercially available three-dimensional motion capture (i.e., stereophotogrammetry), force plate(s), instrumented mat (e.g., GAITRite), instrumented treadmill, footswitch systems, directly observed or pedometer (only applicable for step count), stop watch (only applicable for TUGT/STS/6MWT duration).

Note: Custom devices or other inertial measurement systems would not qualify as gold standards.

<u>Studies were excluded from the review if they ONLY examined:</u>

i) Event identification (e.g., initial contact, toes off, posture transition, fall…);
ii) activity/movement classification (e.g., frailty, sedentary behavior, fall risk…);
iii) gait events as outcomes (e.g., timing of initial contact);
iv) only assessed upper limb function;
v) daily/weekly physical activity (i.e., counts), energy expenditure;
vi) sensitivity or specificity parameters;
vii) non-human subjects, or

viii) measures derived using supplementary sensors (e.g., global position system, footswitches, etc.).

Supplementary Information 3. The characteristics of included articles.

Author(s), Year [Reference No.]	Participants (sample size, age, height, mass, BMI)	IMUs					Reference system	Measurement	Parameters
		Name(manufacturer)	Composition	No.	Placement	Frequency			
Adamowicz et al.,2020(V) (1)	33 participants<br>17males/16females<br>age: 65-85 years	IMU (Opal v2, APDM, Portland, OR, USA)	NA	6	both feet and wrists, sternum and lower back	128Hz	OMC (Simi Reality Motion Systems, Germany)	STS transition	STS duration (s)
Alqahtani et al.,2020(R) (2)	38participants<br>5males/33female<br>age: 76.4±6.5 years,<br>BMI: 31±10.1kg/m^2	The dual axis accelerometer (ADXL213AE, Analog Devices, Inc., Norwood, MA)	NA	1	back at the level of the iliac crest	50Hz	-	foot together stance; semi-tandem stance; tandem stance	AP/ML:<br>RMS sway(mG)/<br>NPL (normalized path length) sway(mG/s)
Álvarez MN et al.,2023(V) (3)	163participants<br>45Males/118Females<br>age 82.6±6.2 years<br>height: 1.57±0.10m<br>mass:64.3±13.1 kg	NA	NA	1	foot	NA	stopwatch	15-min walking test at a self-selected pace	gait speed (m/s)
Bäcklund et al.,2020(V/R) (4)	*validity analysis:*<br>87 participants<br>37males/50females<br>*reliability analysis:*<br>10 participants<br>4males/6females	IMUs (ADIS16448, Analog Devices, USA)	NA	2	the upper part of the shanks	256Hz	GAITRite® (NJ, USA)	normal preferred speed on the 6.09m(validity analysis)/ 20m (reliability analysis) walkway	*validity analysis:*<br>stride time (s)<br>*reliability analysis:*<br>step width (cm)
Bautmans et al.,2011(R) (5)	41participants<br>21males/20female<br>age: 70.6±6.2 years,<br>height: 164.6±7.7cm,<br>mass: 69.7± 11.4kg	3-D piezoresistive accelerometer (DynaPort MiniMod, McRoberts, The	NA	1	at the sacrum between the spinae ilaca posterior superior.	100Hz	-	walk comfortably on the floor of a 18m straight corridor	gait speed (m/s)<br>step time asymmetry (%)

		Hague)							
Bochicchio et al.,2023(V)(6)	62participants 32males/30females age:66.7±5.9 years; height:1.68±0.09m; mass:73.9±15.4kg; BMI:26.2±4.6kg/m^2	A wearable IMU (Gyko, Microgate, Bolzano, Italy)	a gyroscope and magnetometer	1	the lateral face of the right thigh	500Hz	MoCap (Vicon, UK), force platform (AMTI Inc., USA)	5 consecutive repetitions of the sit-to-stand movement quickly	5 sit-to-stand total time (s), mean concentric velocity (m/s), mean power (MP) (W)
Burton et al., 2018(V/R)(7)	31 participants 11 males/20 females age: 74.2 ± 5.78 years; height:168.9±8.6cm mass: 75.2±14.8kg	Fitbit Flex and Fitbit ChargeHR (Fitbit, Inc.; San Francisco, United States of America)	tri-axial accelerometer	2/2	two Flex to the left wrist; two Fibit ChargeHR to the right wrist.	NA	video count	walk without assistance as fast and safe as permissible for 2 minutes in a hallway	steps count
Byun et al.,2016 (V/R)(8)	82 older adults 47males/35 females, age: 68.67±6.14 years height: 161.23±7.23cm mass: 65.24±9.80 kg	FITMETER1 (FitLifeInc, Suwon, Korea, hereafter FITMETER)	NA	1	the level of the 3rd-4th lumbar vertebrae, which is the approximate center of mass	32Hz	GAITRite® (CIR Systems Inc., Havertown, PA)	walk 20m straight at preferred, comfortable speed	Cadence(steps/min) Velocity(m/s) Step time(s) step length(cm) Step time variability (%) Step time asymmetry (%)
Byun et al.,2019(V)(9)	197participants 90males/107females age:73.6±4.2 years mass: 61.7±9.2kg	FITMETER1 (FitLife Inc., Suwon, Korea,)	a tri-axial accelerometer and gyroscope	1	the level of the third–fourth lumbar vertebrae	250Hz	GAITRite™	walk 14m straight at comfortable speed	walk speed(cm/s)
Cerrito et al., 2016(V/R)(10)	16 participants 16males/0females age: 73.5 ±10.4 years height:171.9 ±5.9 cm mass: 81.4 ±11.9 kg	the mobile operating system (OS) Android (Google Inc., Mountain View, CA, USA)	a triaxial accelerometer and a rotation vector sensor	2	the lower back (spinous process of the third lumbar vertebra [L3]); the sternum	59± 13 Hz	two separate force plates (Type 9286BA, Kistler, Winterthur, Switzerland)	sit-to-stand movement as fast as possible	Peak force(N/kg) peak power(W/kg) total duration(s)
Chan et al.,2016(V)(	32 participants 11males/21 females;	an android-based	NA	1	chest	NA	A force sensor (YZC-516;	STS (complete five repetitions of the	duration (s)

11)	age:70.7 ± 6.5 years	smartphone<br>(Galaxy Note II;<br>Samsung<br>Electronics Co.<br>Ltd, Suwon,<br>Korea)					Guangzhou,<br>China)	sit-to-stand<br>manoeuvre)<br>TUGT (self-paced<br>comfortable<br>walking speed)	
Cole et<br>al.,2014(V)(<br>12)	12 participants<br>6 males/ 6 females<br>age: 71.2±4.0 years<br>height: 169.2±11.8cm<br>mass: 73.9±14.1 kg<br>BMI: 25.6±2.5 kg/m^2	An<br>InertiaCube3<br>tri-axial IMU<br>(InterSense Inc.,<br>Bedford MA,<br>USA)	NA	1	the 12th thoracic<br>vertebra	100Hz	OMC (Vicon<br>Nexus;<br>Oxford, UK).	walked barefoot at a<br>self-selected and<br>preferred pace along<br>a firm surface with<br>different conditions	RMS accelerations<br>(AP/ML/VT)
Contreras C<br>et<br>al.,2024(V/<br>R)(13)	20 participants<br>12males/8females<br>age:74.2±4.2 years<br>height:1.71±0.13m<br>weight:75.9±16.5kg<br>BMI:25.8±4.4kg/m^2	Smartphones<br>(iPhone SE,<br>Apple Inc.,<br>Cupertino, CA,<br>USA)	NA	2	right and left<br>anterolateral thighs	100Hz	3D optical<br>motion capture<br>system<br>(VICON,Oxfo<br>rd, UK)	treadmill walk at<br>self-selected pace	Step length(m)<br>Stride length(m)<br>Cadence(steps/min)<br>walking speed(m/s)
De Groote<br>et<br>al.,2021(V/<br>R)(14)	97 participants<br>42males/55females	Samsung<br>Galaxy S7<br>smartphone<br>(Samsung,<br>Seoul, Korea)	Linear and<br>rotational<br>accelerations	1	the level of the<br>second sacral vertebra	500 Hz	BTrackS<br>Balance Plate<br>(California,<br>USA)	bipodal stance<br>semi-tandem stance	RMS Acc<br>Mean Acc
Digo et<br>al.,2023(V)(<br>15)	16 participants<br>8 males/8 females)<br>age: 68.3±4.4 years<br>height: 1.6± 0.1 m<br>mass: 68.7 ±13.4 kg,<br>BMI: 25.1 ± 3.0 kg/m^2	MTx IMUs<br>(Xsens, The<br>Netherlands)	a tri-axial<br>accelerometer, a<br>tri-axial<br>gyroscope, and a<br>tri-axial<br>magnetometer.	5	trunk-IMU: T12-L1<br>level;<br>shank-IMU: proximal<br>anterior part of the<br>tibia;<br>ankle-IMU: insertion<br>of Achilles tendon<br>into calcaneum	50Hz	optoelectronic<br>system<br>(OptiTrack,<br>USA)	walk 6.5m at slow,<br>normal, and fast<br>speed	stride time (s)<br>step time (s)<br>stance time (s)<br>swing time (s)
Donath et<br>al.,2016(V/<br>R)(16)	24participants<br>13males/11females<br>age: 75.3± 6.7years	The RehaGait1<br>system<br>(Hasomed	a 3-axial<br>accelerometer,<br>gyroscope and	2	the lateral aspect of<br>the shoe	500Hz	Treadmill<br>(Zebris<br>FDM-T, Zebris	walk 5min at three<br>walking speeds and<br>two slopes	walking speed (m/s)<br>stride length (m)<br>cadence (steps/min)

	height: 1.65± 0.12m<br>mass: 77.0 ±12.6 kg<br>BMI:28.0±7.3 kg/m^2	GmbH,<br>Magdeburg,<br>Germany)	magnetometer.				medical<br>GmbH, Isny,<br>Germany)		stride time (s)
Ensink C et al.,2023(V)(17)	20 participants<br>10 Males/10Females<br>age 59±12 years<br>height: 174±7.2 cm<br>mass:75±8.0 kg	IMUs (MTw Awinda, Xsens, Enschede)	NA	4	dorsal side of both feet, sternum, and lower back (L4/5)		3D optical motion capture system (VICON,Oxford, United Kingdom)	Treadmill walk: 2 min at a self-paced, comfortable speed with regular and irregular condition;<br>Overground walk: approximately 5 m at a comfortable speed	stride time (s)<br>stride length (m)<br>stride velocity (m/s)
Ferrari L et al.,2024(V)(18)	65 participants,<br>33 male/32female<br>age:66.7±5.83<br>height:1.68±9.58m<br>mass: 73.1±15.3kg<br>BMI:25.90±4.70kg/m^2	IMU sensor (GYKO, Microgate, Bolzano, Italy)	NA	1	lumbar spine (i.e., L5 level)	500Hz	single force plate (1000 Hz, AMTI Inc., Watertown, MA, USA)	two (EO and EC conditions) 30 s standing balance tests with feet in a semi-tandem position	AP/ML:<br>total length<br>95% CI ellipse area
Foster et al.,2022(V)(19)	40 participants<br>17 males/23 females)<br>age: 67.9±6.5 years	The Garmin Vivofit®4 (Garmin Ltd.©, Kansas, MO);<br>The activPAL3™ (PAL Technologies Ltd.©, Glasgow, UK)	triaxial accelerometer in each IMU	NA	Garmin: nondominant wrist.<br>ActivPAL3: anterior thigh of the nondominant leg, midway between the knee and the hip	20Hz	Kaiser Baas X90 action camera (Kaiser Baas, Melbourne, Australia)	5-min increments of walking at four speeds in randomized order (0.44, 0.89, 1.33, and 1.67 m/s) at a 0% grade.	steps count
Fudickar et al.,2020(V)(20)	91 participants<br>44males/47females<br>age:77.87±3.59 years	Inertial sensor (Bosch)	a triaxial accelerometer, gyroscope, magnetometer, and a one-dimensional	1	between the L3 and L5 lumbar vertebral body	100Hz	stopwatch	standup- and walk test;<br>5 times chair-rise test	time (s)

			barometer						
Greene et al., 2022(R)(21)	168 participants<br>51males/117females<br>age:75.0 ± 7.2years<br>height:163.1 ±9.0cm<br>mass:73.2 ± 12.7kg	IMU (Shimmer2R, Shimmer Research, Dublin, Ireland)	a tri-axial accelerometer and a tri-axial gyroscope.	2	*thigh sensor:*the lateral aspect of the thigh;<br>*torso sensor:* the sternum	102.4Hz	-	fully stand up and sit back down five times quickly	Individual STS duration (s)<br>CV of single STS duration (%)<br>Total STS duration (s)<br>Stand-up time (s)<br>CV of individual standup time (%)<br>Sit time (s)<br>CV of Sit time (%)
Grimpampi et al., 2015 (R)(22)	29 participants<br>5males/24females<br>age: 84 ± 5 years<br>height:1.59±0.08m<br>mass:75±15kg<br>BMI:30±6kg/m^2	IMU (FreeSense, Sensorize, Rome)	NA	1	lower lumbar spine	200Hz	-	6 min walk distance as fast as they could	Unbiased autocorrelation coefficients (AP/ML/VT)
Hamacher et al.,2014 (R)(23)	19 participants<br>5 male/14female<br>age: 71±4 years	wireless inertial motion tracker (MTw sensors, Xsens Technologies B.V., Netherlands)	NA	1	forefeet	NA	-	walked continuously five times up and down a level hallway (distance 25 m) at their preferred walking speed	walking speed (m/s)<br>stride length (m)<br>stance time (s)<br>swing time (s)<br>their corresponding measures of variability (%)
Hartmann et al.,2009① (V)(24)	23 participants<br>7males/16females<br>age:77.2±4.7 years<br>height:1.65±0.09m<br>weight:66.5±10.9kg<br>BMI:24.2±2.6kg/m^2	The DynaPort ®MiniMod tri-axial accelerometer (McRoberts BV, The Hague, The Netherlands)	NA	1	level of the second sacral vertebrae	100Hz	GAITRite® system (CIR Systems Inc., Havertown, USA)	walk over 13m walkway at preferred, slow and fast self-selected speed	walking speed (m/s)<br>cadence (steps/min)<br>step duration (s)<br>variability of step duration (%)<br>step length (m)<br>variability of step length (%)
Hartmann et al.,2009② (R)(25)	23participants<br>8 males/ 15 females<br>age: 73.4±4.3 years,<br>height 1.67±0.09 m,<br>mass: 70.8±13.1 kg	DynaPort1MiniMod (McRoberts BV, The Hague, The Netherlands)	tri-axial accelerometer	1	level of the second sacral vertebrae	100Hz	-	walk over 24 m in a gym at preferred speed with different surface and cognitive task.	walking speed (m/s)<br>cadence (steps/min)<br>step duration (s)<br>variability of step duration (%)<br>step length (m)

	BMI 25.4 ±4.2 kg/m^2								variability of step length (%)
Hellmers et al., 2018(V)(26)	147participants<br>60 males/87 females<br>age: 75.22 ±3.83 years<br>height :167.43 ±9.50 m<br>mass:76.01 ±13.94 kg	sensor belt	a triaxial accelerometer, gyroscope, magnetometer, and a barometer	1	between the L3 and L5 lumbar vertebral	100 Hz	stopwatch and automated measurements	TUGT test	duration
Kobsar et al.2014(V)(27)	41 participants<br>16 male/25 female<br>age: 75.68 ± 5.39 years	a triaxial accelerometer (ADXL 330, Analog Device, Inc.,Norwood, MA)	a triaxial accelerometer	1	level of the 3rd lumbar vertebra	100Hz	footswitch	a ten-minute walk at a normal, self-selected pace around a 200 m indoor track	mean step time (ms)<br>mean stride time(ms)<br>step time SD (ms)<br>stride time SD (ms)
Koose et al.,2014(R)(28)	23participants<br>7males/16female<br>age: 65±5.5 years	the iPod Touch G4 (iOS 6; Apple Inc.)	a built-in triaxial acceleration sensor	1	the level of lumbar segment L3	88-92Hz	-	parallel stance and semi-tandem stance with eyes open/closed/dual task	RMS AP/ML<br>median power frequency AP/ML<br>Sway area
Kuntapun et al., 2020(V/R)(29)	12 participants<br>3 males/9females<br>age: 75.6± 5.6 years<br>height: 1.60±0.09m<br>mass: 58.0±6.6kg	Android smartphones (Samsung J7+Android 7.1.1)	tri-axial accelerometer	2	level of the third lumbar vertebrae; vertically inside a shoulder bag (13 × 20 cm) and rested on the right hip	50Hz	OMC (Motion Analysis Corp., Santa Rosa, CA)	walk 10m at preferred speed	gait velocity (m/s)<br>step time (ms)<br>step length (cm)<br>cadence (steps/min)
Maganja et al.,2020 (R)(30)	36 participants<br>17males/19females<br>age 71.4±4.7 years<br>mass:82.0±16.8kg<br>BMI:29.4±5.9 kg/m^2	Misfit Shine (Burlingame, USA); Fitbit One, Fitbit Charge (San Francisco, USA); Jawbone UP2 (San Francisco, USA); Garmin vívofit	each sensor is equipped with a three-axis accelerometer	5	Misfit Shine: hip;<br>Fitbit One: hip;<br>Fitbit Charge: wrist;<br>Jawbone UP2: wrist<br>Garmin vívofit 2: wrist	NA	-	walk 100 steps at preferred speed	steps count

		2 (Olathe Kansan, USA)							
Maggio et al.,2016(V)(31)	172 Participants<br>69males/103females<br>age: 80.7±4.8 years	the inertial triaxial sensor (Free4Act1, LorAn-Engineering, Bologna, Italy)	a tri-axial accelerometer	1	the L4-L5 inter-vertebral space	NA	stopwatch	walk 4m at usual pace	gait speed
Magistro et al.,2018 (V/R)(32)	20 older adults<br>10 males/10 females<br>age: 75 ± 7 years;<br>BMI: 27.4 ± 5.4 kg/m^2	ADAMO Care Watch	a 3-axis accelerometer (ADX346, USA)	2	wrist; arm	50Hz	observed directly	walk 30m at normal/slow/fast speed;<br>timed up and go at comfortable speed	steps count
Mancini et al.,2012(R)(33)	17 participants<br>age: 67.9 ± 6.1 years	MTX Xsens sensor (49A33G15, Xsens, Enschede, NL)	3D accelerometers	1	the posterior trunk at the level of L5	50Hz	-	static stance for 30s	RMS acceleration (m/s^2)<br>path length (m/s^2)<br>jerk (m^2/s^5)<br>distance (m/s^2)<br>range (m/s^2)<br>mean velocity (mm/s)<br>mean frequency (Hz)<br>area (m^2/s^5)
Marques et al.,2021(V/R)(34)	40 participants<br>20males/20females;<br>age:78.9±8.6 years;<br>height:1.57±0.1m<br>mass:65.4±11.6 kg	Xiaomi Mi A1	a triaxial accelerometer (model Bosch BMI120)	1	inside a waistband	200Hz	digital video camera (Canon LEGRIA HF R46, Tokyo, Japan)	sit to stand test	stand-up time (s)<br>total time (s)
Matikainen-Tervola E et al.,2024(V)(35)	26 participants<br>9 males/17females<br>mean age76 years<br>mean height: 165 cm	IMUs (NGIMU, x-ion, UK)"	tri-axis accelerometer (± 16 g), tri-axis gyroscope (rotations ± 2000 °/s), and tri-axis magnetometer (magnetic field ±	3	midway along both shanks laterally and on the lower back at the level of L3-L4	200Hz	3D optical motion capture system (VICON, Oxford, UK)	treadmill walk 3min at predetermined self-selected speed	step time(s)<br>stride time(s)<br>swing time(s)<br>stance time(s)<br>step length(cm)<br>walking speed(m/s)

			1300 μ T)						
Micó-Amigo et al.,2016(V)(36)	20 participants<br>11 males/9 females<br>age: 73.7 ± 7.9 years<br>height: 173.3 ± 8.2 cm,<br>mass: 77.7 ± 13.1 kg	BFS (DynaPort Hybrid, McRoberts)	a triaxial accelerometer and a triaxial gyroscope	1/2	*1st system:* the level of the lowest lumbar vertebra (L5);<br>*2nd system:* the lateral sides of both heels	100Hz	stopwatch	walk 5m at preferred speed	step duration (s)
Motti Ader et al.,2021(R)(37)	98participants<br>30 males/ 68 females<br>age: 70.6±6.2 years,<br>height: 167.5±9.0m,<br>mass: 74.5± 14.9kg<br>BMI 26.6 ±5.0 kg/m^2	inertial sensors (SHIMMER™, Shimmer Research, Dublin, Ireland)	a tri-axial accelerometer and a tri-axial gyroscope	2	mid-point of the anterior shank of each leg	102.4 Hz	-	walk 30m at a comfortable self-selected speed	stance/stride/swing/step time (s)<br>stride length (cm)<br>stride velocity (cm/s)<br>above parameters corresponding variability (%) and asymmetry (%)
Orange et al.,2020(V)(38)	32 participants<br>11males/21 females;<br>age: 70.7 ± 6.5 years	an android-based smartphone (Galaxy Note II; Samsung Electronics Co. Ltd, Suwon, Korea)	NA	1	chest	NA	A force sensor (YZC-516, Guangzhou, China)	sit-to-stand manoeu(V/R)e as quickly as possible.	duration (m)
Ozinga et al.,2014(V)(39)	12 participants<br>5 males/7 females<br>age: 68.3 ± 6.9 years	iPad	an accelerometer and gyroscope	1	waist	100Hz	OMC (Motion Analysis Corporation Eagle System, CA)	double-leg/tandem stance for 60s	peak-to-peak<br>normalized path length<br>RMS distance<br>95% volume<br>power
Pedrero-Sánchez et al.,2023(R)(40)	65 participants<br>age:68.55±7.18 years<br>height: 1.62±0.09m<br>mass: 76.04±12.78kg	smartphone (Xiaomi Redmi 4 x Model MAG138)	a 3-axis gyroscope, 3-axis accelerometer, and Digital Motion Processor ™ (TDK-ICM-20689; Japan)	1	below the iliac crest point near the centre of mass	100Hz	-	30s standing	AP/ML displacement (mm)

Peller et al.,2022 (V)(41)	93particiapants<br>38 male/55 females<br>age:73.03±6.09 years<br>height:66.133±3.89in<br>mass: 60.89±36.22lb	NIH Toolbox® app	NA	1	anteriorly between the iliac crests	NA	-	50s double leg/ tandem standing	uncorrected average Theta score
Phillips et al.,2015(V)(42)	50 participants<br>5males/45females<br>age:84.2±7.1 years	Fitbit® Tracker; GT1M"	biaxial accelerometer	4	*Fitbit*: waist<br>*GT1M*: ankle, waist, and wrist	NA	observed count	NA	steps count
Pooranawatthanakul et al.,2023 (V/R)(43)	20 participants<br>3 male/17female<br>age:70.855±4.09<br>height:1.55±0.06m<br>mass: 57.05±7.72kg<br>BMI:23.94±3.22kg/m^2	Samsung A5 (2017) smartphone (Samsung Electronics Co., Ltd., Suwon, South Korea)	NA	1	midpoint of the sternum	50Hz	OMC (Raptor E, Motion Analysis Corporation, Santa Rosa, CA)"	double/single leg stance	RMS acceleration
Rantalainen et al.,2019 (V/R) (44)	27 participants<br>10 males/ 17 females<br>age: 74.4±4.3 years<br>height: 165.9±9.9cm<br>mass: 74.5±12.0 kg	inertial measurement unit (NGIMU, x-io Technologies, Bristol, UK)	3-dimensional accelerations	1	lateral malleolus	400Hz	OMC (Vicon T40, Oxford, UK)	3 min self-paced walk up and back a 14 m track	stance/swing/stride duration (ms)<br>stride length (m)<br>stride velocity (m/s)<br>above parameters corresponding standard deviation
Rantalainen et al.,2020 (V/R) (45)	27participants<br>10males/17females<br>age 74.4±4.3 years<br>height:165.9±9.9cm<br>mass:74.5±12.0kg	inertial measurement unit (NGIMU, x-io Technologies, Bristol, UK)	3-dimensional accelerations	2	lateral malleolus; the waist just below the iliac crest in the mid-line of the back around the L4 level	400Hz	OMC (Vicon T40, Oxford, UK)	3 min self-paced walk up and back a 14 m track	mean stride duration (ms)<br>stride duration standard deviation(ms)<br>stride duration variability (%)
Regterschot et al.,2014 (R)(46)	31 participants<br>10 males/ 21 females<br>age: 82.5 ±4.9 years<br>height :1.66 ±0.10 m<br>mass:79.9 ±14.3 kg	hybrid motion sensor (p-Node, Philips)	an accelerometer, a gyroscope and a magnetometer	1	the right side of the hip	50Hz	-	normal/fast speed sit to stand;<br>timed up and go	normal STS/Fast STS duration (s)<br>maximal acceleration (m/s^2)<br>maximal jerk (m/s^3)<br>maximal velocity (m/s)<br>peak power (W)

									five times STS duration (s)
Regterschot et al.,2016(V)(47)	27 participants<br>7males/20females<br>age:81.7±5.6 years<br>height:1.63±0.09m<br>mass:75.7±13.3kg	body-fixed motion sensors (p-Node, Philips)	a 3D accelerometer, a 3D gyroscope and a 3D magnetometer	2	right side of the hip; chest	50Hz	force plates (Bertec)	five chair rise movements at a normal speed from a standard chair	STS duration (s)<br>maximal acceleration (m/s^2)<br>maximal jerk (m/s^3)<br>maximal velocity (m/s)<br>Peak power (W)
Rogan et al.,2017 (V)(48)	21 participants<br>age: 89.8 years<br>height:1.61 ± 0.2 m<br>mass: 63.9 ± 15.0 kg	RehaWatch®	three accelerometers and three gyroscopes	2	lateral malleolus of both the left and right leg	500Hz	GAITRite®	walk at preferred/fast speed with single/dual task	Step duration time left feet (s)<br>Step duration time right feet (s)<br>Velocity (m/s)<br>Cadence (/m)<br>Step length left (cm)<br>Step length right (cm)"
Rüdiger et al.,2019 (V)(49)	32 participants<br>14males/18 females,<br>age: 74.8±5.9 years	The Polar M400©	a triaxial accelerometer	1	non-dominant arm	NA	directly observed	walk 200m at normal speed	steps count
Rudisch et al.,2021 (V)(50)	12 participants<br>6males/6females<br>age:72.1±7.9 years<br>height:169.8±8.3cm<br>mass:74.0±12.3kg	GaitUp (GaitUp S.A., Switzerland)	NA	2	dorsum of the left and right foot	128Hz	GAITRite; Optogait; Zebris	walk 14.7m at preferred speed	gait cycle time (s)<br>cadence (steps/min)<br>stride length (m)<br>gait speed(m/s)
Saunders et al.,2015 (R)(51)	20 participants<br>8 males/12 females<br>age: 81±4 years	YEI 3-Space Sensor Wireless accelerometer (Yost Engineering Inc., Portsmouth, OH)	accelerometer	1	lower backs at the L3 vertebral level	250 Hz	-	30s static stance with open/closed eyes, floor/mat	RMS acceleration
Smith et al.,	12 participants	SHIMMER	a triaxial	2	mid-point of the	102.4 Hz	-	timed up and go at	TUGT duration (s)

2016 (R)(52)	2 males/10 females age:74.17±3.88 years	kinematic sensors (Kinesis QTUGTM)	accelerometer and an addon triaxial gyroscope		shank			fast speed	steps count
Song et al.,2022 (V)(53)	20participants 6males/14females age:72.4±1.57 years; height:1.67±0.01m; mass:68.44±2.79kg; BMI:24.57±0.87kg/m^2	a smartphone (iPhone 6s; Apple Inc., Cupertino, CA)	3D accelerometer	1	1/3 of the distance from the anterior superior iliac spine to the superior border of the patella	NA	force plate (AMTI, Watertown, MA); OMC (Miqus; Qualisys, Sweden)	Sit to stand at normal/ fast speed	duration (s)
Walgaard et al.,2016 (V)(54)	27 participants 14males/13females age:74.7±8.5 years height:172.2±8.2cm mass:76.8±13.2kg	IS (DynaPorts Hybrid, McRoberts)	3 accelerometers and 3 gyroscopes	1	the level of the fourth lumbar spinous process	100Hz	OMC (Optotrak Certuss, Northern Digital Inc.)	sit to walk	acceleration (m/s^2) velocity(m/s) displacement (m) rotation (degree)
Werner et al.,2023 (R)(55)	28partcicipants 6males/22females age:75.6±5.7 years BMI: 23.5±2.7 kg/m^2	Apple Health app (Cupertino, CA, USA)	NA	1	the right front pocket of their pants	NA	-	walk continuously 6 min a flat and straight 20-m walkway	gait speed(m/s) step length(cm)
Zhang et al., 2014(56)	41 participants 13males/28females age:81.9 ± 5.5years height:1.65 ± 0.09m mass:78.5 ± 14kg	a matchbox-sized hybrid motion sensor	a 3D accelerometer	1	a necklace, which was left hanging unrestricted in front of the chest	50Hz	-	sit to stand at normal/fast speed	duration (s) max vert Acc peak power max jerk

(V) represents validity analysis; (R) represents reliability analysis; OMC optimal motion capture system.

1. Adamowicz L, Karahanoglu FI, Cicalo C, Zhang H, Demanuele C, Santamaria M, et al. Assessment of Sit-to-Stand Transfers during Daily Life Using an Accelerometer on the Lower Back. SENSORS. 2020;20(22).

2. Alqahtani BA, Sparto PJ, Whitney SL, Greenspan SL, Perera S, Brach JS. Psychometric properties of instrumented postural sway measures recorded in community settings in independent living older adults. BMC geriatrics. 2020;20(1):82.

3. Álvarez MN, Ruiz ARJ, Neira GG, Huertas-Hoyas E, Cerda MTE, Delgado LP, et al. Assessing falls in the elderly population using

G-STRIDE foot-mounted inertial sensor. Scientific reports. 2023;13(1):9208.

4. Bäcklund T, Öhberg F, Johansson G, Grip H, Sundström N. Novel, clinically applicable method to measure step-width during the swing phase of gait. Physiological measurement. 2020;41(6):065005.

5. Bautmans I, Jansen B, Van Keymolen B, Mets T. Reliability and clinical correlates of 3D-accelerometry based gait analysis outcomes according to age and fall-risk. Gait & posture. 2011;33(3):366-72.

6. Bochicchio G, Ferrari L, Bottari A, Lucertini F, Scarton A, Pogliaghi S. Temporal, Kinematic and Kinetic Variables Derived from a Wearable 3D Inertial Sensor to Estimate Muscle Power during the 5 Sit to Stand Test in Older Individuals: A Validation Study. Sensors. 2023;23(10).

7. Burton E, Hill KD, Lautenschlager NT, Thøgersen-Ntoumani C, Lewin G, Boyle E, et al. Reliability and validity of two fitness tracker devices in the laboratory and home environment for older community-dwelling people. BMC geriatrics. 2018;18(1):103.

8. Byun S, Han JW, Kim TH, Kim KW. Test-Retest Reliability and Concurrent Validity of a Single Tri-Axial Accelerometer-Based Gait Analysis in Older Adults with Normal Cognition. PLoS ONE. 2016;11(7):e0158956.

9. Byun S, Lee HJ, Han JW, Kim JS, Choi E, Kim KW. Walking-speed estimation using a single inertial measurement unit for the older adults. PLoS ONE. 2019;14(12):e0227075.

10. Cerrito A, Bichsel L, Radlinger L, Schmid S. Reliability and validity of a smartphone-based application for the quantification of the sit-to-stand movement in healthy seniors. Gait & posture. 2015;41(2):409-13.

11. Chan MHM, Keung DTF, Lui SYT, Cheung RTH. A validation study of a smartphone application for functional mobility assessment of the elderly. Hong kong physiotherapy journal. 2016;35:1-4.

12. Cole MH, Van Den Hoorn W, Kavanagh JK, Morrison S, Hodges PW, Smeathers JE, et al. Concurrent validity of accelerations measured using a tri-axial inertial measurement unit while walking on firm, compliant and uneven surfaces. PLoS ONE. 2014;9(5).

13. Contreras C, Stanley EC, Deschamps-Prescott C, Burnap S, Hopkins M, Browning B, et al. Evaluation of Smartphone Technology on Spatiotemporal Gait in Older and Diseased Adult Populations. Sensors (Basel, Switzerland). 2024;24(17).

14. De Groote F, Vandevyvere S, Vanhevel F, Orban de Xivry J-J. Validation of a smartphone embedded inertial measurement unit for

measuring postural stability in older adults. Gait & posture. 2021;84:17-23.

15. Digo E, Panero E, Agostini V, Gastaldi L. Comparison of IMU set-ups for the estimation of gait spatio-temporal parameters in an elderly population. Proceedings of the Institution of Mechanical Engineers Part H, Journal of engineering in medicine. 2023;237(1):61-73.

16. Donath L, Faude O, Lichtenstein E, Pagenstert G, Nüesch C, Mündermann A. Mobile inertial sensor based gait analysis: Validity and reliability of spatiotemporal gait characteristics in healthy seniors. Gait Posture. 2016;49:371-4.

17. Ensink C, Smulders K, Warnar J, Keijsers N. Validation of an algorithm to assess regular and irregular gait using inertial sensors in healthy and stroke individuals. PeerJ. 2023;11:e16641.

18. Ferrari L, Bochicchio G, Bottari A, Scarton A, Lucertini F, Pogliaghi S. Construct Validity of a Wearable Inertial Measurement Unit (IMU) in Measuring Postural Sway and the Effect of Visual Deprivation in Healthy Older Adults. Biosensors. 2024;14(11).

19. Foster JI, Williams KL, Timmer BHB, Brauer SG. Concurrent Validity of the Garmin Vivofit (R) 4 to Accurately Record Step Count in Older Adults in Challenging Environments. JOURNAL OF AGING AND PHYSICAL ACTIVITY. 2022;30(5):833-41.

20. Fudickar S, Hellmers S, Lau S, Diekmann R, Bauer JM, Hein A. Measurement System for Unsupervised Standardized Assessment of Timed "Up & Go" and Five Times Sit to Stand Test in the Community-A Validity Study. Sensors (Basel, Switzerland). 2020;20(10).

21. Greene BR, Doheny EP, McManus K, Caulfield B. Estimating balance, cognitive function, and falls risk using wearable sensors and the sit-to-stand test. Wearable Technol. 2022;3.

22. Grimpampi E, Oesen S, Halper B, Hofmann M, Wessner B, Mazza C. Reliability of gait variability assessment in older individuals during a six-minute walk test. JOURNAL OF BIOMECHANICS. 2015;48(15):4185-9.

23. Hamacher D, Hamacher D, Taylor WR, Singh NB, Schega L. Towards clinical application: repetitive sensor position re-calibration for improved reliability of gait parameters. Gait & posture. 2014;39(4):1146-8.

24. Hartmann A, Luzi S, Murer K, de Bie RA, de Bruin ED. Concurrent validity of a trunk tri-axial accelerometer system for gait analysis in older adults. Gait & posture. 2009;29(3):444-8.

25. Hartmann A, Murer K, de Bie RA, de Bruin ED. Reproducibility of spatio-temporal gait parameters under different conditions in older adults using a trunk tri-axial accelerometer system. Gait & posture. 2009;30(3):351-5.

26. Hellmers S, Izadpanah B, Dasenbrock L, Diekmann R, Bauer JM, Hein A, et al. Towards an Automated Unsupervised Mobility Assessment for Older People Based on Inertial TUG Measurements. Sensors (Basel, Switzerland). 2018;18(10).

27. Kobsar D, Olson C, Paranjape R, Barden JM. The validity of gait variability and fractal dynamics obtained from a single, body-fixed triaxial accelerometer. Journal of applied biomechanics. 2014;30(2):343-7.

28. Kosse NM, Caljouw S, Vervoort D, Vuillerme N, Lamoth CJ. Validity and Reliability of Gait and Postural Control Analysis Using the Tri-axial Accelerometer of the iPod Touch. Annals of biomedical engineering. 2015;43(8):1935-46.

29. Kuntapun J, Silsupadol P, Kamnardsiri T, Lugade V. Smartphone Monitoring of Gait and Balance During Irregular Surface Walking and Obstacle Crossing. Frontiers in sports and active living. 2020;2:560577.

30. Maganja SA, Clarke DC, Lear SA, Mackey DC. Formative Evaluation of Consumer-Grade Activity Monitors Worn by Older Adults: Test-Retest Reliability and Criterion Validity of Step Counts. JMIR formative research. 2020;4(8):e16537.

31. Maggio M, Ceda GP, Ticinesi A, De Vita F, Gelmini G, Costantino C, et al. Instrumental and Non-Instrumental Evaluation of 4-Meter Walking Speed in Older Individuals. PLoS ONE. 2016;11(4):e0153583.

32. Magistro D, Brustio PR, Ivaldi M, Esliger DW, Zecca M, Rainoldi A, et al. Validation of the ADAMO Care Watch for step counting in older adults. PLoS ONE. 2018;13(2):e0190753.

33. Mancini M, Salarian A, Carlson-Kuhta P, Zampieri C, King L, Chiari L, et al. ISway: A sensitive, valid and reliable measure of postural control. Journal of NeuroEngineering and Rehabilitation. 2012;9(1).

34. Marques DL, Neiva HP, Pires IM, Zdravevski E, Mihajlov M, Garcia NM, et al. An Experimental Study on the Validity and Reliability of a Smartphone Application to Acquire Temporal Variables during the Single Sit-to-Stand Test with Older Adults. SENSORS. 2021;21(6).

35. Matikainen-Tervola E, Cronin N, Aartolahti E, Sihvonen S, Sansgiri S, Finni T, et al. Validity of IMU sensors for assessing features of walking in laboratory and outdoor environments among older adults. Gait & posture. 2024;114:277-83.

36. Micó-Amigo ME, Kingma I, Ainsworth E, Walgaard S, Niessen M, Van Lummel RC, et al. A novel accelerometry-based algorithm for the detection of step durations over short episodes of gait in healthy elderly. Journal of NeuroEngineering and Rehabilitation. 2016;13(1).

37. Motti Ader LG, Greene BR, McManus K, Caulfield B. Reliability of inertial sensor based spatiotemporal gait parameters for short walking

bouts in community dwelling older adults. GAIT & POSTURE. 2021;85:1-6.

38. Orange ST, Metcalfe JW, Liefeith A, Jordan AR. Validity of various portable devices to measure sit-to-stand velocity and power in older adults. Gait & posture. 2020;76:409-14.

39. Ozinga SJ, Alberts JL. Quantification of postural stability in older adults using mobile technology. EXPERIMENTAL BRAIN RESEARCH. 2014;232(12):3861-72.

40. Pedrero-Sánchez JF, De-Rosario-Martínez H, Medina-Ripoll E, Garrido-Jaén D, Serra-Añó P, Mollà-Casanova S, et al. The Reliability and Accuracy of a Fall Risk Assessment Procedure Using Mobile Smartphone Sensors Compared with a Physiological Profile Assessment. Sensors (Basel, Switzerland). 2023;23(14).

41. Peller A, Garib R, Garbe E, Komforti D, Joffe C, Magras A, et al. Validity and reliability of the NIH Toolbox R Standing Balance Test As compared to the Biodex Balance System SD. Physiotherapy theory and practice. 2023;39(4):827-33.

42. Phillips LJ, Petroski GF, Markis NE. A comparison of accelerometer accuracy in older adults. Res Gerontol Nurs. 2015;8(5):213-9.

43. Pooranawatthanakul K, Siriphorn A. Testing the validity and reliability of a new android application-based accelerometer balance assessment tool for community-dwelling older adults. Gait & posture. 2023;104:103-8.

44. Rantalainen T, Pirkola H, Karavirta L, Rantanen T, Linnamo V. Reliability and concurrent validity of spatiotemporal stride characteristics measured with an ankle-worn sensor among older individuals. Gait & posture. 2019;74:33-9.

45. Rantalainen T, Karavirta L, Pirkola H, Rantanen T, Linnamo V. Gait Variability Using Waist- and Ankle-Worn Inertial Measurement Units in Healthy Older Adults. Sensors (Basel, Switzerland). 2020;20(10).

46. Regterschot GRH, Zhang W, Baldus H, Stevens M, Zijlstra W. Test-retest reliability of sensor-based sit-to-stand measures in young and older adults. Gait & posture. 2014;40(1):220-4.

47. Regterschot GRH, Zhang W, Baldus H, Stevens M, Zijlstra W. Accuracy and concurrent validity of a sensor-based analysis of sit-to-stand movements in older adults. Gait Posture. 2016;45:198-203.

48. Rogan S, de Bie R, de Bruin ED. Sensor-based foot-mounted wearable system and pressure sensitive gait analysis Agreement in frail elderly people in long-term care. ZEITSCHRIFT FUR GERONTOLOGIE UND GERIATRIE. 2017;50(6):488-97.

49. Ruediger S, Stuckenschneider T, Abeln V, Askew CD, Wollseiffen P, Schneider S, et al. Validation of a widely used heart rate monitor to track steps in older adults. JOURNAL OF SPORTS MEDICINE AND PHYSICAL FITNESS. 2019;59(10):1622-7.

50. Rudisch J, Joellenbeck T, Vogt L, Cordes T, Klotzbier TJ, Vogel O, et al. Agreement and consistency of five different clinical gait analysis systems in the assessment of spatiotemporal gait parameters. GAIT & POSTURE. 2021;85:55-64.

51. Saunders NW, Koutakis P, Kloos AD, Kegelmeyer DA, Dicke JD, Devor ST. Reliability and validity of a wireless accelerometer for the assessment of postural sway. Journal of applied biomechanics. 2015;31(3):159-63.

52. Smith E, Walsh L, Doyle J, Greene B, Blake C. The reliability of the quantitative timed up and go test (QTUG) measured over five consecutive days under single and dual-task conditions in community dwelling older adults. Gait & posture. 2016;43:239-44.

53. Song Y, Begum M, Arthanat S, LaRoche DP. Validation of Smartphone Accelerometry for the Evaluation of Sit-To-Stand Performance and Lower-Extremity Function in Older Adults. Journal of aging and physical activity. 2022;30(1):3-11.

54. Walgaard S, Faber GS, van Lummel RC, van Dieen JH, Kingma I. The validity of assessing temporal events, sub-phases and trunk kinematics of the sit-to-walk movement in older adults using a single inertial sensor. JOURNAL OF BIOMECHANICS. 2016;49(9):1933-7.

55. Werner C, Hezel N, Dongus F, Spielmann J, Mayer J, Becker C, et al. Validity and reliability of the Apple Health app on iPhone for measuring gait parameters in children, adults, and seniors. Scientific reports. 2023;13(1):5350.

56. Zhang W, Regterschot GRH, Schaabova H, Baldus H, Zijlstra W. Test-Retest Reliability of a Pendant-Worn Sensor Device in Measuring Chair Rise Performance in Older Persons. SENSORS. 2014;14(5):8705-17.

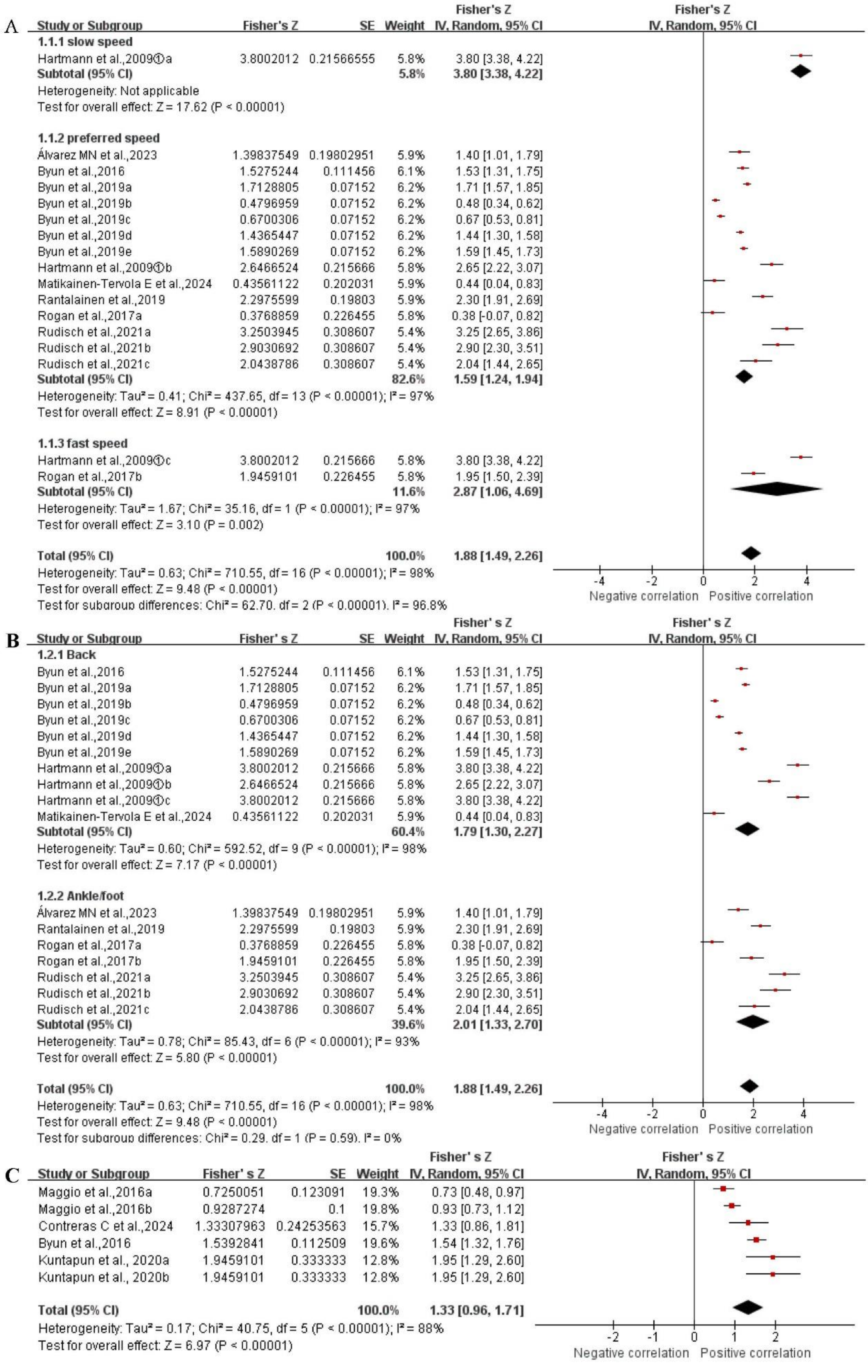
A
Study or Subgroup
Fisher's Z
SE
Weight
Fisher's Z IV, Random, 95% CI
Fisher's Z IV, Random, 95% CI
1.1.1 slow speed
Hartmann et al.,2009①a 3.8002012 0.21566555 5.8% 3.80 [3.38, 4.22]
Subtotal (95% CI) 5.8% 3.80 [3.38, 4.22]
Heterogeneity: Not applicable
Test for overall effect: Z = 17.62 (P < 0.00001)
1.1.2 preferred speed
Álvarez MN et al.,2023 1.39837549 0.19802951 5.9% 1.40 [1.01, 1.79]
Byun et al.,2016 1.5275244 0.111456 6.1% 1.53 [1.31, 1.75]
Byun et al.,2019a 1.7128805 0.07152 6.2% 1.71 [1.57, 1.85]
Byun et al.,2019b 0.4796959 0.07152 6.2% 0.48 [0.34, 0.62]
Byun et al.,2019c 0.6700306 0.07152 6.2% 0.67 [0.53, 0.81]
Byun et al.,2019d 1.4365447 0.07152 6.2% 1.44 [1.30, 1.58]
Byun et al.,2019e 1.5890269 0.07152 6.2% 1.59 [1.45, 1.73]
Hartmann et al.,2009①b 2.6466524 0.215666 5.8% 2.65 [2.22, 3.07]
Matikainen-Tervola E et al.,2024 0.43561122 0.202031 5.9% 0.44 [0.04, 0.83]
Rantalainen et al.,2019 2.2975599 0.19803 5.9% 2.30 [1.91, 2.69]
Rogan et al.,2017a 0.3768859 0.226455 5.8% 0.38 [-0.07, 0.82]
Rudisch et al.,2021a 3.2503945 0.308607 5.4% 3.25 [2.65, 3.86]
Rudisch et al.,2021b 2.9030692 0.308607 5.4% 2.90 [2.30, 3.51]
Rudisch et al.,2021c 2.0438786 0.308607 5.4% 2.04 [1.44, 2.65]
Subtotal (95% CI) 82.6% 1.59 [1.24, 1.94]
Heterogeneity: Tau² = 0.41; Chi² = 437.65, df = 13 (P < 0.00001); I² = 97%
Test for overall effect: Z = 8.91 (P < 0.00001)
1.1.3 fast speed
Hartmann et al.,2009①c 3.8002012 0.215666 5.8% 3.80 [3.38, 4.22]
Rogan et al.,2017b 1.9459101 0.226455 5.8% 1.95 [1.50, 2.39]
Subtotal (95% CI) 11.6% 2.87 [1.06, 4.69]
Heterogeneity: Tau² = 1.67; Chi² = 35.16, df = 1 (P < 0.00001); I² = 97%
Test for overall effect: Z = 3.10 (P = 0.002)
Total (95% CI) 100.0% 1.88 [1.49, 2.26]
Heterogeneity: Tau² = 0.63; Chi² = 710.55, df = 16 (P < 0.00001); I² = 98%
Test for overall effect: Z = 9.48 (P < 0.00001)
Test for subgroup differences: Chi² = 62.70, df = 2 (P < 0.00001), I² = 96.8%
-4 -2 0 2 4
Negative correlation Positive correlation
B
Study or Subgroup
Fisher' s Z
SE
Weight
Fisher' s Z IV, Random, 95% CI
Fisher' s Z IV, Random, 95% CI
1.2.1 Back
Byun et al.,2016 1.5275244 0.111456 6.1% 1.53 [1.31, 1.75]
Byun et al.,2019a 1.7128805 0.07152 6.2% 1.71 [1.57, 1.85]
Byun et al.,2019b 0.4796959 0.07152 6.2% 0.48 [0.34, 0.62]
Byun et al.,2019c 0.6700306 0.07152 6.2% 0.67 [0.53, 0.81]
Byun et al.,2019d 1.4365447 0.07152 6.2% 1.44 [1.30, 1.58]
Byun et al.,2019e 1.5890269 0.07152 6.2% 1.59 [1.45, 1.73]
Hartmann et al.,2009①a 3.8002012 0.215666 5.8% 3.80 [3.38, 4.22]
Hartmann et al.,2009①b 2.6466524 0.215666 5.8% 2.65 [2.22, 3.07]
Hartmann et al.,2009①c 3.8002012 0.215666 5.8% 3.80 [3.38, 4.22]
Matikainen-Tervola E et al.,2024 0.43561122 0.202031 5.9% 0.44 [0.04, 0.83]
Subtotal (95% CI) 60.4% 1.79 [1.30, 2.27]
Heterogeneity: Tau² = 0.60; Chi² = 592.52, df = 9 (P < 0.00001); I² = 98%
Test for overall effect: Z = 7.17 (P < 0.00001)
1.2.2 Ankle/foot
Álvarez MN et al.,2023 1.39837549 0.19802951 5.9% 1.40 [1.01, 1.79]
Rantalainen et al.,2019 2.2975599 0.19803 5.9% 2.30 [1.91, 2.69]
Rogan et al.,2017a 0.3768859 0.226455 5.8% 0.38 [-0.07, 0.82]
Rogan et al.,2017b 1.9459101 0.226455 5.8% 1.95 [1.50, 2.39]
Rudisch et al.,2021a 3.2503945 0.308607 5.4% 3.25 [2.65, 3.86]
Rudisch et al.,2021b 2.9030692 0.308607 5.4% 2.90 [2.30, 3.51]
Rudisch et al.,2021c 2.0438786 0.308607 5.4% 2.04 [1.44, 2.65]
Subtotal (95% CI) 39.6% 2.01 [1.33, 2.70]
Heterogeneity: Tau² = 0.78; Chi² = 85.43, df = 6 (P < 0.00001); I² = 93%
Test for overall effect: Z = 5.80 (P < 0.00001)
Total (95% CI) 100.0% 1.88 [1.49, 2.26]
Heterogeneity: Tau² = 0.63; Chi² = 710.55, df = 16 (P < 0.00001); I² = 98%
Test for overall effect: Z = 9.48 (P < 0.00001)
Test for subgroup differences: Chi² = 0.29, df = 1 (P = 0.59), I² = 0%
-4 -2 0 2 4
Negative correlation Positive correlation
C
Study or Subgroup
Fisher' s Z
SE
Weight
Fisher' s Z IV, Random, 95% CI
Fisher' s Z IV, Random, 95% CI
Maggio et al.,2016a 0.7250051 0.123091 19.3% 0.73 [0.48, 0.97]
Maggio et al.,2016b 0.9287274 0.1 19.8% 0.93 [0.73, 1.12]
Contreras C et al.,2024 1.33307963 0.24253563 15.7% 1.33 [0.86, 1.81]
Byun et al.,2016 1.5392841 0.112509 19.6% 1.54 [1.32, 1.76]
Kuntapun et al., 2020a 1.9459101 0.333333 12.8% 1.95 [1.29, 2.60]
Kuntapun et al., 2020b 1.9459101 0.333333 12.8% 1.95 [1.29, 2.60]
Total (95% CI) 100.0% 1.33 [0.96, 1.71]
Heterogeneity: Tau² = 0.17; Chi² = 40.75, df = 5 (P < 0.00001); I² = 88%
Test for overall effect: Z = 6.97 (P < 0.00001)
-2 -1 0 1 2
Negative correlation Positive correlation

Figure 1. Subgroup analysis on the validity of walking speed measured by IMUs. A represented intraclass correlation coefficients (ICCs) based on speed subgroup; B represented ICCs based on location subgroup; C represented Pearson correlation coefficients(r). Byun et al.,2019a (model 1), b(model 2), c(model 3), d(model 4), e(model 5). Rogan et al.,2017a (preferred speed), b (fast speed). Rudisch et al.,2021a (IMUs vs GAITRite), b (IMUs vs Optogait), c (IMUs vs Zebris). Kuntapun et al., 2020a(back), b(shoulder bag). Maggio et al.,2016a(men),b(women). *SE* standard error, *IV* inverse variance, *CI* confidence interval.

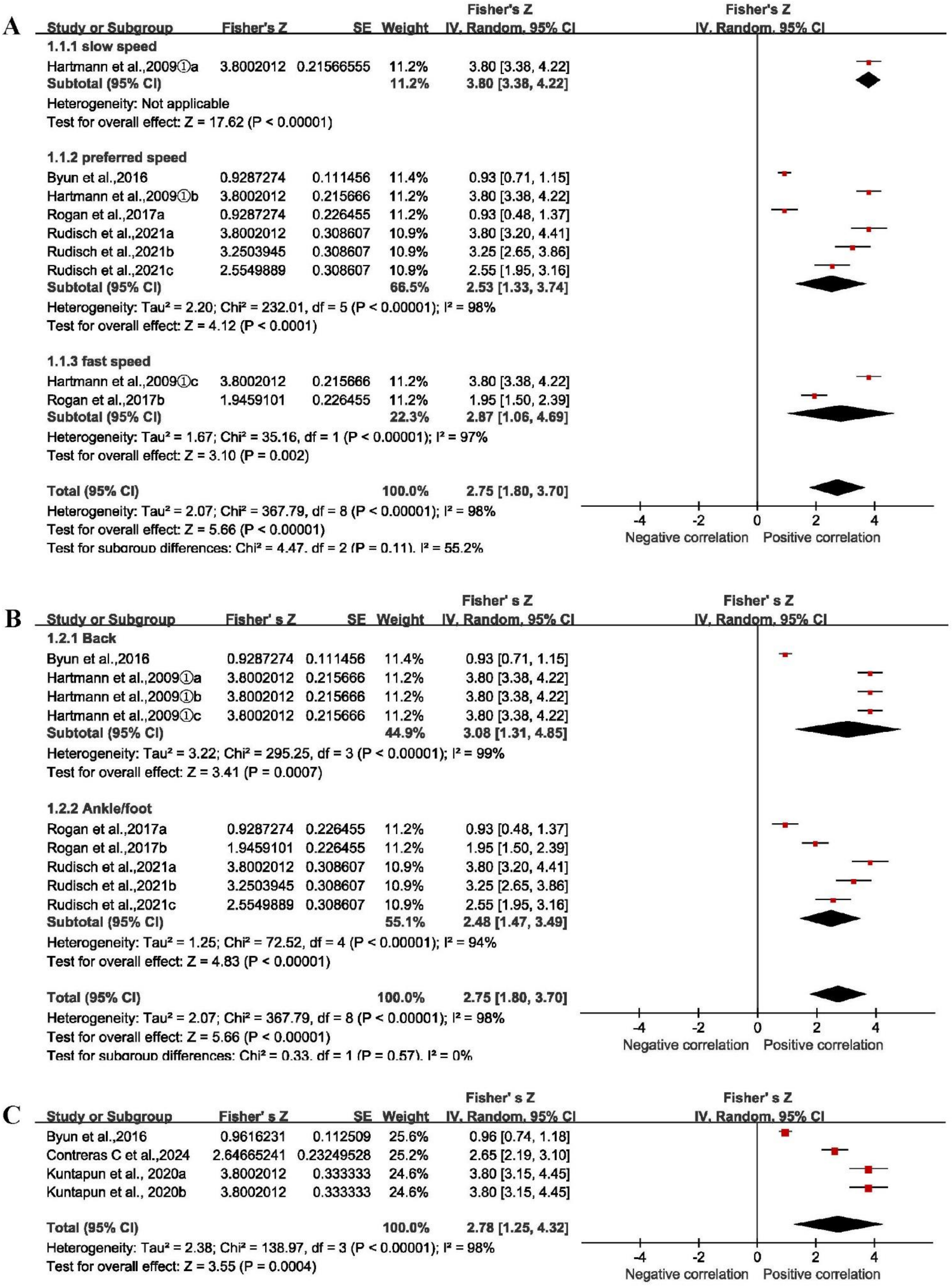

Figure 2. Subgroup analysis on the validity of cadence measured by IMUs. A represented intraclass correlation coefficients (ICCs) based on speed subgroup; B represented ICCs based on location subgroup; C represented Pearson correlation coefficients(r). Hartmann et al.,2009a(slow speed), b(preferred speed), c(fast speed). Byun et al.,2019a(model 1), b(model 2), c(model 3), d(model 4), e(model 5). Rogan et al.,2017a(preferred speed), b(fast speed). Rudisch et al.,2021a(IMUs vs GAITRite),b(IMUs vs Optogait),c(IMUs vs Zebris). Kuntapun et al., 2020a(back), b(shoulder bag). *SE* standard error, *IV* inverse variance, *CI* confidence interval.

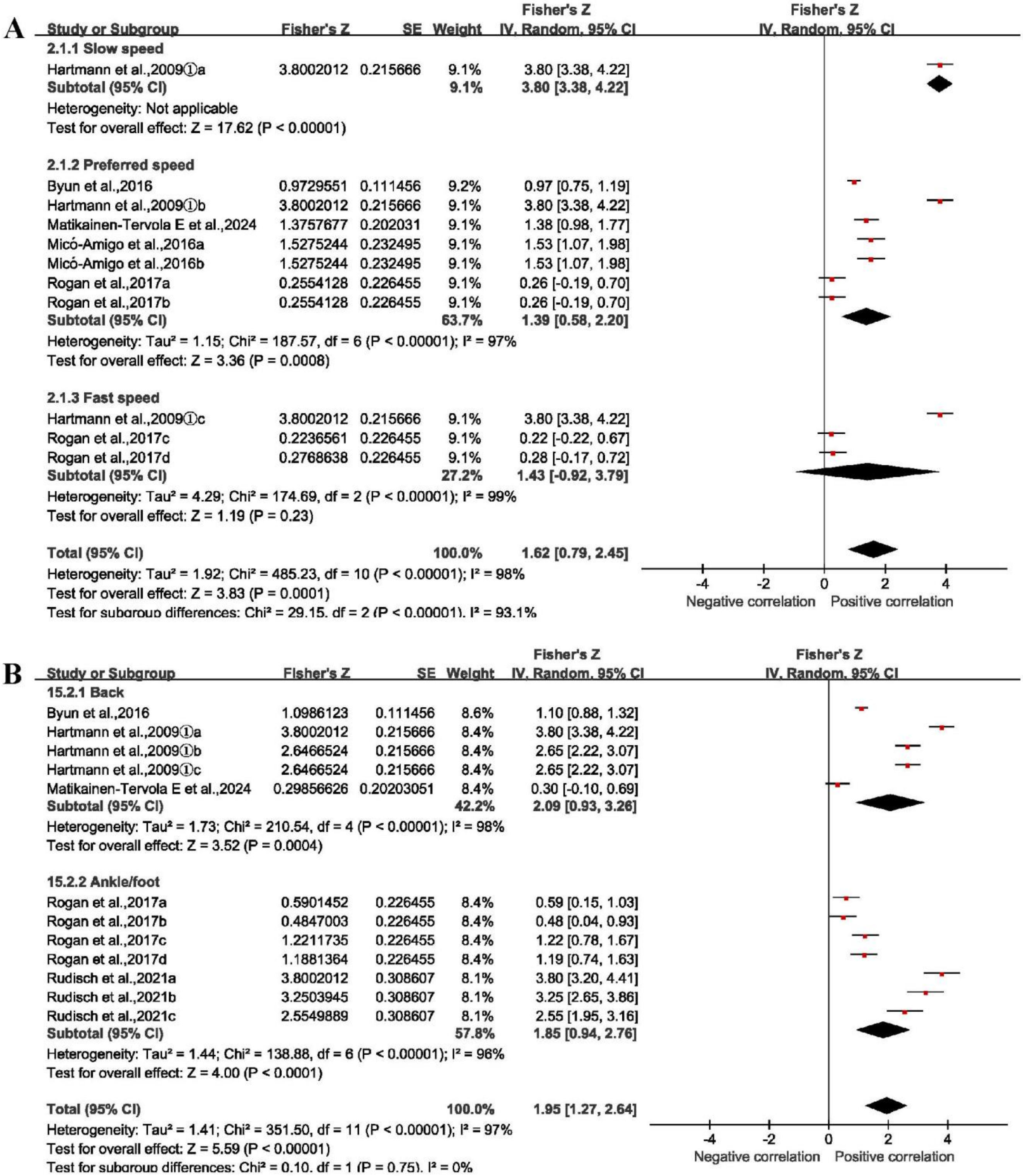

Figure3. Subgroup analysis on the validity of step time measured by IMUs. A represented intraclass correlation coefficients (ICCs) based on speed subgroup; B represented ICCs based on location subgroup; Hartmann et al.,2009a (slow speed), b (preferred speed), c (fast speed). Micó-Amigo et al.,2016a (back), b(foot). Rogan et al.,2017a (preferred speed-left), b (preferred speed-right), c (fast speed-left), d (fast speed-right). *SE* standard error, *IV* inverse variance, *CI* confidence interval.

A
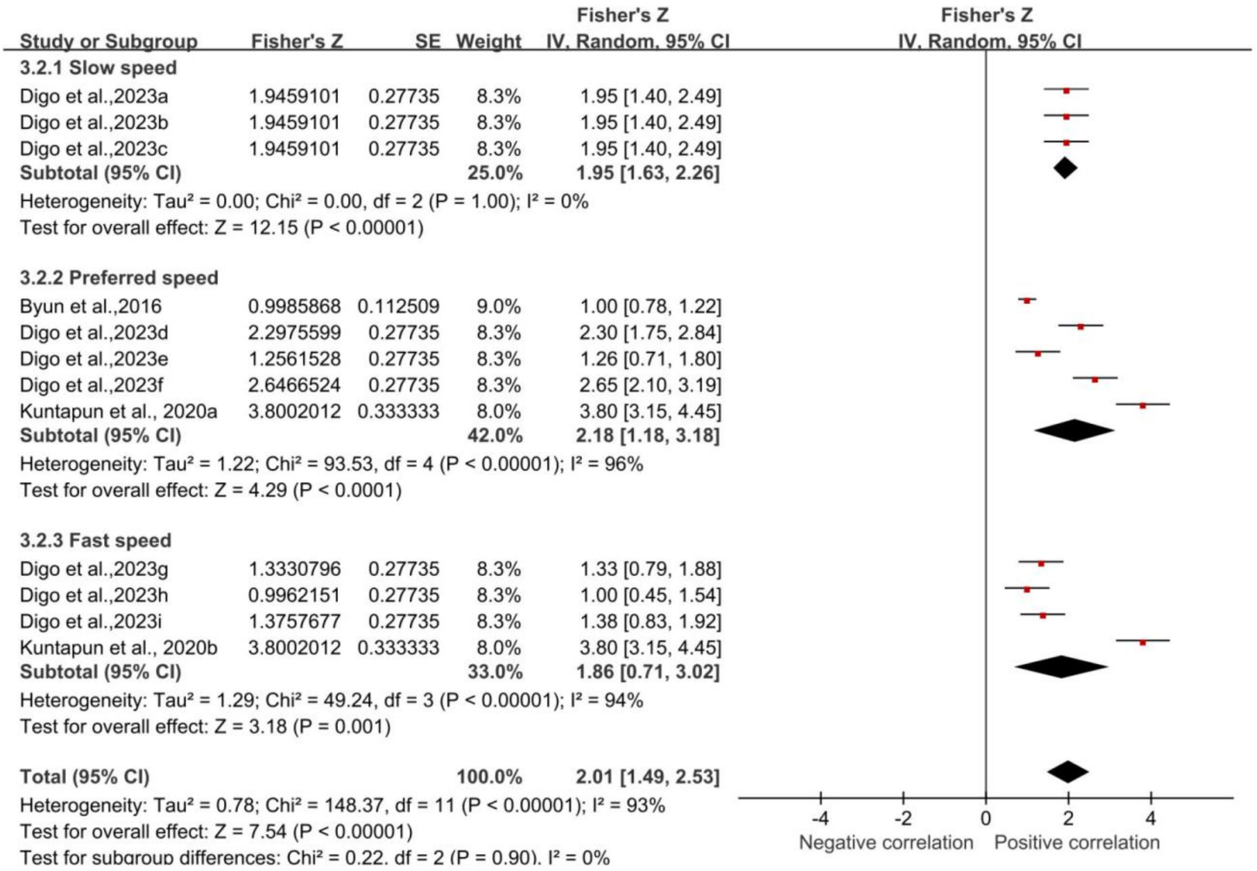

B
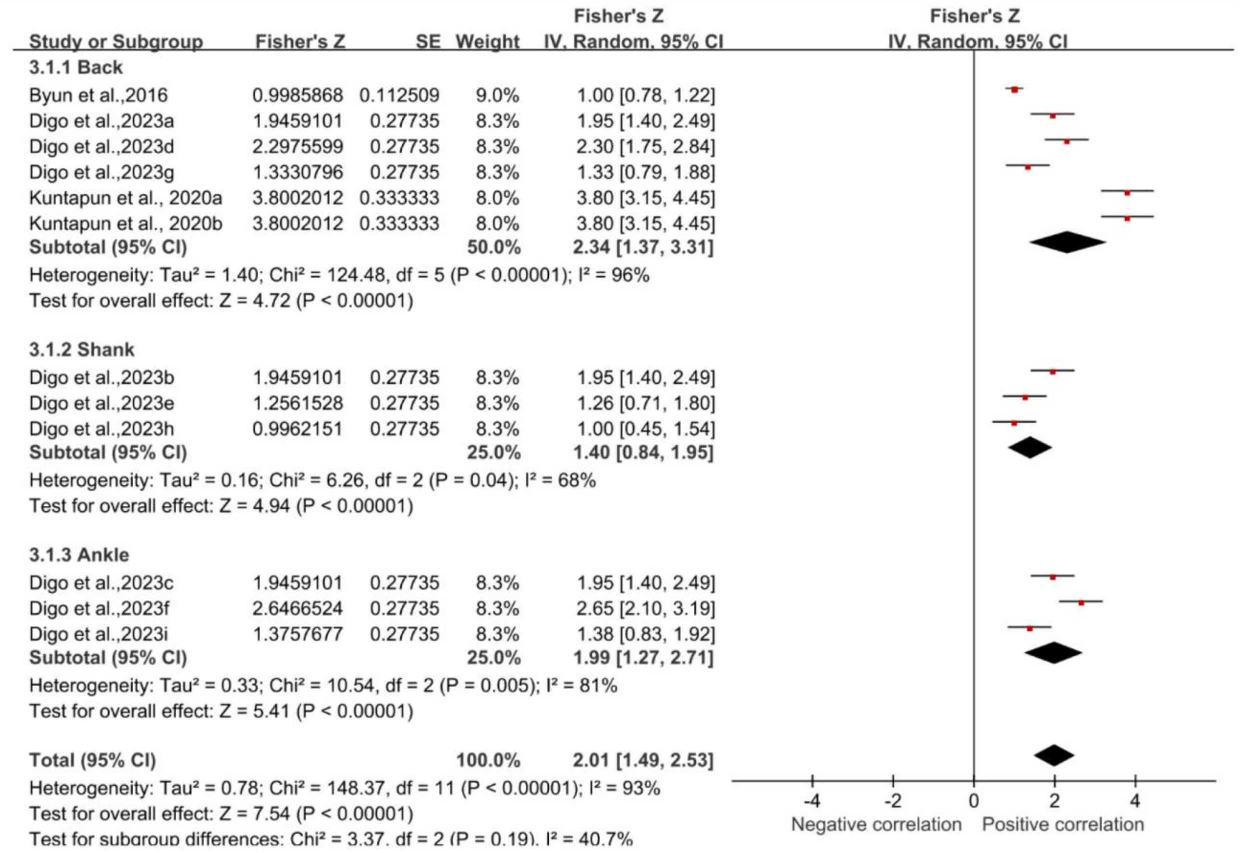

Figure 4. Subgroup analysis on the validity of step time measured by IMUs. A represented Pearson correlation coefficients(r) based on speed subgroup; B represented Pearson correlation coefficients(r) based on location subgroup. Digo et al., 2023a(slow speed, trunk), b(slow speed, shank), c(slow speed, ankle), d(normal speed, trunk),e(normal speed, shank),

f(normal speed, ankle), g(fast speed, trunk), h(fast speed, shank), i(fast speed, ankle). Kuntapun et al., 2020a(back), b(shoulder bag). *SE* standard error, *IV* inverse variance, *CI* confidence interval.

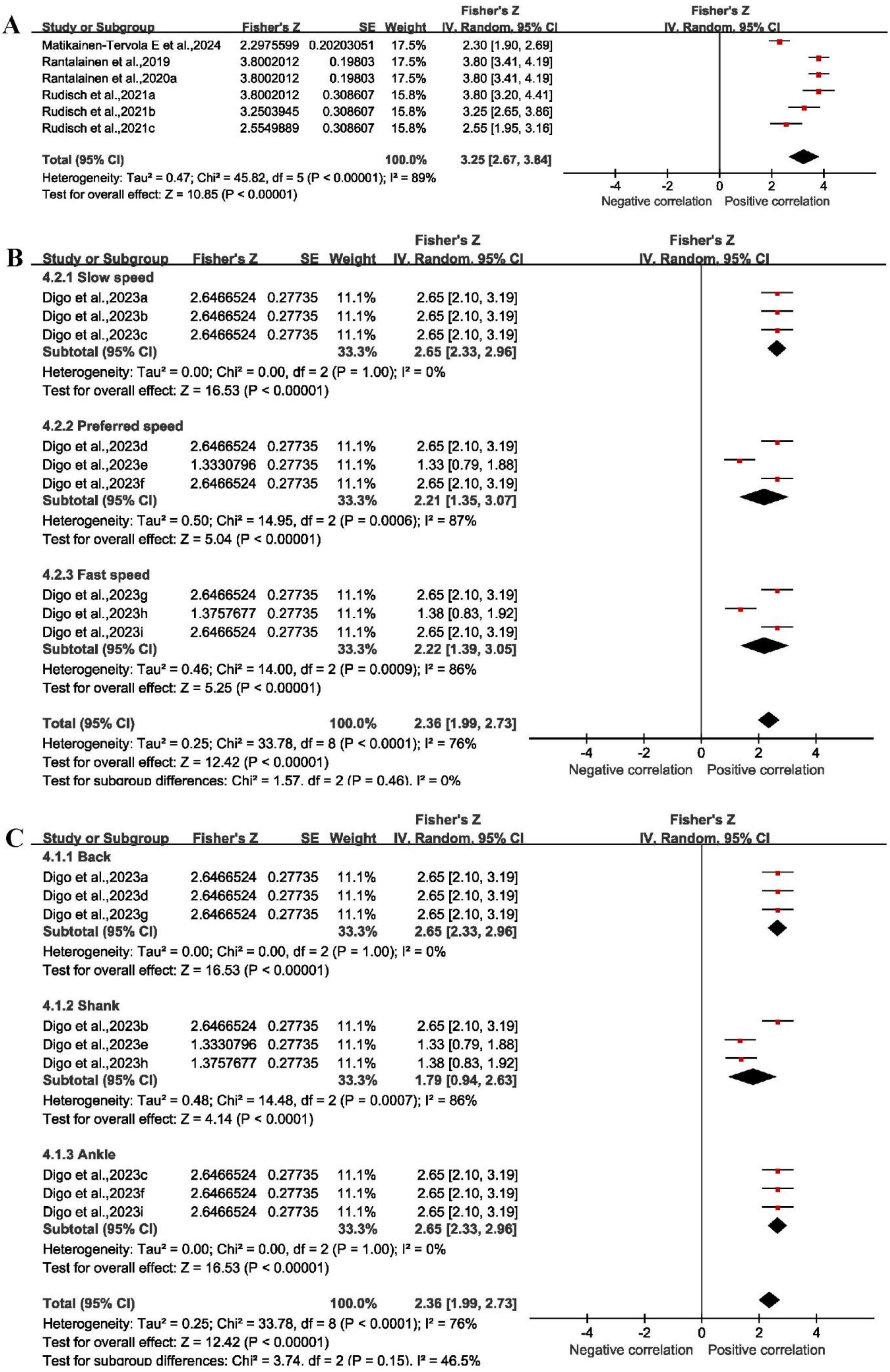
A
Study or Subgroup
Fisher's Z
SE
Weight
Fisher's Z IV, Random, 95% CI
Fisher's Z IV, Random, 95% CI
Matikainen-Tervola E et al.,2024 2.2975599 0.20203051 17.5% 2.30 [1.90, 2.69]
Rantalainen et al.,2019 3.8002012 0.19803 17.5% 3.80 [3.41, 4.19]
Rantalainen et al.,2020a 3.8002012 0.19803 17.5% 3.80 [3.41, 4.19]
Rudisch et al.,2021a 3.8002012 0.308607 15.8% 3.80 [3.20, 4.41]
Rudisch et al.,2021b 3.2503945 0.308607 15.8% 3.25 [2.65, 3.86]
Rudisch et al.,2021c 2.5549889 0.308607 15.8% 2.55 [1.95, 3.16]
Total (95% CI) 100.0% 3.25 [2.67, 3.84]
Heterogeneity: Tau² = 0.47; Chi² = 45.82, df = 5 (P < 0.00001); I² = 89%
Test for overall effect: Z = 10.85 (P < 0.00001)
-4 -2 0 2 4
Negative correlation Positive correlation
B
Study or Subgroup
Fisher's Z
SE
Weight
Fisher's Z IV, Random, 95% CI
Fisher's Z IV, Random, 95% CI
4.2.1 Slow speed
Digo et al.,2023a 2.6466524 0.27735 11.1% 2.65 [2.10, 3.19]
Digo et al.,2023b 2.6466524 0.27735 11.1% 2.65 [2.10, 3.19]
Digo et al.,2023c 2.6466524 0.27735 11.1% 2.65 [2.10, 3.19]
Subtotal (95% CI) 33.3% 2.65 [2.33, 2.96]
Heterogeneity: Tau² = 0.00; Chi² = 0.00, df = 2 (P = 1.00); I² = 0%
Test for overall effect: Z = 16.53 (P < 0.00001)
4.2.2 Preferred speed
Digo et al.,2023d 2.6466524 0.27735 11.1% 2.65 [2.10, 3.19]
Digo et al.,2023e 1.3330796 0.27735 11.1% 1.33 [0.79, 1.88]
Digo et al.,2023f 2.6466524 0.27735 11.1% 2.65 [2.10, 3.19]
Subtotal (95% CI) 33.3% 2.21 [1.35, 3.07]
Heterogeneity: Tau² = 0.50; Chi² = 14.95, df = 2 (P = 0.0006); I² = 87%
Test for overall effect: Z = 5.04 (P < 0.00001)
4.2.3 Fast speed
Digo et al.,2023g 2.6466524 0.27735 11.1% 2.65 [2.10, 3.19]
Digo et al.,2023h 1.3757677 0.27735 11.1% 1.38 [0.83, 1.92]
Digo et al.,2023i 2.6466524 0.27735 11.1% 2.65 [2.10, 3.19]
Subtotal (95% CI) 33.3% 2.22 [1.39, 3.05]
Heterogeneity: Tau² = 0.46; Chi² = 14.00, df = 2 (P = 0.0009); I² = 86%
Test for overall effect: Z = 5.25 (P < 0.00001)
Total (95% CI) 100.0% 2.36 [1.99, 2.73]
Heterogeneity: Tau² = 0.25; Chi² = 33.78, df = 8 (P < 0.0001); I² = 76%
Test for overall effect: Z = 12.42 (P < 0.00001)
Test for subgroup differences: Chi² = 1.57, df = 2 (P = 0.46), I² = 0%
-4 -2 0 2 4
Negative correlation Positive correlation
C
Study or Subgroup
Fisher's Z
SE
Weight
Fisher's Z IV, Random, 95% CI
Fisher's Z IV, Random, 95% CI
4.1.1 Back
Digo et al.,2023a 2.6466524 0.27735 11.1% 2.65 [2.10, 3.19]
Digo et al.,2023d 2.6466524 0.27735 11.1% 2.65 [2.10, 3.19]
Digo et al.,2023g 2.6466524 0.27735 11.1% 2.65 [2.10, 3.19]
Subtotal (95% CI) 33.3% 2.65 [2.33, 2.96]
Heterogeneity: Tau² = 0.00; Chi² = 0.00, df = 2 (P = 1.00); I² = 0%
Test for overall effect: Z = 16.53 (P < 0.00001)
4.1.2 Shank
Digo et al.,2023b 2.6466524 0.27735 11.1% 2.65 [2.10, 3.19]
Digo et al.,2023e 1.3330796 0.27735 11.1% 1.33 [0.79, 1.88]
Digo et al.,2023h 1.3757677 0.27735 11.1% 1.38 [0.83, 1.92]
Subtotal (95% CI) 33.3% 1.79 [0.94, 2.63]
Heterogeneity: Tau² = 0.48; Chi² = 14.48, df = 2 (P = 0.0007); I² = 86%
Test for overall effect: Z = 4.14 (P < 0.0001)
4.1.3 Ankle
Digo et al.,2023c 2.6466524 0.27735 11.1% 2.65 [2.10, 3.19]
Digo et al.,2023f 2.6466524 0.27735 11.1% 2.65 [2.10, 3.19]
Digo et al.,2023i 2.6466524 0.27735 11.1% 2.65 [2.10, 3.19]
Subtotal (95% CI) 33.3% 2.65 [2.33, 2.96]
Heterogeneity: Tau² = 0.00; Chi² = 0.00, df = 2 (P = 1.00); I² = 0%
Test for overall effect: Z = 16.53 (P < 0.00001)
Total (95% CI) 100.0% 2.36 [1.99, 2.73]
Heterogeneity: Tau² = 0.25; Chi² = 33.78, df = 8 (P < 0.0001); I² = 76%
Test for overall effect: Z = 12.42 (P < 0.00001)
Test for subgroup differences: Chi² = 3.74, df = 2 (P = 0.15), I² = 46.5%
-4 -2 0 2 4
Negative correlation Positive correlation

Figure5. Forest plot on the validity of stride time measured by IMUs. A represented intraclass correlation coefficients (ICCs). B represented Pearson correlation coefficients(r) based on speed subgroup; C represented Pearson correlation coefficients(r) based on location subgroup. Rudisch et al.,2021a(IMUs vs GAITRite),b(IMUs vs Optogait),c(IMUs vs Zebris). Rantalainen et al.,2020a(ankle). Digo et al.,2023a(slow,trunk),b(slow,shank),c(slow,ankle),d(normal,trunk),e(normal,shank),f(normal,ankle), g(fast,trunk), h(fast, shank), i(fast, ankle). SE standard error, IV inverse variance, CI confidence interval.

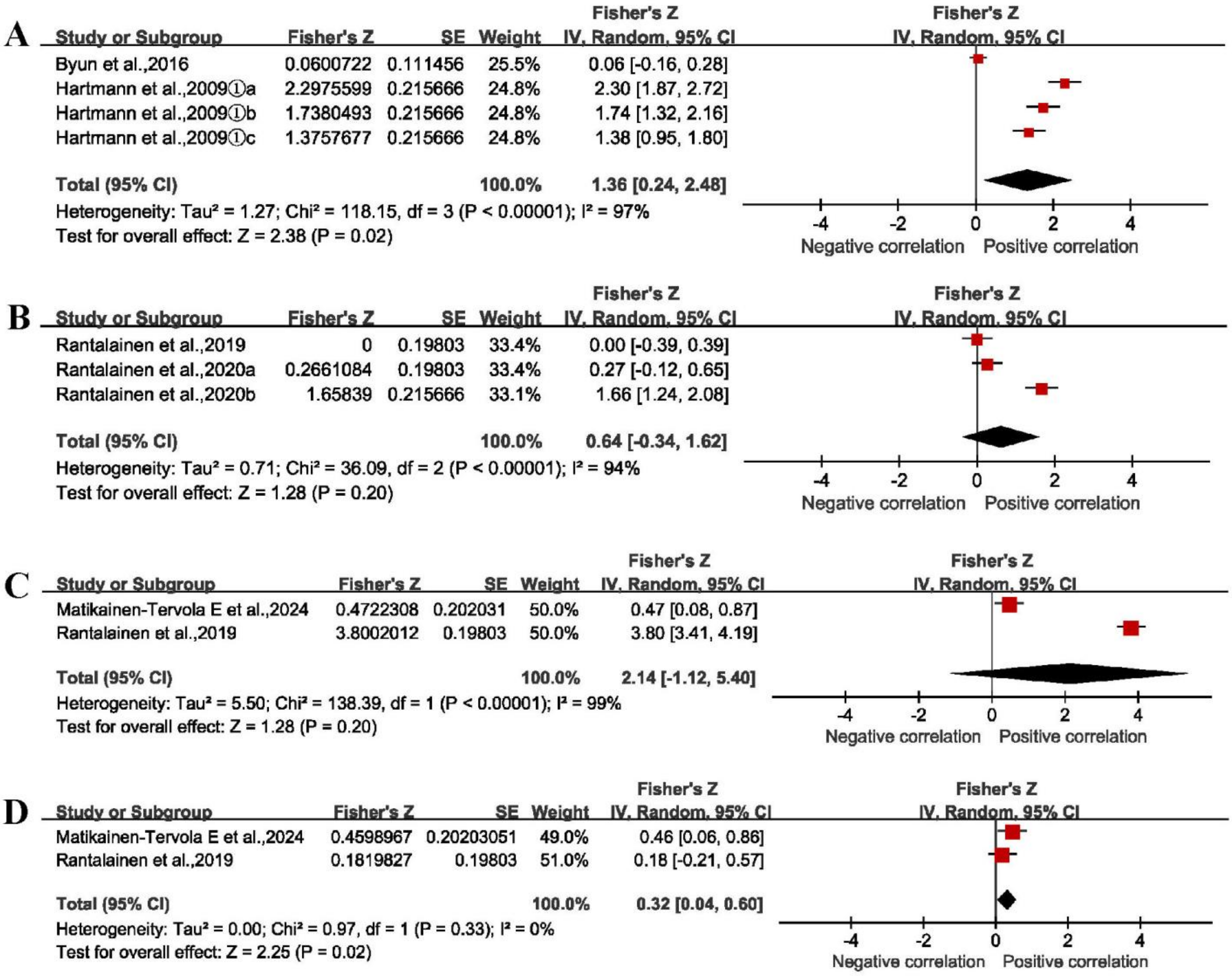

Figure 6. Forest plot on the validity of (A) step time variability, (B) stride time SD; (C) stance time and (D) swing time. Hartmann et al.,2009①a(slow speed), ①b(preferred speed), ①c(fast speed). Rantalainen et al.,2020a(ankle), b(back). *SE* standard error, *IV* inverse variance, *CI* confidence interval.

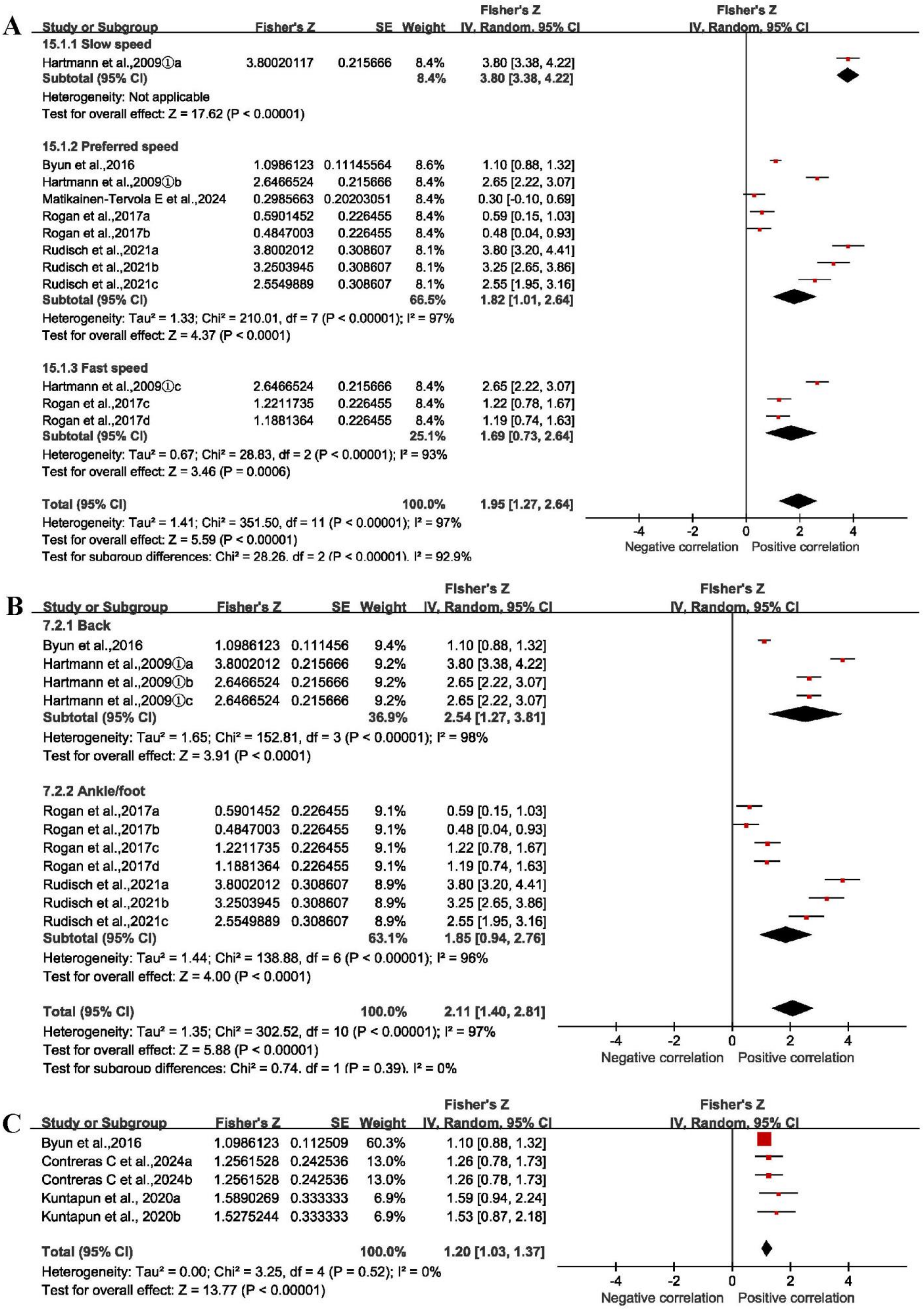
A
Study or Subgroup | Fisher's Z | SE | Weight | Fisher's Z IV, Random, 95% CI | Fisher's Z IV, Random, 95% CI
15.1.1 Slow speed
Hartmann et al.,2009①a 3.80020117 0.215666 8.4% 3.80 [3.38, 4.22]
Subtotal (95% CI) 8.4% 3.80 [3.38, 4.22]
Heterogeneity: Not applicable
Test for overall effect: Z = 17.62 (P < 0.00001)
15.1.2 Preferred speed
Byun et al.,2016 1.0986123 0.11145564 8.6% 1.10 [0.88, 1.32]
Hartmann et al.,2009①b 2.6466524 0.215666 8.4% 2.65 [2.22, 3.07]
Matikainen-Tervola E et al.,2024 0.2985663 0.20203051 8.4% 0.30 [-0.10, 0.69]
Rogan et al.,2017a 0.5901452 0.226455 8.4% 0.59 [0.15, 1.03]
Rogan et al.,2017b 0.4847003 0.226455 8.4% 0.48 [0.04, 0.93]
Rudisch et al.,2021a 3.8002012 0.308607 8.1% 3.80 [3.20, 4.41]
Rudisch et al.,2021b 3.2503945 0.308607 8.1% 3.25 [2.65, 3.86]
Rudisch et al.,2021c 2.5549889 0.308607 8.1% 2.55 [1.95, 3.16]
Subtotal (95% CI) 66.5% 1.82 [1.01, 2.64]
Heterogeneity: Tau² = 1.33; Chi² = 210.01, df = 7 (P < 0.00001); I² = 97%
Test for overall effect: Z = 4.37 (P < 0.0001)
15.1.3 Fast speed
Hartmann et al.,2009①c 2.6466524 0.215666 8.4% 2.65 [2.22, 3.07]
Rogan et al.,2017c 1.2211735 0.226455 8.4% 1.22 [0.78, 1.67]
Rogan et al.,2017d 1.1881364 0.226455 8.4% 1.19 [0.74, 1.63]
Subtotal (95% CI) 25.1% 1.69 [0.73, 2.64]
Heterogeneity: Tau² = 0.67; Chi² = 28.83, df = 2 (P < 0.00001); I² = 93%
Test for overall effect: Z = 3.46 (P = 0.0006)
Total (95% CI) 100.0% 1.95 [1.27, 2.64]
Heterogeneity: Tau² = 1.41; Chi² = 351.50, df = 11 (P < 0.00001); I² = 97%
Test for overall effect: Z = 5.59 (P < 0.00001)
Test for subgroup differences: Chi² = 28.26, df = 2 (P < 0.00001), I² = 92.9%
-4 -2 0 2 4
Negative correlation Positive correlation
B
Study or Subgroup | Fisher's Z | SE | Weight | Fisher's Z IV, Random, 95% CI | Fisher's Z IV, Random, 95% CI
7.2.1 Back
Byun et al.,2016 1.0986123 0.111456 9.4% 1.10 [0.88, 1.32]
Hartmann et al.,2009①a 3.8002012 0.215666 9.2% 3.80 [3.38, 4.22]
Hartmann et al.,2009①b 2.6466524 0.215666 9.2% 2.65 [2.22, 3.07]
Hartmann et al.,2009①c 2.6466524 0.215666 9.2% 2.65 [2.22, 3.07]
Subtotal (95% CI) 36.9% 2.54 [1.27, 3.81]
Heterogeneity: Tau² = 1.65; Chi² = 152.81, df = 3 (P < 0.00001); I² = 98%
Test for overall effect: Z = 3.91 (P < 0.0001)
7.2.2 Ankle/foot
Rogan et al.,2017a 0.5901452 0.226455 9.1% 0.59 [0.15, 1.03]
Rogan et al.,2017b 0.4847003 0.226455 9.1% 0.48 [0.04, 0.93]
Rogan et al.,2017c 1.2211735 0.226455 9.1% 1.22 [0.78, 1.67]
Rogan et al.,2017d 1.1881364 0.226455 9.1% 1.19 [0.74, 1.63]
Rudisch et al.,2021a 3.8002012 0.308607 8.9% 3.80 [3.20, 4.41]
Rudisch et al.,2021b 3.2503945 0.308607 8.9% 3.25 [2.65, 3.86]
Rudisch et al.,2021c 2.5549889 0.308607 8.9% 2.55 [1.95, 3.16]
Subtotal (95% CI) 63.1% 1.85 [0.94, 2.76]
Heterogeneity: Tau² = 1.44; Chi² = 138.88, df = 6 (P < 0.00001); I² = 96%
Test for overall effect: Z = 4.00 (P < 0.0001)
Total (95% CI) 100.0% 2.11 [1.40, 2.81]
Heterogeneity: Tau² = 1.35; Chi² = 302.52, df = 10 (P < 0.00001); I² = 97%
Test for overall effect: Z = 5.88 (P < 0.00001)
Test for subgroup differences: Chi² = 0.74, df = 1 (P = 0.39), I² = 0%
-4 -2 0 2 4
Negative correlation Positive correlation
C
Study or Subgroup | Fisher's Z | SE | Weight | Fisher's Z IV, Random, 95% CI | Fisher's Z IV, Random, 95% CI
Byun et al.,2016 1.0986123 0.112509 60.3% 1.10 [0.88, 1.32]
Contreras C et al.,2024a 1.2561528 0.242536 13.0% 1.26 [0.78, 1.73]
Contreras C et al.,2024b 1.2561528 0.242536 13.0% 1.26 [0.78, 1.73]
Kuntapun et al., 2020a 1.5890269 0.333333 6.9% 1.59 [0.94, 2.24]
Kuntapun et al., 2020b 1.5275244 0.333333 6.9% 1.53 [0.87, 2.18]
Total (95% CI) 100.0% 1.20 [1.03, 1.37]
Heterogeneity: Tau² = 0.00; Chi² = 3.25, df = 4 (P = 0.52); I² = 0%
Test for overall effect: Z = 13.77 (P < 0.00001)
-4 -2 0 2 4
Negative correlation Positive correlation

Figure 7. Subgroup analysis on the validity of step length measured by IMUs. A represented intraclass correlation coefficients (ICCs) based on speed subgroup; B represented ICCs based on location subgroup; C represented Pearson correlation coefficients(r) based on speed subgroup; D represented Pearson correlation coefficients(r) based on location subgroup. Hartmann et al.,2009a(slow speed), b(preferred speed), c(fast speed). Rogan et al.,2017a (preferred speed-left), b(preferred speed-right),c(fast speed-left), b(fast speed-right). Rudisch et al.,2021a(IMUs vs GAITRite),b(IMUs vs Optogait),c(IMUs vs Zebris). Contreras C et al.,2024a(left), b(right). Kuntapun et al., 2020a(back), b(shoulder bag). *SE* standard error, *IV* inverse variance, *CI* confidence interval.

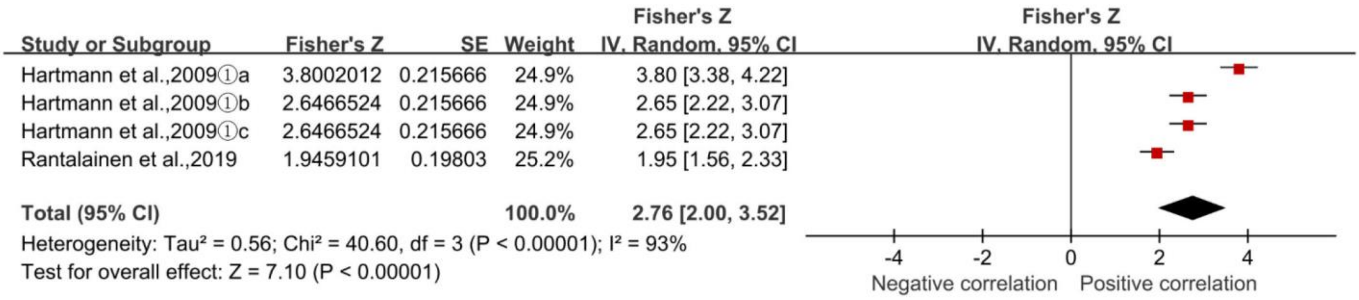

Figure 8. Forest plot on the validity of stride length which represented by intraclass correlation coefficients (ICCs) measured by IMUs. Hartmann et al.,2009a(slow speed), b(preferred speed), c(fast speed). *SE* standard error, *IV* inverse variance, *CI* confidence interval.

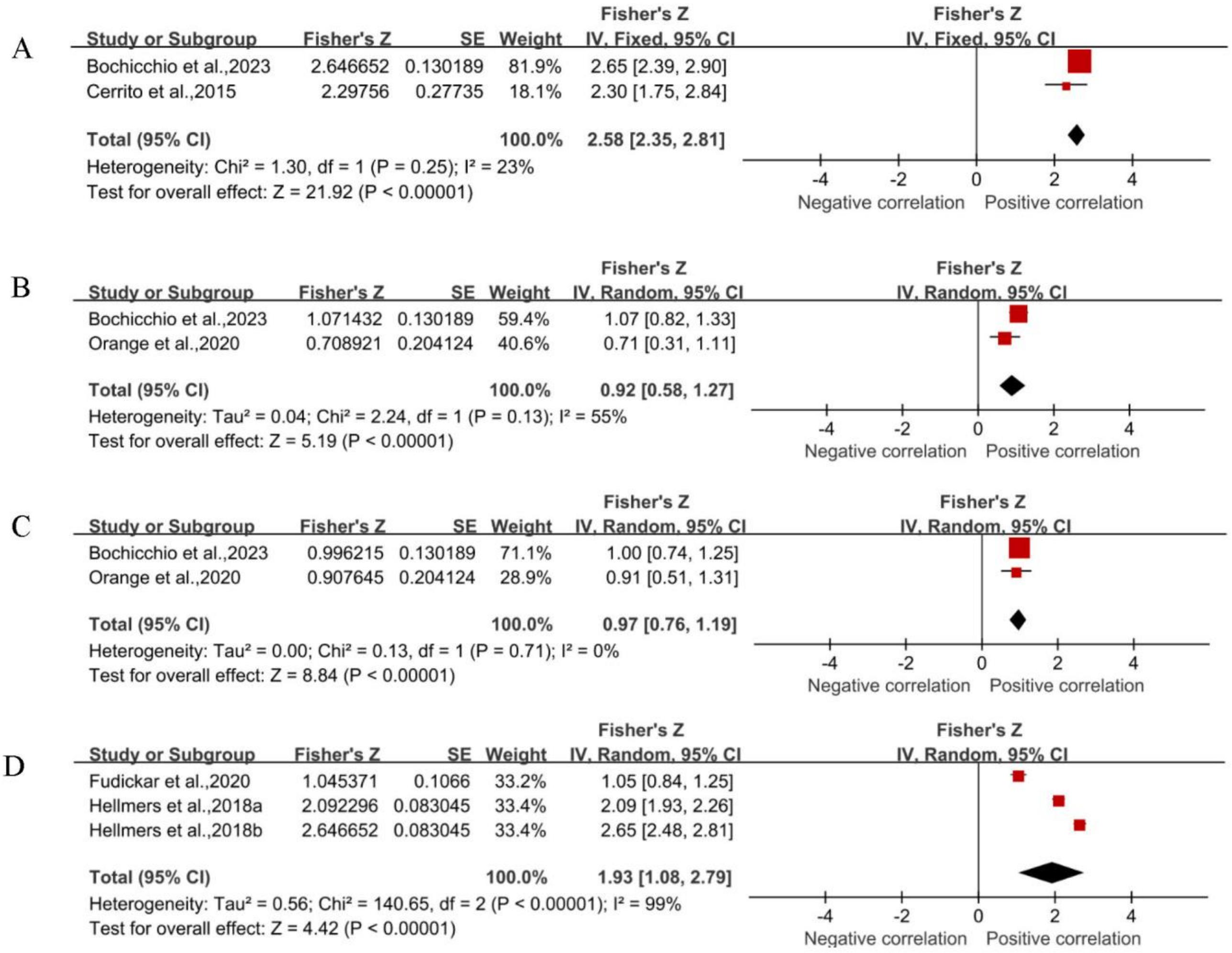

Figure 9. Forest plot on the validity of individual sit to stand duration(A), sit to stand power(B), sit to stand velocity(C) and timed up and go duration(D) which represented by intraclass correlation coefficients (ICCs) measured by IMU. Hellmers et al.,2018a, IMUs vs stopwatch; Hellmers et al.,2018b, IMUs vs automated measurements. *SE* standard error, *IV* inverse variance, *CI* confidence interval.

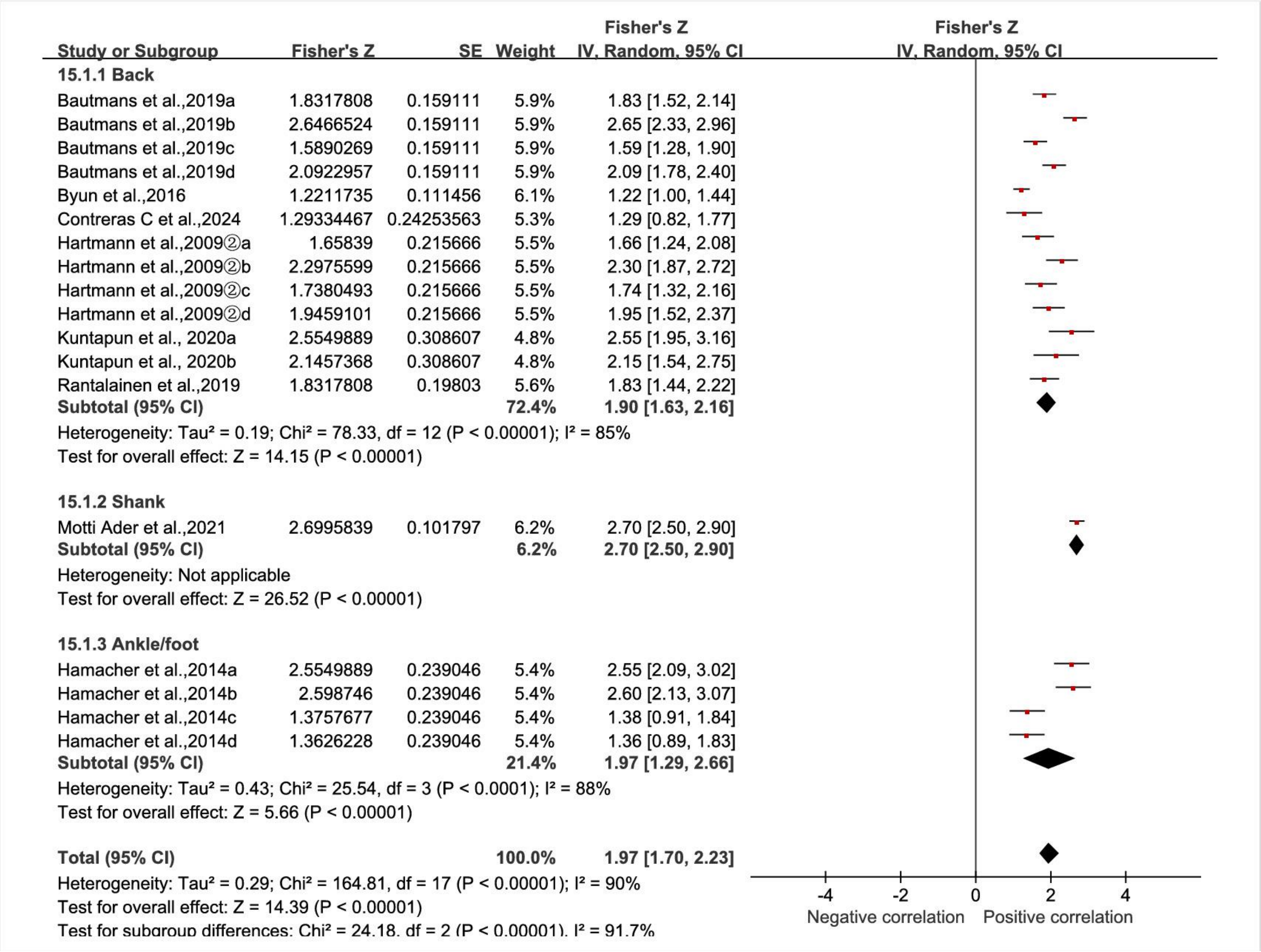

Figure 10. Subgroup analysis on the reliability of walking speed based on location subgroup which represented by intraclass correlation coefficients (ICCs) measured by IMUs. Greene et al.,2022a(Torsor sensor),b(Thigh sensor). Regterschot et al.,2014a(normal speed), b(fast speed). Zhang et al.,2014a(normal speed), b(fast speed). *SE* standard error, *IV* inverse variance, *CI* confidence interval.

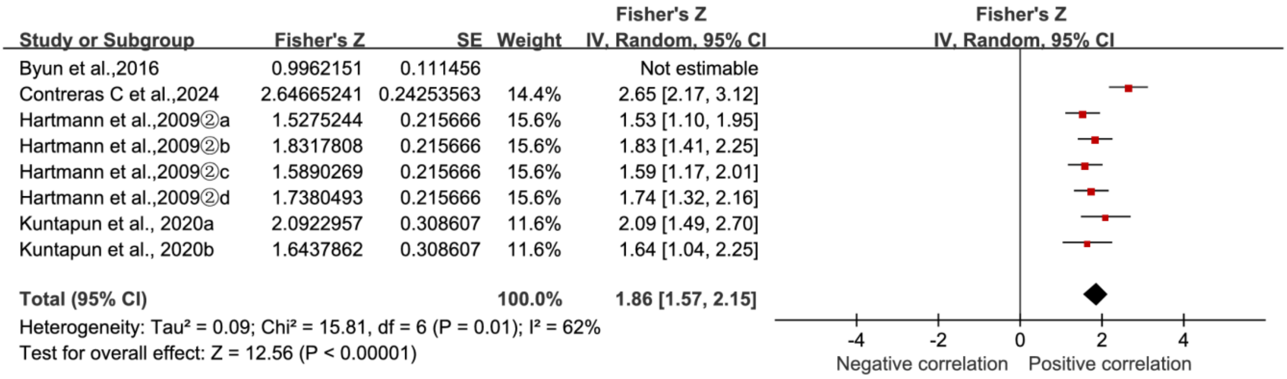

Figure 11. Forest plot on the reliability of cadence which represented by intraclass correlation coefficients (ICCs) measured by IMUs. Hartmann et al.,2009 ② a (Intra-rater, Gym floor), b(Intra-rater,Soft foam rubber),c(Inter-rater,Gym floor),d(Inter-rater,Soft foam rubber). Kuntapun et al., 2020a(back), b (shoulder bag). *SE* standard error, *IV* inverse variance, *CI* confidence interval.

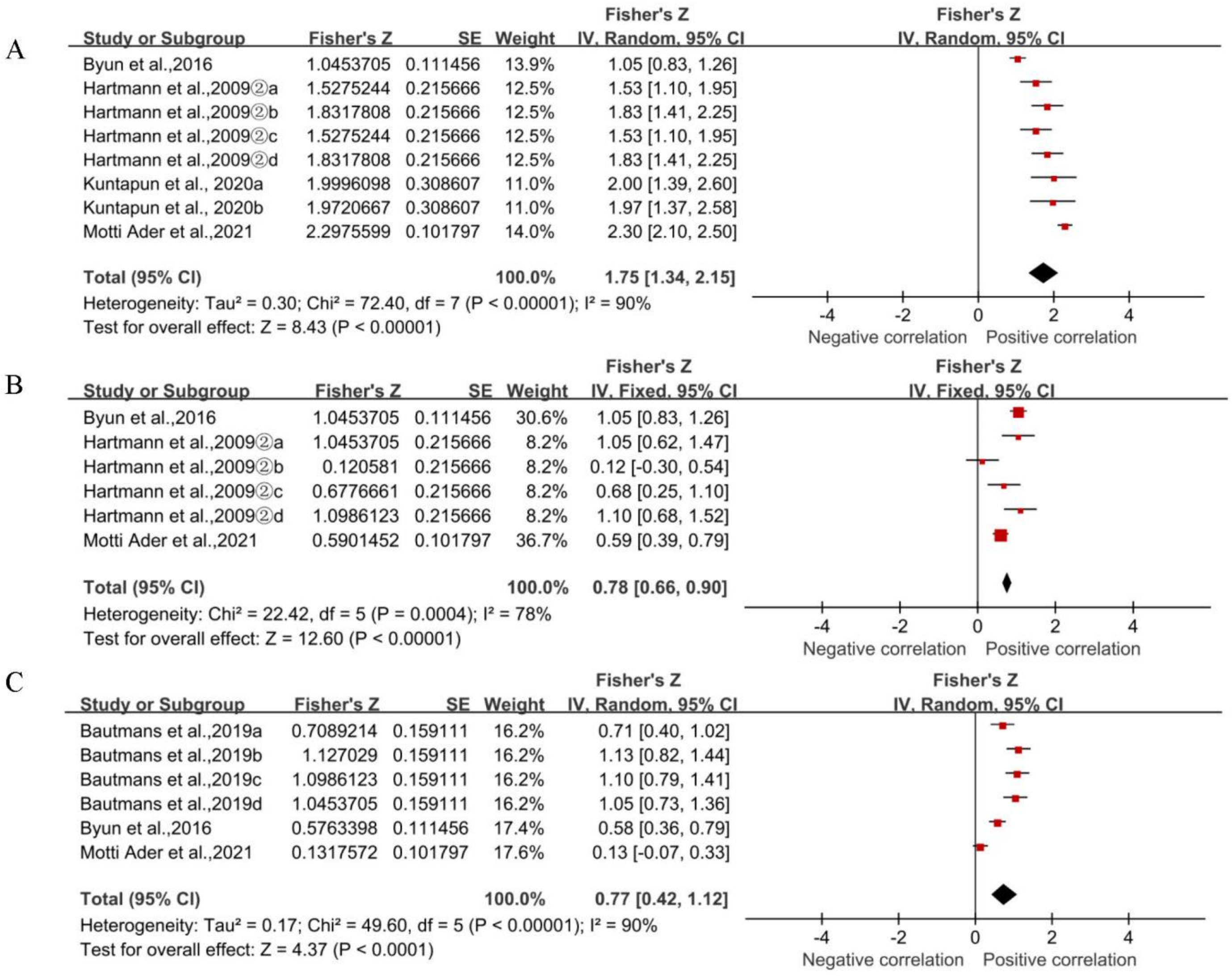

Figure12. Forest plot on the reliability of (A) step time, (B) step time variability and (C) step time asymmetry which represented by intraclass correlation coefficients (ICCs) measured by IMUs. Hartmann et al.,2009②a(Intra-rater,Gym floor),b(Intra-rater,Soft foam rubber),c(Inter-rater,Gym floor),d(Inter-rater,Soft foam rubber). Kuntapun et al., 2020a(back), b(shoulder bag). Hartmann et al.,2009②a(Intra-rater,Gym floor),b(Intra-rater,Soft foam rubber),c(Inter-rater,Gym floor),d(Inter-rater,Soft foam rubber). Bautmans et al.,2019a(Intra-observer,single walk),b(Intra-observer,mean of two walks),c(Inter-observer,single walk),d(Inter-observer,mean of two walks). *SE* standard error, *IV* inverse variance, *CI* confidence interval.

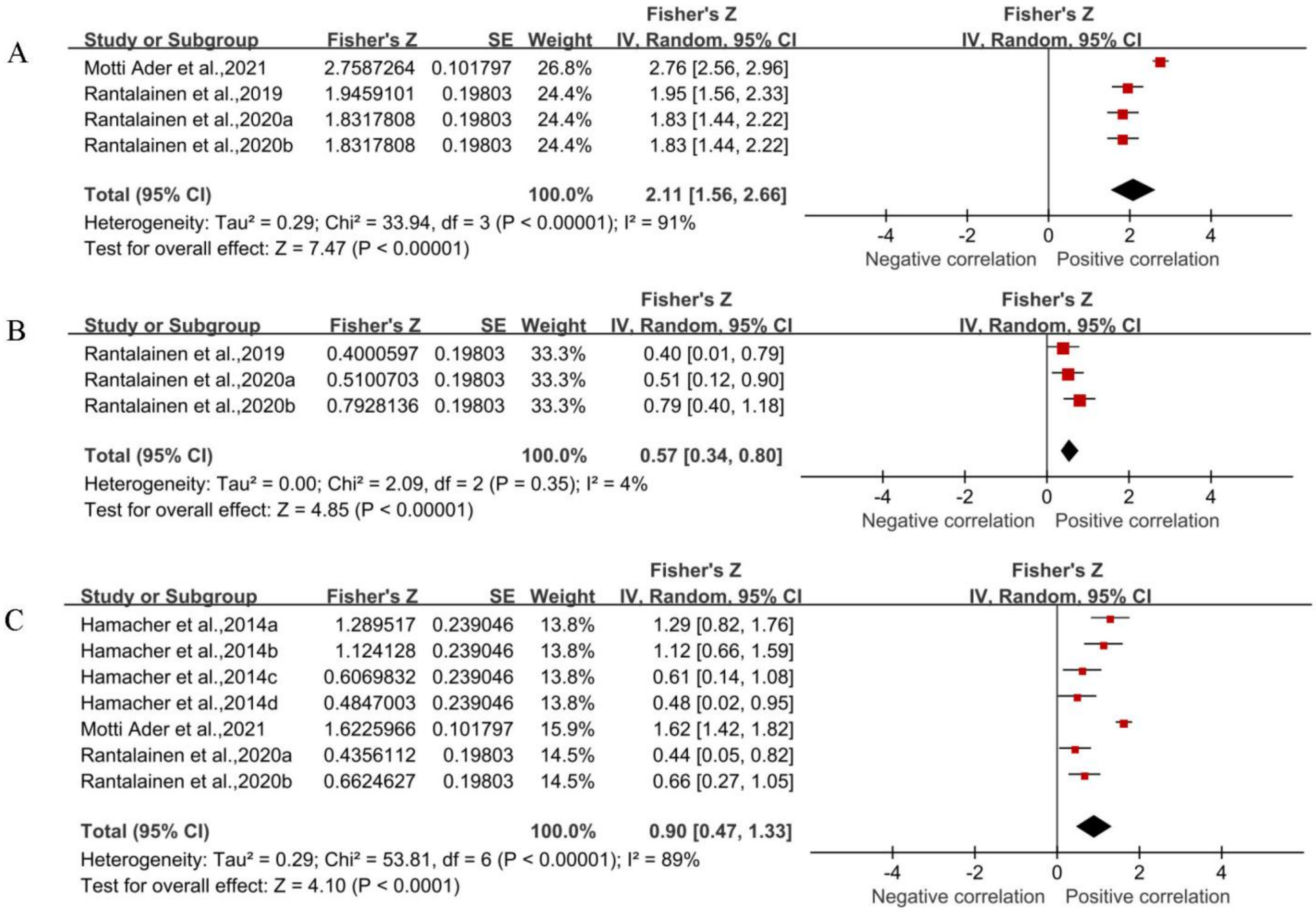

Figure13. Forest plot on the reliability of (A)stride time, (B) stride time SD and (C) stride time variability which represented by intraclass correlation coefficients (ICCs) measured by IMUs. Rantalainen et al.,2020a(ankle), b(waist). Hamacher et al.,2014a(Intra-day, left),b(Intra-day, right),c(Inter-day, left),d(Inter-day, right). *SE* standard error, *IV* inverse variance, *CI* confidence interval.

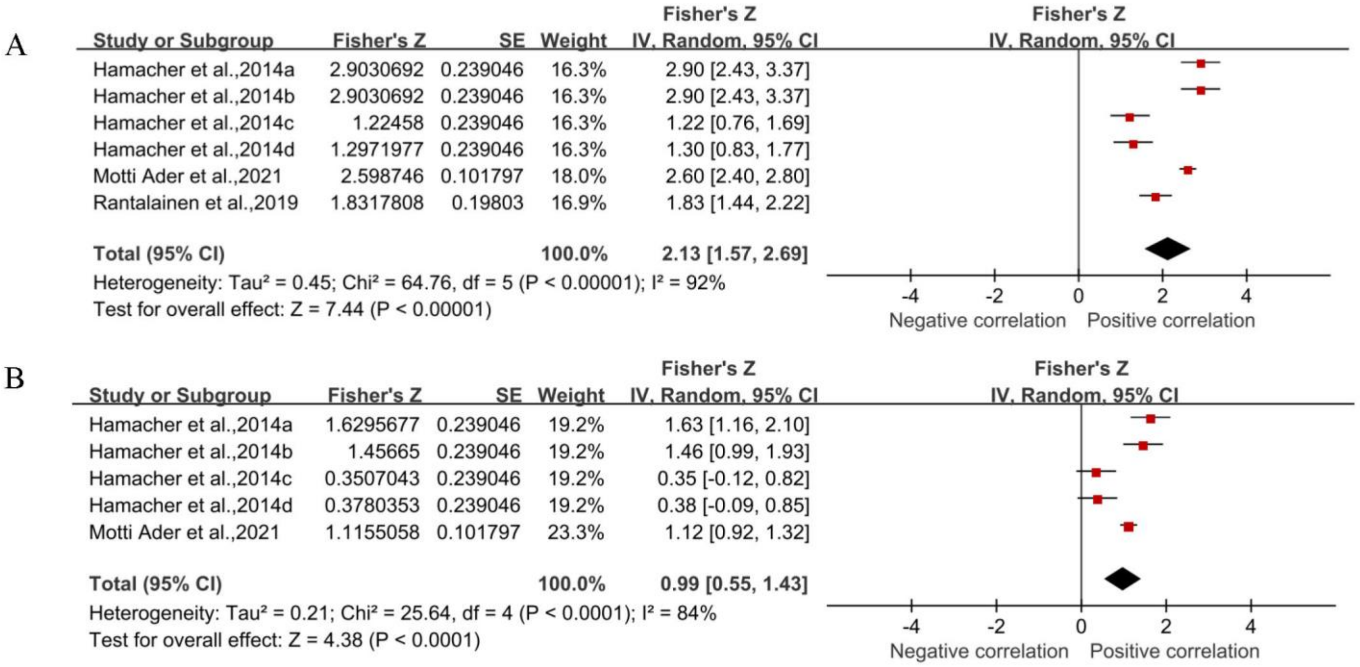

Figure14. Forest plot on the reliability of (A) stance time and (B) stance time variability which represented by intraclass correlation coefficients (ICCs) measured by IMU. Hamacher et al.,2014a(Intra-day, left),b(Intra-day, right),c(Inter-day, left),d(Inter-day, right). *SE* standard error, *IV* inverse variance, *CI* confidence interval.

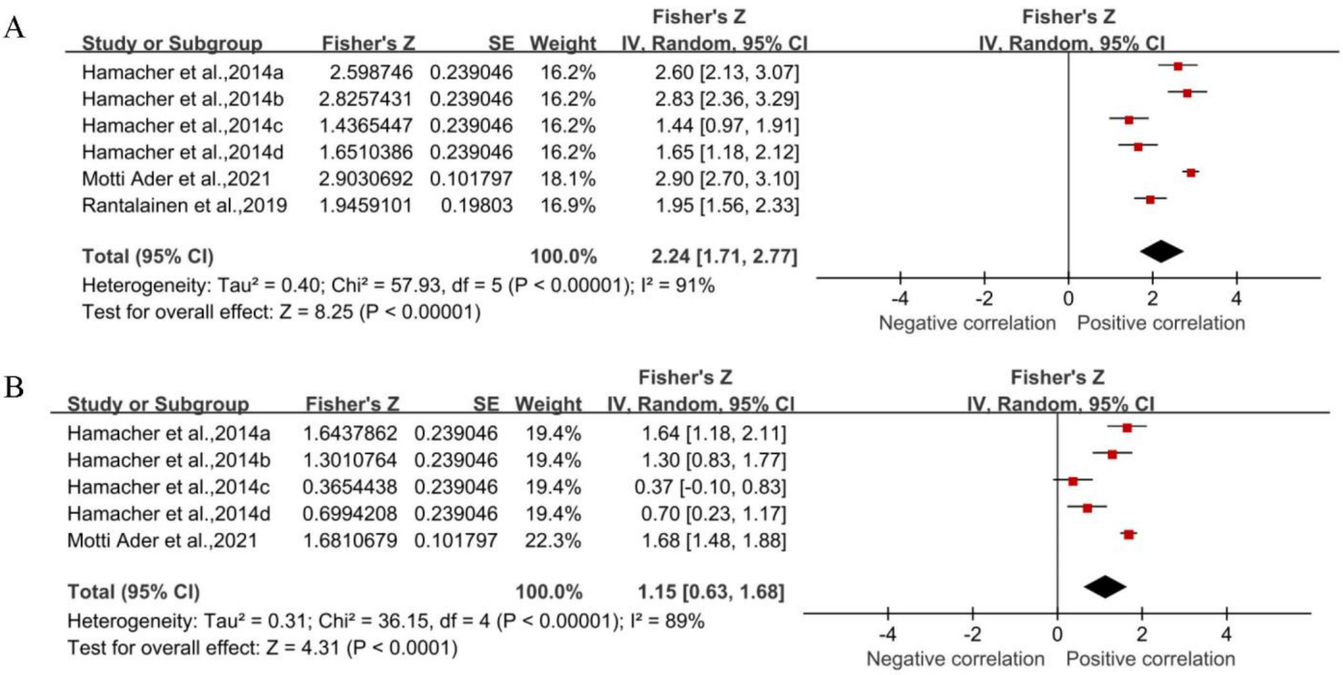

Figure15. Forest plot on the reliability of (A) swing time and (B) swing time variability which represented by intraclass correlation coefficients (ICCs) measured by IMUs. Hamacher et al.,2014a(Intra-day, left),b(Intra-day, right),c(Inter-day, left),d(Inter-day, right). *SE* standard error, *IV* inverse variance, *CI* confidence interval.

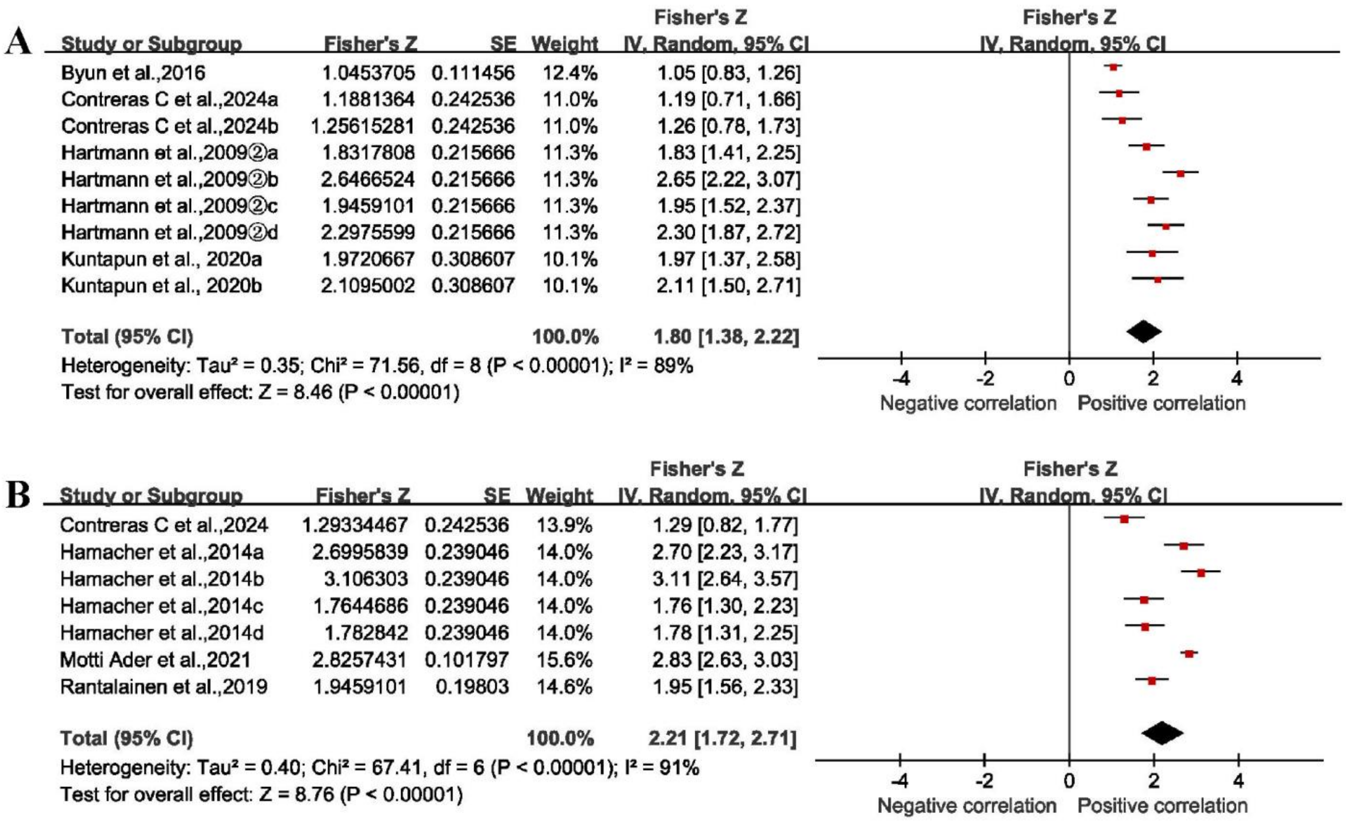

Figure16. Forest plot on the reliability of step length (A) and stride length (B) which represented by intraclass correlation coefficients (ICCs) measured by IMUs. Contreras C et al.,2024a(left), b(right); Hartmann et al.,2009②a(Intra-rater, Gym floor),b(Intra-rater, Soft foam rubber),c(Inter-rater, Gym floor),d(Inter-rater, Soft foam rubber). Kuntapun et al., 2020a(back), b(shoulder bag). Hamacher et al.,2014a(Intra-day, left),b(Intra-day, right),c(Inter-day, left),d(Inter-day, right). *SE* standard error, *IV* inverse variance, *CI* confidence interval.

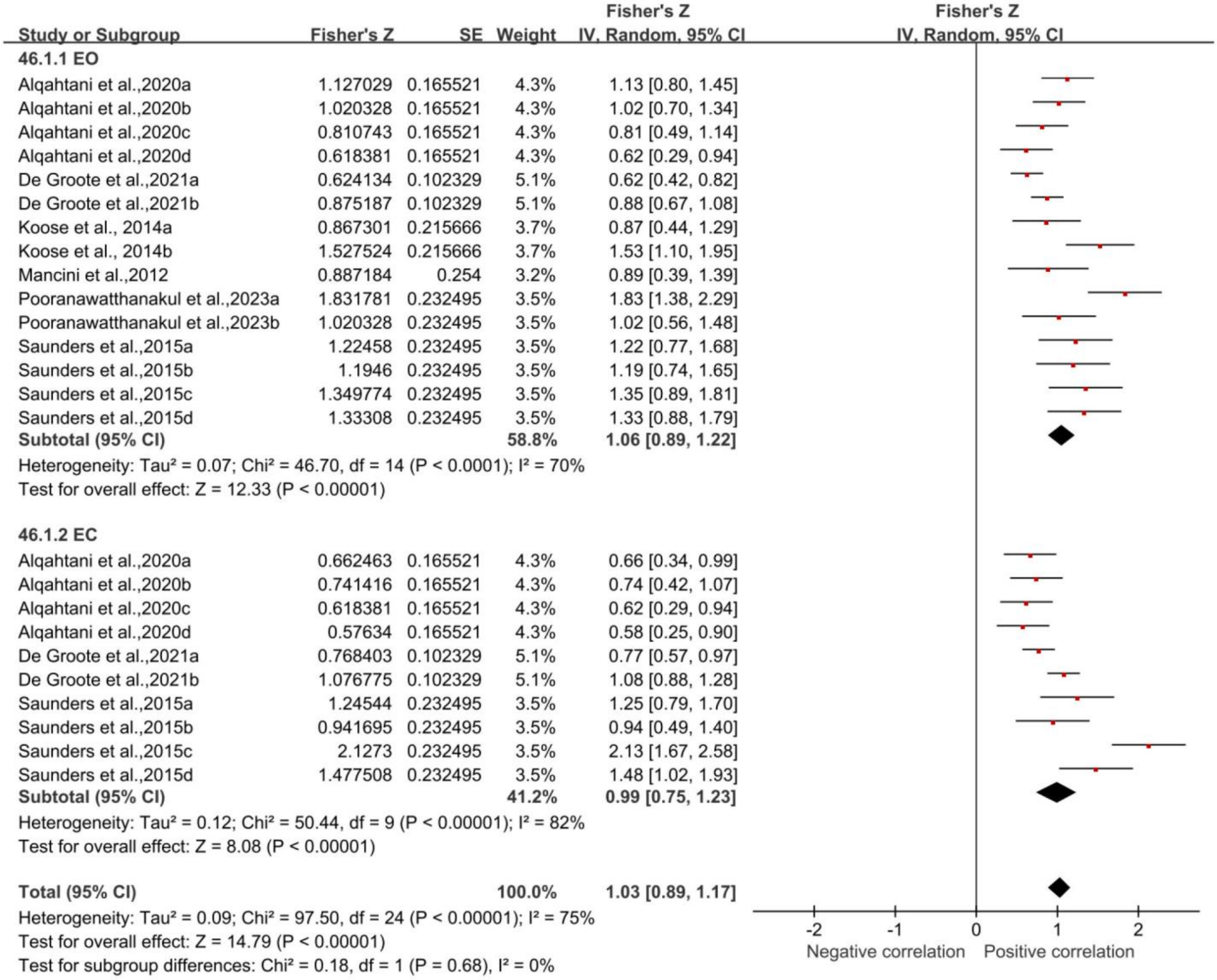

Figure17. Subgroup analysis on the reliability of RMS acceleration during double leg stance which represented by intraclass correlation coefficients (ICCs) measured by IMUs. Alqahtani et al.,2020 a(firm_AP), b(unfirm_AP), c(firm_ML), d(unfirm_ML). De Groote et al.,2021a(AP),b(ML). Koose et al.,2014 a(AP), b(ML). Pooranawatthanakul et al.,2023a(firm_SUM),b（unfirm_sum). Saunders et al.,2015 a(firm_AP), b(unfirm_AP),c(firm_ML), d(unfirm_ML). *EO* eyes open, *EC* eyes closed, *SE* standard error, *IV* inverse variance, *CI* confidence interval.

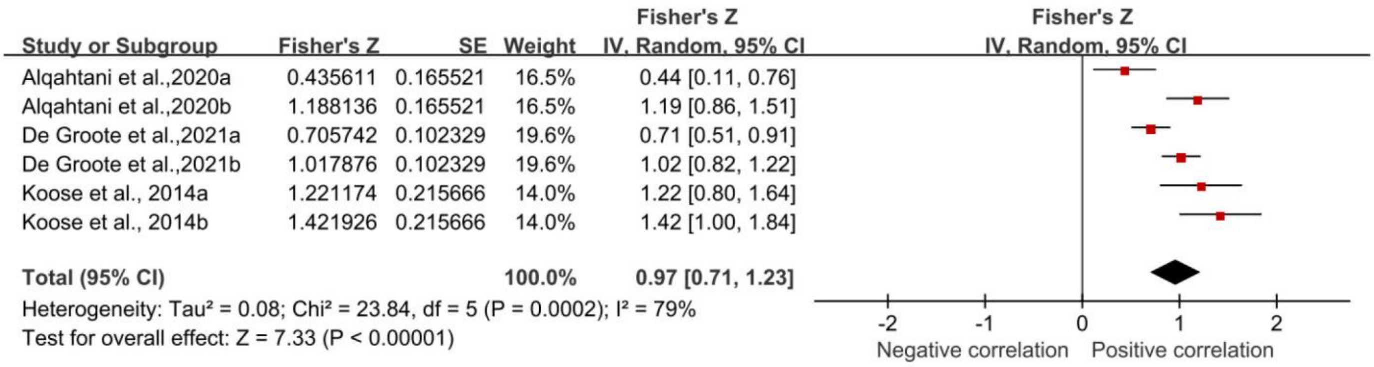

Figure18. Forest plot on the reliability of RMS acceleration during semi-tandem stance which represented by intraclass correlation coefficients (ICCs) measured by IMUs. Alqahtani et al.,2020 a(AP), b(ML). De Groote et al.,2021a(AP),b(ML) Koose et al.,2014 a(AP), b(ML). *SE* standard error, *IV* inverse variance, *CI* confidence interval.

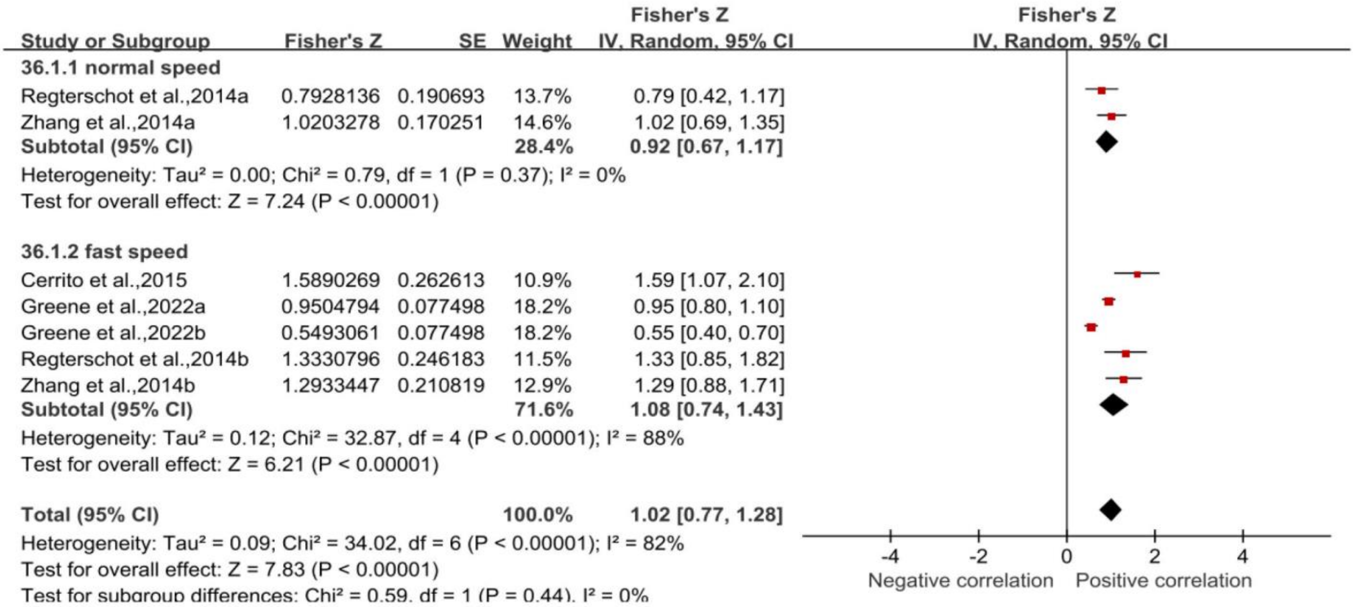

Figure19. Subgroup analysis on the reliability of individual sit to stand duration which represented by intraclass correlation coefficients (ICCs) measured by IMUs. Greene et al.,2022a(Torsor sensor),b(Thigh sensor). Regterschot et al.,2014a(normal speed), b(fast speed). Zhang et al.,2014a(normal speed), b(fast speed). *SE* standard error, *IV* inverse variance, *CI* confidence interval.

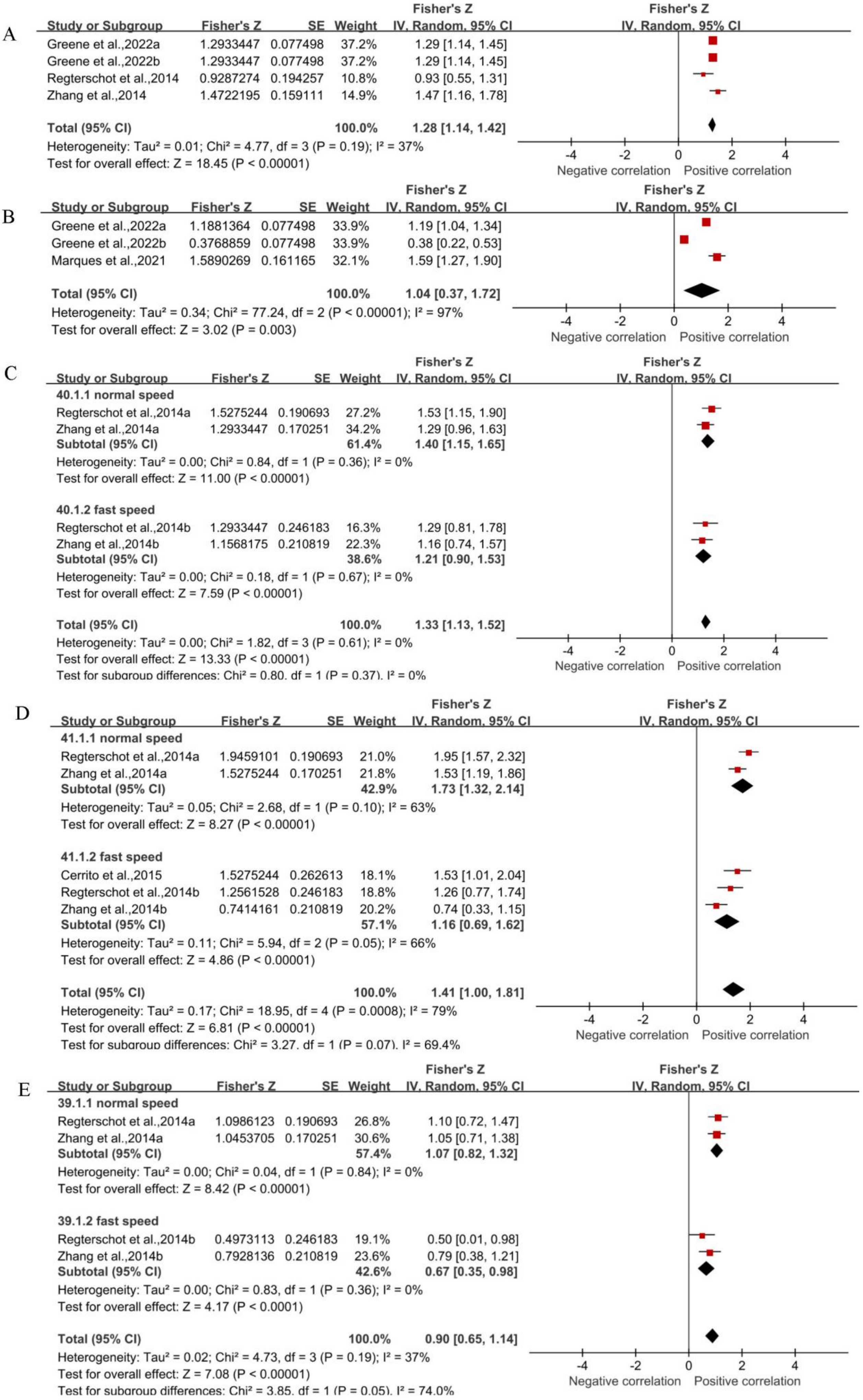
A
Study or Subgroup | Fisher's Z | SE | Weight | Fisher's Z IV, Random, 95% CI
Greene et al.,2022a 1.2933447 0.077498 37.2% 1.29 [1.14, 1.45]
Greene et al.,2022b 1.2933447 0.077498 37.2% 1.29 [1.14, 1.45]
Regterschot et al.,2014 0.9287274 0.194257 10.8% 0.93 [0.55, 1.31]
Zhang et al.,2014 1.4722195 0.159111 14.9% 1.47 [1.16, 1.78]
Total (95% CI) 100.0% 1.28 [1.14, 1.42]
Heterogeneity: Tau² = 0.01; Chi² = 4.77, df = 3 (P = 0.19); I² = 37%
Test for overall effect: Z = 18.45 (P < 0.00001)
Fisher's Z IV, Random, 95% CI
-4 -2 0 2 4
Negative correlation Positive correlation
B
Study or Subgroup | Fisher's Z | SE | Weight | Fisher's Z IV, Random, 95% CI
Greene et al.,2022a 1.1881364 0.077498 33.9% 1.19 [1.04, 1.34]
Greene et al.,2022b 0.3768859 0.077498 33.9% 0.38 [0.22, 0.53]
Marques et al.,2021 1.5890269 0.161165 32.1% 1.59 [1.27, 1.90]
Total (95% CI) 100.0% 1.04 [0.37, 1.72]
Heterogeneity: Tau² = 0.34; Chi² = 77.24, df = 2 (P < 0.00001); I² = 97%
Test for overall effect: Z = 3.02 (P = 0.003)
Fisher's Z IV, Random, 95% CI
-4 -2 0 2 4
Negative correlation Positive correlation
C
Study or Subgroup | Fisher's Z | SE | Weight | Fisher's Z IV, Random, 95% CI
40.1.1 normal speed
Regterschot et al.,2014a 1.5275244 0.190693 27.2% 1.53 [1.15, 1.90]
Zhang et al.,2014a 1.2933447 0.170251 34.2% 1.29 [0.96, 1.63]
Subtotal (95% CI) 61.4% 1.40 [1.15, 1.65]
Heterogeneity: Tau² = 0.00; Chi² = 0.84, df = 1 (P = 0.36); I² = 0%
Test for overall effect: Z = 11.00 (P < 0.00001)
40.1.2 fast speed
Regterschot et al.,2014b 1.2933447 0.246183 16.3% 1.29 [0.81, 1.78]
Zhang et al.,2014b 1.1568175 0.210819 22.3% 1.16 [0.74, 1.57]
Subtotal (95% CI) 38.6% 1.21 [0.90, 1.53]
Heterogeneity: Tau² = 0.00; Chi² = 0.18, df = 1 (P = 0.67); I² = 0%
Test for overall effect: Z = 7.59 (P < 0.00001)
Total (95% CI) 100.0% 1.33 [1.13, 1.52]
Heterogeneity: Tau² = 0.00; Chi² = 1.82, df = 3 (P = 0.61); I² = 0%
Test for overall effect: Z = 13.33 (P < 0.00001)
Test for subgroup differences: Chi² = 0.80, df = 1 (P = 0.37), I² = 0%
Fisher's Z IV, Random, 95% CI
-4 -2 0 2 4
Negative correlation Positive correlation
D
Study or Subgroup | Fisher's Z | SE | Weight | Fisher's Z IV, Random, 95% CI
41.1.1 normal speed
Regterschot et al.,2014a 1.9459101 0.190693 21.0% 1.95 [1.57, 2.32]
Zhang et al.,2014a 1.5275244 0.170251 21.8% 1.53 [1.19, 1.86]
Subtotal (95% CI) 42.9% 1.73 [1.32, 2.14]
Heterogeneity: Tau² = 0.05; Chi² = 2.68, df = 1 (P = 0.10); I² = 63%
Test for overall effect: Z = 8.27 (P < 0.00001)
41.1.2 fast speed
Cerrito et al.,2015 1.5275244 0.262613 18.1% 1.53 [1.01, 2.04]
Regterschot et al.,2014b 1.2561528 0.246183 18.8% 1.26 [0.77, 1.74]
Zhang et al.,2014b 0.7414161 0.210819 20.2% 0.74 [0.33, 1.15]
Subtotal (95% CI) 57.1% 1.16 [0.69, 1.62]
Heterogeneity: Tau² = 0.11; Chi² = 5.94, df = 2 (P = 0.05); I² = 66%
Test for overall effect: Z = 4.86 (P < 0.00001)
Total (95% CI) 100.0% 1.41 [1.00, 1.81]
Heterogeneity: Tau² = 0.17; Chi² = 18.95, df = 4 (P = 0.0008); I² = 79%
Test for overall effect: Z = 6.81 (P < 0.00001)
Test for subgroup differences: Chi² = 3.27, df = 1 (P = 0.07), I² = 69.4%
Fisher's Z IV, Random, 95% CI
-4 -2 0 2 4
Negative correlation Positive correlation
E
Study or Subgroup | Fisher's Z | SE | Weight | Fisher's Z IV, Random, 95% CI
39.1.1 normal speed
Regterschot et al.,2014a 1.0986123 0.190693 26.8% 1.10 [0.72, 1.47]
Zhang et al.,2014a 1.0453705 0.170251 30.6% 1.05 [0.71, 1.38]
Subtotal (95% CI) 57.4% 1.07 [0.82, 1.32]
Heterogeneity: Tau² = 0.00; Chi² = 0.04, df = 1 (P = 0.84); I² = 0%
Test for overall effect: Z = 8.42 (P < 0.00001)
39.1.2 fast speed
Regterschot et al.,2014b 0.4973113 0.246183 19.1% 0.50 [0.01, 0.98]
Zhang et al.,2014b 0.7928136 0.210819 23.6% 0.79 [0.38, 1.21]
Subtotal (95% CI) 42.6% 0.67 [0.35, 0.98]
Heterogeneity: Tau² = 0.00; Chi² = 0.83, df = 1 (P = 0.36); I² = 0%
Test for overall effect: Z = 4.17 (P < 0.0001)
Total (95% CI) 100.0% 0.90 [0.65, 1.14]
Heterogeneity: Tau² = 0.02; Chi² = 4.73, df = 3 (P = 0.19); I² = 37%
Test for overall effect: Z = 7.08 (P < 0.00001)
Test for subgroup differences: Chi² = 3.85, df = 1 (P = 0.05), I² = 74.0%
Fisher's Z IV, Random, 95% CI
-4 -2 0 2 4
Negative correlation Positive correlation

Figure20. Forest plot on the reliability of total sit to stand duration (A), stand-up duration(B), sit to stand maximal velocity(C), sit to stand peak power(D) and sit to stand maximal jerk (E). Greene et al.,2022a (torsor sensor), b (thigh sensor). Regterschot et al.,2014a (normal speed), b (fast speed). Zhang et al.,2014a (normal speed), b (fast speed). *SE* standard error, *IV* inverse variance, *CI* confidence interval.

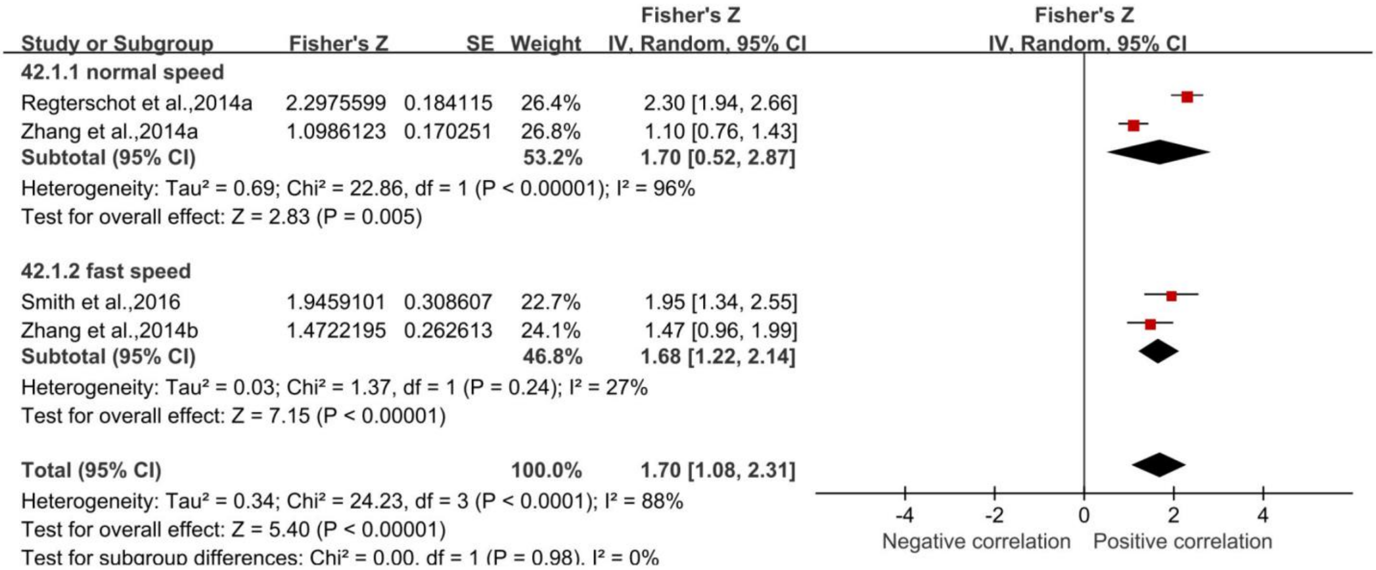

Figure21. Subgroup analysis on the reliability of timed up and go test duration which represented by intraclass correlation coefficients (ICCs) measured by IMUs. Chan et al.,2016a(left), b(right). Regterschot et al.,2014a(normal speed), b(fast speed). Zhang et al.,2014a(normal speed), b(fast speed). *SE* standard error, *IV* inverse variance, *CI* confidence interval.

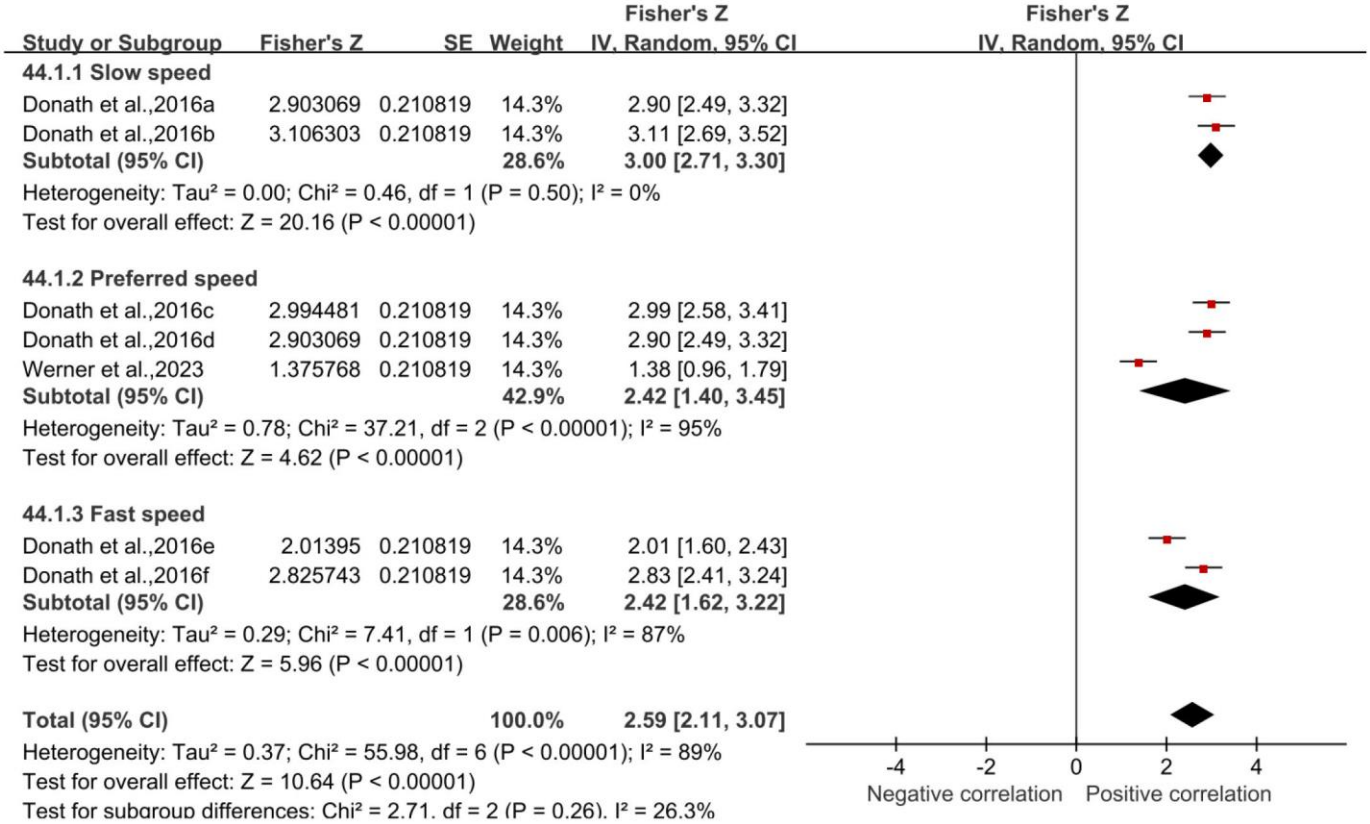

Figure22. Subgroup analysis on the reliability of walking speed during 6 minute walk test which represented by intraclass correlation coefficients (ICCs) measured by IMUs. Donath et al.,2016a(0% slope, slow speed), b(15% slope, slow speed),c(0% slope, normal speed), d(15% slope, normal speed),e(0% slope, fast speed), f(15% slope, fast speed). *SE* standard error, *IV* inverse variance, *CI* confidence interval.

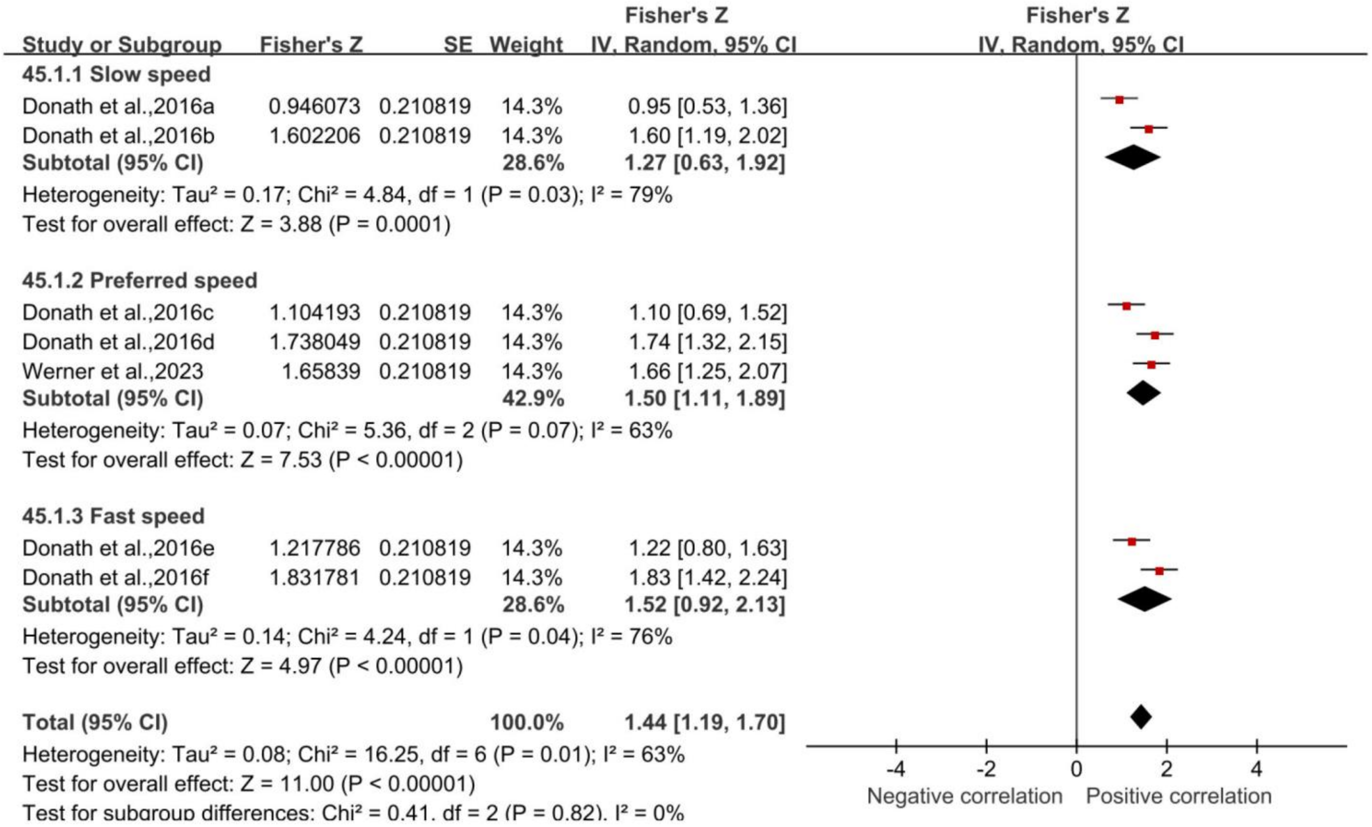

Figure23. Subgroup analysis on the reliability of stride length during 6 minute walk test which represented by intraclass correlation coefficients (ICCs) measured by IMUs. Donath et al.,2016a(0% slope, slow speed), b(15% slope, slow speed),c(0% slope, normal speed), d(15% slope, normal speed),e(0% slope, fast speed), f(15% slope, fast speed). *SE* standard error, *IV* inverse variance, *CI* confidence interval.

Supplementary Table1: Validity assessment for gait outcomes.

	Study	Results					Evidence
		ICC(95%CI)/r	RMSE/SEM	CVs/CV%	Bias (95%CI)	LoA(95%CI)	
Walking speed	Álvarez et al.,2023(1)	ICC= 0.885(0.844-0.916）	-	-			Moderate evidence
	Byun et al.,2016(2)	ICC=0.91(0.86-0.94)<br>r=0.912	-	-		0.02±0.02 m/s	
	Byun et al.,2019(3)	Model 1<br>ICC=0.937 (0.918,0.952)<br>Model 2<br>ICC=0.446 (-0.058, 0.783)<br>Model 3<br>ICC=0.585 (-0.052,0.865)<br>Model 4<br>ICC=0.893 (0.861, 0.918)<br>Model 5<br>ICC=0.920 (0.895,0.939)	Model 1<br>RMSE:6.81 cm/s<br>Model 2<br>RMSE:25.99 cm/s<br>Model 3<br>RMSE:22.07 cm/s<br>Model 4<br>RMSE:8.63 cm/s<br>Model 5<br>RMSE:7.59 cm/s	-	-		
	Contreras C et al.,2024(4)	r=0.87			0.12(0.07,0.16)	±0.19(0.11,0.26)	
	Ensink et al.,2023(5)	-	-	-	-	Treadmill walking:<br>Regular:<br>−0.03±0.04 m/s<br>Irregular:<br>−0.03±0.04 m/s<br>Overground walking:<br>−0.08±0.07 m/s	
	Hartmann et al.,2009①(6)	Slow：<br>ICC=1.00(1.00,1.00)<br>Preferred:<br>ICC=0.99 (0.95,1.00)<br>Fast:<br>ICC=1.00 (0.99,1.00)	-	-	-	-	
	Maggio et al.,2016(7)	Men: r=0.62<br>Women: r=0.73	-	-	-	-	
	Matikainen-Tervola E et al.,2024(8)	ICC : 0.41(0.08, 0.72)	0.279	-	0.207(-0.170,0.583)	-	

	Kuntapun et al., 2020(9)	Smartphone-Body r=0.96 Smartphone-Bag r=0.96	-	-	Smartphone-Body 0.13(-0.03, 0.29) m/s Smartphone-Bag 0.09(-0.06, 0.25) m/s	-	
	Rantalainen et al.,2019(10)	ICC：0.98（0.96,0.99) r^2=0.98	-	1.9%	0.02(0.01,0.03) m/s	-	
	Rogan et al.,2017(11)	Normal walking ICC=0.36(-0.07,0.68) Normal walking with counting ICC=0.36(-0.07,0.67) Fast walking ICC=0.34(-0.61,0.52) Fast walking with counting ICC=0.96(0.96,0.99)	-	-	-	-	
	Rudisch et al.,2021(12)	IMUs vs GAITRite ICC=0.997 IMUs vs Optogait ICC=0.994 IMUs vs Zebris ICC=0.967	-	-	-	-	
Walking speed SD	Rantalainen et al.,2019(10)	ICC=-0.05(-0.41,0.33) r^2=0.07		35.6%	-0.0238 (-0.0342, -0.0133) m/s	-	Very limited evidence
Steps count	Magistro et al.,2018(8)	-	-	-	Slow speed: -0.8(-3.2, 1.7) Preferred speed: -0.8(-3.5, 1.9) Fast speed: -2.8(-8.5, 2.8)	-	Moderate evidence
	Rüdiger et al.,2019(13)	r_c=0.802	-	-	-	-	
	Foster et al.,2022(14)	Garmin vs video 0.44m/s: ICC=0.67(0.19,0.85) 0.89m/s: ICC=0.98(0.96,0.99) 1.33m/s: ICC=0.99	-	-	Garmin vs video 0.44m/s: -57.3(-264.9,150.3) 0.89m/s: -3.4(-27.1, 20.3) 1.33m/s:	-	

		(0.98,1.00)<br>1.67m/s: ICC=0.99 (0.97,0.99)<br>ActivPAL3 vs video<br>0.44m/s: ICC=0.86(0.66,0.93)<br>0.89m/s: ICC=0.98(0.80,1.00)<br>1.33m/s: ICC=0.99 (0.71,1.00)<br>1.67m/s: ICC=0.99 (0.25,1.00)			-2.0(-19.3, 15.3)<br>1.67m/s:<br>-0.6(-15.2, 16.4)<br>ActivPAL3 vs video<br>0.44m/s:<br>-26.2(-144.0, 91.6)<br>0.89m/s:<br>-5.8(-19.9, 8.3)<br>1.33m/s:<br>-4.6(-13.8, 4.5)<br>1.67m/s:<br>-7.1(-20.8, 6.6)		
	Maganja et al.,2020(15)	-	-		Misfit Shine:<br>-6.9(-122.1,108.3)<br>Fitbit One:<br>13.4(-40.5, -13.5)<br>Jawbone UP2:<br>-31.2(-102.2, 39.8)<br>Garmin vívofit 2:<br>-36.3(-170.5, 97.9)<br>Fitbit Charge:<br>-53.4(-194.1, 87.4)	-	
	Phillips et al.,2015(16)	Fitbit at waist:<br>CCC=-0.341(-0.626,0.026)<br>GT1M at ankle:<br>CCC=0.205(0.059,0.344)<br>GT1M at waist:<br>CCC=-0.021(-0.146,0.106)<br>GT1M at wrist:<br>CCC=-0.011(-0.119,0.097)	-	-	Fitbit at waist:<br>-8.3(-15.5, -2.0)<br>GT1M at ankle:<br>-12.2(-25.5, -5.0)<br>GT1M at waist:<br>-61.1(-73.5, -51.9)<br>GT1M at wrist:<br>-70.6(-76.4, -61.0)	-	
Cadence	Byun et al.,2016 (2)	ICC=0.73 (0.61, 0.82)<br>r=0.745	-		3.41±1.42 steps/min	-	Moderate evidence
	Contreras C et al.,2024(4)	r=0.99	-	-	-0.97(-1.54, -0.41)	±2.42(1.43, 3.40)	
	Kuntapun et al., 2020(9)	Smartphone-Body<br>r=1.00<br>Smartphone-Bag<br>r=1.00	-	-	Smartphone-Body<br>-0.2(-1.7, 1.2) steps/min<br>Smartphone-Bag	-	

					-0.9(-2.8, 1.0) steps/min		
	Rogan et al.,2017(11)	Normal walking ICC=0.73(0.72,0.91)<br>Normal walking with counting ICC=0.03(7.20,0.91)<br>Fast walking ICC=0.20(-0.30,0.61)<br>Fast walking with counting ICC=0.61(0.57,0.87)	-	-	-	-	
	Hartmann et al.,2009①(6)	Slow：<br>ICC=1.00（1.00,1.00）<br>Preferred:<br>ICC=1.00（1.00,1.00）<br>Fast:<br>ICC=1.00（1.00,1.00）	-	-	-	-	
	Rudisch et al.,2021(12)	IMUs vs GAITRite ICC=0.999<br>IMUs vs Optogait ICC=0.997<br>IMUs vs Zebris ICC=0.988	-	-	-	-	
Step time	Byun et al.,2016(2)	ICC=0.75 (0.64, 0.83)<br>r=0.761	-	-	-	-0.01±0.01s	Moderate evidence
	Kuntapun et al., 2020(9)	Smartphone-Body r=1<br>Smartphone-Bag r=1	-		Smartphone-Body 0.6(-5.0, 6.3) ms<br>Smartphone-Bag 5.4(-4.1, 15.0) ms	-	
	Matikainen-Tervola E et al.,2024(17)	ICC : 0.88(0.88, 0.89)	0.023	-	-0.000(-0.044,0.044)	-	
	Rogan et al.,2017(11)	Normal walking<br>Left: ICC=0.25(-0.11,0.69)<br>Right: ICC=0.25(-0.11,0.67)<br>Normal walking with counting<br>Left: ICC=0.25(-0.11,0.69)	-		-	-	

		Right: ICC=0.25(-0.09,0.67)<br>Fast walking<br>Left: ICC=0.22(-0.20,0.65)<br>Right: ICC=0.27(-0.73,0.69)<br>Fast walking with counting<br>Left: ICC=0.12(-0.46,0.57)<br>Right: ICC=0.15(-1.47,0.28)					
	Digo et al.,2023(18)	**Slow:**<br>Trunk: r=0.96<br>Shank: r=0.96<br>Ankle: r=0.96<br>**Normal:**<br>Trunk: r=0.98<br>Shank: r=0.85<br>Ankle: r=0.99<br>**Fast:**<br>Trunk: r=0.87<br>Shank: r=0.76<br>Ankle: r=0.88	**Slow:**<br>Trunk: 0.03s(L),0.05s(R)<br>Shank: 0.03s(L),0.07s(R)<br>Ankle: 0.02s(L),0.05s(R)<br>**Normal:**<br>Trunk: 0.02s(L),0.02s(R)<br>Shank: 0.04s(L),0.03s(R)<br>Ankle: 0.02s(L),0.01s(R)<br>**Fast:**<br>Trunk: 0.02s(L),0.05s(R)<br>Shank: 0.04s(L),0.05s(R)<br>Ankle: 0.01s(L),0.05s(R)	-	-	-	
	Hartmann et al.,2009①(6)	Slow：<br>ICC=1.00（1.00,1.00）<br>Preferred:<br>ICC=1.00（0.99,1.00）<br>Fast:<br>ICC=1.00（0.99,1.00）	-	-	-	-	
	Micó-Amigo et	Back: ICC=0.91	-	-	-	11.11±1.42%	

	al.,2016(19)	Heel: ICC=0.91					
Step time variability	Byun et al.,2016 (2)	ICC=0.06 (-0.16, 0.27)<br>r=0.148	-	-	-	-	Limited evidence
	Hartmann et al.,2009①(6)	Slow：<br>ICC=0.98（0.95,0.99）<br>Preferred:<br>ICC=0.94（0.75,0.98）<br>Fast:<br>ICC=0.88（0.72,0.95）	-	-	-	-	
Step time asymmetry	Byun et al.,2016 (2)	ICC=0.13 (-0.09, 0.34)<br>r=0.192	-		6.44±0.69%	-	Very limited evidence
Stride time	Rantalainen et al.,2019(10)	ICC=1.00(1.00,1.00)<br>r^2=1.00	-	0.2%	0(0,0) ms	-	Moderate evidence
	Bäcklund et al.,2020(20)	r^2=0.99	-	-	-0.003(-0.019, 0.013) s	-	
	Digo et al.,2023(18)	**Slow:**<br>Trunk: r=0.99<br>Shank: r=0.99<br>Ankle: r=0.99<br>**Normal:**<br>Trunk: r=0.99<br>Shank: r=0.87<br>Ankle: r=0.99<br>**Fast:**<br>Trunk: r=0.99<br>Shank: r=0.88<br>Ankle: r=0.99	**Slow:**<br>Trunk:<br>0.04s(L),0.04s(R)<br>Shank:<br>0.03s(L),0.03s(R)<br>Ankle:<br>0.03s(L),0.04s(R)<br>**Normal:**<br>Trunk:<br>0.02s(L),0.02s(R)<br>Shank:<br>0.05s(L),0.05s(R)<br>Ankle:<br>0.02s(L),0.02s(R)<br>**Fast:**<br>Trunk:<br>0.02s(L),0.01s(R)<br>Shank:<br>0.04s(L),0.04s(R)<br>Ankle:<br>0.02s(L),0.02s(R)	-	-	-	
	Ensink et	-	-	-	Treadmill walking:	-	

	al.,2023(5)				Regular: −0.00±0.01 s<br>Irregular: 0.00±0.01 s<br>Overground walking: 0.00±0.03 s		
	Matikainen-Tervola E et al.,2024(17)	ICC : 0.98(0.98, 0.98)	0.015	-	0.000(-0.030,0.030)	-	
	Rantalainen et al.,2020	ICC=1.00(1.00,1.00)<br>r^2=1.00	-	Ankle: 0.1%<br>Back: 0.2%	Ankle: 0(0,0)<br>Back: 0(0,0)	-	
	Rudisch et al.,2021(12)	IMUs vs GAITRite<br>ICC=0.999<br>IMUs vs Optogait<br>ICC=0.997<br>IMUs vs Zebris<br>ICC=0.988	-	-	-	-	
Stride time SD	Rantalainen et al.,2019(10)	ICC=0.00(-0.37,0.37)<br>r^2=0.00	-	31.9%	-2.4(-7.1, 2.4)	-	Moderate evidence
	Rantalainen et al.,2020(21)	Ankle:<br>ICC=0.26(-0.12,0.58)<br>r^2=0.22<br>Back:<br>ICC=0.93(0.86,0.96)<br>r^2=0.91	-	Ankle: 30.5%<br>Back:11.6%	Ankle:<br>-6.8 (-10.3, -3.3)<br>Back:<br>-1.8(-2.7, -0.9)	-	
Stride time variability	Rantalainen et al.,2020(21)	Ankle:<br>ICC=0.10(-0.22,0.40)<br>r^2=0.10<br>Back:<br>ICC=0.89(0.80,0.94)<br>r^2=0.86	-	Ankle:30.4%<br>Back:11.5%	Ankle:<br>-0.66 (-1.01, -0.31)<br>Back:<br>-0.18(-0.27, -0.09)	-	Very limited evidence
Stance time	Rantalainen et al.,2019(10)	ICC=0.81(0.64,0.91)<br>r^2=0.68	-	5.1%	-14(-31,4)	-	Moderate
	Digo et al.,2023(18)	**Slow:**<br>Trunk: r=0.97<br>Shank: r=0.92<br>Ankle: r=0.95<br>**Normal:**<br>Trunk: r=0.93	**Slow:**<br>Trunk:<br>0.06s(L),0.05s(R)<br>Shank:<br>0.06s(L),0.06s(R)<br>Ankle:	-	-	-	

		Shank: r=0.78<br>Ankle: r=0.94<br>**Fast:**<br>Trunk: r=0.96<br>Shank: r=0.69<br>Ankle: r=0.94	0.07s(L),0.07s(R)<br>**Normal:**<br>Trunk:<br>0.03s(L),0.03s(R)<br>Shank:<br>0.04s(L),0.05s(R)<br>Ankle:<br>0.05s(L),0.05s(R)<br>**Fast:**<br>Trunk:<br>0.03s(L),0.03s(R)<br>Shank:<br>0.06s(L),0.05s(R)<br>Ankle:<br>0.05s(L),0.05s(R)				
	Matikainen-Tervola E et al.,2024(17)	ICC : 0.44(-0.05, 0.78)	0.094	-	-0.089(-0.150,-0.027)	-	
Stance time SD	Rantalainen et al.,2019(10)	ICC=-0.05(-0.41,0.33)<br>r^2=0.00	-	30.8%	-4.5(-8.4, -0.7) ms	-	Very limited
Swing time	Rantalainen et al.,2019(10)	ICC=0.43(0.07,0.69)<br>r^2=0.24	-	8.1%	17(0,35) ms	-	Moderate evidence
	Digo et al.,2023(18)	**Slow:**<br>Trunk: r=0.91<br>Shank: r=0.74<br>Ankle: r=0.90<br>**Normal:**<br>Trunk: r=0.81<br>Shank: r=0.53<br>Ankle: r=0.87<br>**Fast:**<br>Trunk: r=0.86<br>Shank: r=0.71<br>Ankle: r=0.89	**Slow:**<br>Trunk:<br>0.04s(L),0.04s(R)<br>Shank:<br>0.06s(L),0.05s(R)<br>Ankle:<br>0.08s(L),0.08s(R)<br>**Normal:**<br>Trunk:<br>0.02s(L),0.03s(R)<br>Shank:<br>0.04s(L),0.03s(R)<br>Ankle:<br>0.05s(L),0.06s(R)<br>**Fast:**<br>Trunk:<br>0.02s(L),0.03s(R)	-	-	-	

			Shank: 0.05s(L),0.04s(R) Ankle: 0.05s(L),0.05s(R)				
	Matikainen-Tervola E et al.,2024(17)	ICC : 0.18(-0.08, 0.46)	0.073	-	0.066(0.003,0.128)	-	
Swing time SD	Rantalainen et al.,2019(10)	ICC=-0.25(-0.57,0.13) r^2=0.00	-	56.2%	-10.7(-15.8, -5.6) ms	-	Very limited evidence
Step length	Byun et al.,2016(2)	ICC=0.800(0.70,0.86) r=0.800	-	-	-0.59±0.87cm	-	Moderate evidence
	Contreras C et al.,2024(4)	Left: r=0.85 Right: r=0.85	-	-	Left: 0.06(0.04,0.08) Right: 0.09(0.07,0.11)	Left: ±0.09(0.05,0.12) Right: ±0.09(0.05,0.12)	
	Hartmann et al.,2009①(6)	Slow: ICC=1.00(0.99,1.00) Preferred: ICC=0.99(0.98,1.00) Fast: ICC=0.99(0.98,1.00)	-	-	-	-	
	Kuntapun et al., 2020(9)	Smartphone-Body r=0.92 Smartphone-Bag r=0.91	-	-	Smartphone-Body 6.9(-1.3,15.0) cm Smartphone-Bag 5.6(-2.3, 13.5) cm	-	
	Matikainen-Tervola E et al.,2024(17)	ICC : 0.29(-0.02, 0.51)	14.820	-	10.266(-10.684,31.215)	-	
	Rogan et al.,2017(11)	Normal walking Left: ICC=0.53(0.46,0.84) Right: ICC=0.45(0.33,0.79) Normal walking with counting Left: ICC=0.78(0.79,0.93) Right: ICC=0.74(0.74,0.92) Fast walking Left: ICC=0.84(0.85,0.95)	-	-	-	-	

		Right: ICC=0.83(0.83,0.95)<br>Fast walking with counting<br>Left: ICC=0.77(0.78,0.93)<br>Right: ICC=0.82(0.82,0.94)					
	Rudisch et al.,2021(12)	IMUs vs GAITRite ICC=0.988<br>IMUs vs Optogait ICC=0.994<br>IMUs vs Zebris ICC=0.965	-	-	-	-	
Step length variability	Hartmann et al.,2009①(6)	Slow： ICC=0.24(-0.26,0.60)<br>Preferred: ICC=0.33(-0.24,0.68)<br>Fast: ICC=0.33(-0.24,0.69)	-	-	-	-	Very limited evidence
Stride length	Contreras C et al.,2024(4)	r=0.88	-	-	0.15(0.11,0.18)	±0.16(0.09,0.22)	Moderate evidence
	Ensink et al.,2023(5)					Treadmill walking:<br>Regular: −0.03±0.04 m<br>Irregular: −0.03±0.05 m<br>Overground walking: −0.08±0.05 m	
	Rantalainen et al.,2019(10)	ICC=0.96(0.92,0.98)<br>r^2=0.96	-	2.1%	0.03(0.02,0.04)	-	
Stride length SD	Rantalainen et al.,2019(10)	ICC=-0.23(-0.55,0.16)<br>r^2=0.07	-	47.0%	-0.0308(-0.041, -0.0204)	-	Very limited evidence
RMS Accelerations	Cole et al.,2014(22)	Detrending method:<br>AP: ICC=0.84(0.54,0.95)<br>ML: ICC=0.63(0.16,0.88)<br>VT: ICC=0.98(0.94,0.99)	Detrending method:<br>AP: SEM=0.08<br>ML: SEM =0.11<br>VT: SEM =0.04	-	-	-	Very limited evidence

		Quaternion method<br>AP: ICC=0.76(-0.06,0.94)<br>ML: ICC=0.71(0.27,0.91)<br>VT: ICC=0.99(0.95,1.00)	Quaternion method<br>AP: SEM =0.11<br>ML: SEM =0.10<br>VT: SEM =0.04				

ICC intraclass correlation coefficient; r_c concordance correlation; *LoA* limits of agreement; *RMSE* root mean square error; *r* pearson correlation coefficient; r^2 coefficient of determination; *SEM* standard error of measurement; *MD* mean difference; *95%CI* 95% confidence interval; *AP* anterior-posterior; *ML* medio-lateral; *VT* vertical; Shaded represents that outcome have been included in the meta-analysis.

Supplementary Table 2: Validity assessment for static balance outcomes.

	Study	Results					Evidence
		ICC/r (95%CI)	RMSE/SEM	CVs/CV%	Bias (95%CI)	LoA(95%CI)	
Mean acceleration	De Groote et al.,2021(23)	Double leg stance：<br>EO,ML: r=0.608<br>EC,ML:r=0.771<br>EO, AP: r=0.357<br>EC, AP: r=0.563<br>Dual task stance:<br>ML: r=0.750<br>AP: r=0.548<br>Semitandem stance:<br>ML: r=0.595<br>AP: r=0.551	-	-	-	-	Very limited evidence
RMS acceleration	De Groote et al.,2021(23)	Double leg stance：<br>EO,ML: r=0.640<br>EC,ML:r=0.809<br>EO, AP: r=0.395<br>EC, AP: r=0.589<br>Dual task stance:<br>ML: r=0.749<br>AP: r=0.585<br>Semitandem stance:<br>ML: r=0.616<br>AP: r=0.600	-	-	-	-	Limited evidence

	Pooranawatthanakul et al.,2023(24)	Double leg stance：<br>Firm EO: r=0.77<br>Firm EC: r=0.85<br>Foam EO: r=0.81<br>Foam EC: r=0.78<br>Single leg stance:<br>Dominant EO: r=0.91<br>Non-dominant EO: r=0.82<br>Dominant EC: r=0.81<br>Non-dominant EC: r=0.70	-	-	Double leg stance：<br>Firm EO: 0.24(-0.08,0.56)<br>Firm EC: 0.33(-0.22,0.88)<br>Foam EO: 0.23(-0.12,0.59)<br>Foam EC:0.00(-0.84,0.85)<br>Single leg stance:<br>Dominant EO: 0.24(-0.56,1.04)<br>Non-dominant EO: 0.32(-1.05,1.70)<br>Dominant EC: 0.55(-0.94,2.03)<br>Non-dominant EC: 0.28(-1.37,1.93)	-	
P2P	Ozinga et al., 2014(25)	C1-C6<br>ML: r=0.917,0.970,0.968,0.718,0.991,0.847<br>AP: r=0.989,0.986,0.896,0.895,0.994,0.813<br>TR: r=0.702,0.974,0.759,0.738,0.953,0.966	-	-	-	-	Very limited evidence
Length	Ferrari L et al.,2024(26)	Semitandem stance:<br>EO,ML:r=0.50<br>EC,ML:r=0.74<br>EO,AP:r=0.50<br>EC,AP:r=0.82	-	-	-	-	Limited evidence
	Ozinga et al., 2014 (25)	C1-C6<br>ML: r=0.795,0.907,0.981,0.980,0.773,0.776<br>AP: r=0.899,0.923,0.731,0.900,0.761,0.644	-	-	-	-	
RMS COM	Ozinga et al., 2014(25)	C1-C6<br>ML: r=0.952,0.982,0.912,0.732,0.953,0.869	-	-	-	-	Very limited evidence

		AP:<br>r=0.980,0.970,0.971,0.778,0.989,0.865<br>TR:<br>r=0.914,0.958,0.870,0.896,0.934,0.931					
Ellipsoid volume (95 % volume)	Ozinga et al., 2014 (25)	C1-C6<br>r=0.992,0.998,0.992,0.963,0.999,0.963	-	-	-	-	Limited evidence
	Ferrari L et al.,2024(26)	Semitandem stance:<br>EO:r=0.65<br>EC:r=0.89	-	-	-	-	
Total power	Ozinga et al., 2014 (25)	C1-C6<br>r=<br>0.792,0.892,0.861,0.851,0.925,0.923	-	-	-	-	Very limited evidence

ICC intraclass correlation coefficient; *LoA* limits of agreement; *r* pearson correlation coefficient; r^2 coefficient of determination; *MD* mean difference; *95%CI* 95% confidence interval; *P2P* peak-to-peak, which quantifies displacement amplitudes; *RMS COM* root-mean-square distance which quantifies the magnitude of COM displacements; *Ellipsoid volume (95 % volume)* with 95 % of probability, contained the center of the points of sway in 3D; *AP* anterior-posterior; *ML* medial-lateral; *TR* trunk rotation; *EO* eyes open; *EC* eyes closed; *ST* semi-tandem; *DT* dual task; Condition 1 = double-leg stance, eyes open, firm surface; Condition 2 = double-leg stance, eyes closed, firm surface; Condition 3 = tandem stance, eyes open, firm surface; Condition 4 = double-leg stance, eyes open, foam surface; Condition 5 = double-leg stance, eyes closed, foam surface; Condition 6 = tandem stance, eyes open, foam surface. Shaded represents that outcome have been included in the meta-analysis.

Supplementary Table3: Validity assessment for sit to stand outcomes.

	Study	Results					Evidence
		ICC/r (95%CI)	RMSE/SEM	CVs/CV%	Bias (95%CI)	LoA(95%CI)	
Individual STS duration (fast)	Adamowicz et al.,2020(27)	-	-	-	-0.10(-0.61,0.41) s	-	Moderate evidence
	Bochicchio et al.,2023(28)	r=0.99<br>r^2=0.003	-	-	-0.11(-0.64, 0.43) s	-	
	Cerrito et al.,2015(29)	r=0.98	-	-	-0.084(-0.398, 0.230) s	-	
	Marques et al.,2021(30)	r^2=0.96	-	-	-0.06(-0.27,0.14) s	-	
	Song et al.,2022(31)	-	-	-	IMU vs motion system:<br>0.06(-0.00, 0.12) s<br>IMU vs force plate:<br>0.05(-0.04, 0.13) s	-	

Individual STS duration (normal)	Regterschot et al.,2016(32)	-	-	-	Hip sensor:<br>0.10(-0.27, 0.47) s<br>Chest sensor:<br>0.23(-0.29, 0.75) s	-	Moderate evidence
	Song et al.,2022(31)	-	-	-	IMU vs motion system:<br>0.03(-0.08, 0.13) s<br>IMU vs force plate:<br>0.03(-0.05, 0.11) s	-	
Total STS duration	Chan et al.,2016(33)	ICC=0.988(0.976-0.994)	-	-	0.27(-1.22,1.76) s	-	Moderate evidence
	Fudickar et al.,2020(34)	r=0.87	-	-	-0.06(-3.43, 3.32) s	-	
Mean velocity	Bochicchio et al.,2023(28)	r=0.76<br>r^2=0.030	-	-	-0.08(-0.2,0.05) m/s	-	Limited evidence
	Orange et al.,2020(35)	r=0.72(0.47, 0.86)	-	-	1.00(0.42,1.57) m/s	-	
Mean power	Bochicchio et al.,2023(28)	r=0.79<br>r^2=0.000	-	-	-58 (-150, 32) W	-	Limited evidence
	Orange et al.,2020(35)	r=0.61(0.30, 0.80)		-	0.34 (-0.21, 0.89) W/kg		
Peak power	Regterschot et al.,2016(32)	-	-	-	Hip sensor:<br>58.2 (-72.7, 155.3) W<br>Chest sensor:<br>146.4 (-108.7, 465) W	-	Moderate evidence
	Cerrito et al.,2015(29)	r=0.69	-	-	3.609 (-1.549, 8.766) W/kg	-	
Maximal acceleration	Regterschot et al.,2016(32)	-	-	-	Hip sensor:<br>0.01(-0.51, 0.53) m/s^2<br>Chest sensor:<br>1.30(0.13, 2.48) m/s^2	-	Very limited evidence
Maximal jerk	Regterschot et al.,2016(32)	-	-	-	Hip sensor:<br>2.98(-6.08, 5.60) m/s^3<br>Chest sensor:<br>-0.54(-9.094, 20.03) m/s^3	-	Very limited evidence
Maximal velocity	Regterschot et al.,2016(32)	-	-	-	Hip sensor:<br>0.07(-0.10, 0.19) m/s	-	Very limited

					Chest sensor:<br>0.18(-0.13, 0.57) m/s		evidence

ICC intraclass correlation coefficient; *LoA* limits of agreement; *r* pearson correlation coefficient; r^2 coefficient of determination; *MD* mean difference; *95%CI* 95% confidence interval;

Supplementary Table4: Validity assessment for timed up and go test outcomes.

	Study	Results					Evidence
		ICC/r (95%CI)	RMSE/SEM	CVs/CV%	Bias (95%CI)	LoA(95%CI)	
TUGT duration (fast)	Hellmers et al.,2018(36)	IMU vs stopwatch:<br>r=0.97<br>IMU vs automated measurements:<br>r=0.99	-	-	IMU vs stopwatch:<br>0.10(-0.65,0.85)<br>IMU vs automated measurements:<br>-0.99(-1.45, -0.55)	-	Limited evidence
	Chan et al.,2016(33)	ICC=0.946(0.889,0.973)	-	-	0.48(-1.66, 2.63) s	-	
	Fudickar et al.,2020(34)	r=0.78	-	-	-0.65(-2.70,1.40)	-	
Acceleration	Walgaard et al.2016(37)	-	Vertical:0.33±0.16(m/s^2)<br>Sideways:0.23±0.13(m/s^2)<br>Forward:0.34±0.13(m/s^2)	-	-	-	Very limited evidence
Velocity	Walgaard et al.2016(37)	-	Vertical:0.04±0.02(m/s)<br>Sideways:0.02±0.01(m/s)<br>Forward:0.07±0.03(m/s)		-	-	Very limited evidence
Displacement	Walgaard et al.2016(37)	-	Vertical:0.02±0.02(m)<br>Sideways:0.01±0.01(m)<br>Forward:0.04±0.03(m)	-	-	-	Very limited evidence
Rotation	Walgaard et al.2016(37)	-	Vertical:1.33±0.78(°)<br>Sideways:0.55±0.18(°)<br>Forward:1.66±1.25(°)	-	-	-	Very limited evidence
Steps count	Magistro et al.,2018 (8)	-	-	-	Slow speed:<br>-0.8(-3.2, 1.7)<br>Preferred speed:<br>-0.8(-3.5, 1.9)<br>Fast speed:<br>-2.8(-8.5, 2.8)	-	Very limited evidence

ICC intraclass correlation coefficient; *LoA* limits of agreement; *r* pearson correlation coefficient; *MD* mean difference; *95%CI* 95% confidence interval;

Supplementary Table5: Validity assessment for 6 minute walk test outcomes.

	Study	Results					Evidence
		ICC/r (95%CI)	RMSE/SEM	CVs/CV%	Bias (95%CI)	LoA(95%CI)	
Walking speed	Donath et al.,2016(38)	0% slope<br>Slow speed:<br>ICC=0.945(0.868,0.977)<br>Normal speed<br>ICC=0.938(0.858,0.973)<br>Fast speed<br>ICC=0.941(0.861,0.975)<br>15% slope<br>Slow speed:<br>ICC=0.918(0.811,0.965)<br>Normal speed<br>ICC=0.937(0.851,0.973)<br>Fast speed<br>ICC=0.951(0.883,0.980)	-	-	-	-	Very limited evidence
Cadence	Donath et al.,2016(38)	0% slope<br>Slow speed:<br>ICC=1.000(1.000,1.000)<br>Normal speed<br>ICC=1.000(1.000,1.000)<br>Fast speed<br>ICC=0.999(0.999,1.000)<br>15% slope<br>Slow speed:<br>ICC=1.000(1.000,1.000)<br>Normal speed<br>ICC=1.000(1.000,1.000)<br>Fast speed<br>ICC=1.000(1.000,1.000)	-	-	-	-	Very limited evidence
Stride time	Kobsar et al.,2014(39)	ICC=1.00(-0.98, 0.98)	-	-	-	-	Moderate evidence
	Donath et al.,2016(38)	0% slope<br>Slow speed:<br>ICC=1.000(1.000,1.000)<br>Normal speed	-	-	-	-	

		ICC=1.000(0.999,1.000)<br>Fast speed<br>ICC=0.999(0.997,0.999)<br>15% slope<br>Slow speed:<br>ICC=1.000(1.000,1.000)<br>Normal speed<br>ICC=1.000(1.000,1.000)<br>Fast speed<br>ICC=1.000(0.999,1.000)					
Stride length	Donath et al.,2016(38)	0% slope<br>Slow speed:<br>ICC=0.728(0.358,0.884)<br>Normal speed<br>ICC=0.880(0.724,0.948)<br>Fast speed<br>ICC=0.791(0.516,0.909)<br>15% slope<br>Slow speed:<br>ICC=0.789(0.512,0.909)<br>Normal speed<br>ICC=0.812(0.557,0.920)<br>Fast speed<br>ICC=0.892(0.739,0.955)	-	-	-	-	Very limited evidence
Steps count	Burton et al., 2018(40)	Fitbit Flex:<br>2MWT (1): ICC=0.77(0.57,0.88)<br>2MWT (2): ICC=0.76(0.53,0.88)<br>Fitbit Charge HR:<br>2MWT (1): ICC=0.95(0.92,0.97)<br>2MWT (2): ICC=0.90(0.83,0.95)	-	-	-	-	Very limited evidence

ICC intraclass correlation coefficient; *LoA* limits of agreement; *r* Pearson correlation coefficient; *MD* mean difference; *95%CI* 95% confidence interval;

Supplementary Table 6: Reliability assessment for gait outcomes.

	Study	Results					Evidence
		ICC/r (95%CI)	RMSE/SEM	CVs/CV%	Bias (95%CI)	LoA(95%CI)	
Walking speed	Bautmans et al.,2019(41)	Intra-observer:<br>single walk:<br>ICC=0.96(0.93,0.98) old fall risk<br>ICC=0.95(0.90,0.97) old<br>Mean of two walks<br>ICC=0.99(0.98,0.99) old fall risk<br>ICC=0.99(0.97,0.99) old<br>Inter-observer:<br>single walk:<br>ICC=0.95(0.90,0.97) old fall risk<br>ICC=0.92(0.85,0.95) old<br>Mean of two walks<br>ICC=0.98(0.97,0.99) old fall risk<br>ICC=0.97(0.95,0.99) old	Intra-observer:<br>single walk:<br>SEM=0.06 old fall risk<br>SEM=0.05 old<br>Mean of two walks<br>SEM=0.04 old fall risk<br>SEM=0.03 old<br>Inter-observer:<br>single walk:<br>SEM=0.07 old fall risk<br>SEM=0.06 old<br>Mean of two walks<br>SEM=0.05 old fall risk<br>SEM=0.04 old	-	Intra-observer:<br>single walk:<br>-0.06(-0.09, 0.04)<br>old fall risk<br>0.95(0.90,0.97)<br>Mean of two walks<br>-0.05(-0.07, -0.03)<br>old fall risk<br>-0.03(-0.05, -0.02) old<br>Inter-observer:<br>single walk:<br>-0.04(-0.07, -0.01) old fall risk<br>-0.03(-0.06, -0.01) old<br>Mean of two walks<br>-0.03(-0.05, -0.01)<br>old fall risk<br>-0.03(-0.05, -0.01) old	-	Moderate evidence
	Byun et al.,2016 (2)	ICC=0.84 (0.76-0.89)<br>r=0.844	-	-	-	-	
	Contreras C et al.,2024(4)	ICC=0.86(0.69,0.94)	-	-	-	-	
	Kuntapun et al., 2020(9)	Smartphone-Body<br>ICC=0.988<br>Smartphone-Bag<br>ICC=0.973	-	-	-	-	
	Rantalainen et al.,2019(10)	ICC=0.95	-	-	-	-	
	Hamacher et al.,2014(42)	Intra-day:<br>ICC (left/right) =0.988/0.989<br>Inter-day:<br>ICC (left/right) =0.880/0.877	-	-	-	-	
	Hartmann et	Intra-rater	-	Intra-rater	-	Intra-rater	

	al.,2009②(43)	Gym floor:<br>ICC=0.93(0.84,0.97)<br>Gym floor with DT:<br>ICC=0.92(0.80,0.97)<br>Soft foam rubber:<br>ICC=0.98(0.93,0.99)<br>Soft foam rubber with DT:<br>ICC=0.93(0.84,0.97)<br>Inter-rater<br>Gym floor:<br>ICC=0.94(0.87,0.98)<br>Gym floor with DT:<br>ICC=0.97(0.91,0.99)<br>Soft foam rubber:<br>ICC=0.96(0.90,0.99)<br>Soft foam rubber with DT:<br>ICC=0.95(0.87,0.98)		Gym floor: 3.1%<br>Gym floor with DT: 3.7%<br>Soft foam rubber: 1.9%<br>Soft foam rubber with DT:3.4%<br>Inter-rater<br>Gym floor: 2.7%<br>Gym floor with DT:2.4%<br>Soft foam rubber:2.0%<br>Soft foam rubber with DT:3.7%		±0.12<br>±0.13<br>±0.07<br>±0.13<br>Inter-rater<br>±0.10<br>±0.09<br>±0.08<br>±0.13	
	Motti Ader et al.,2021(44)	ICC=0.991	-	-	-	-	
Walking speed SD	Rantalainen et al.,2019(10)	ICC=0.36	-	-	0.0019(-0.0097,0.0134)	-	Very limited evidence
walking speed variability	Motti Ader et al.,2021(44)	ICC=0.920	-	-	-	-	Very limited evidence
walking speed asymmetry	Motti Ader et al.,2021(44)	ICC=0.989	-	-	-	-	Very limited evidence
Steps count	Magistro et al.,2018(8)	Right:<br>Normal speed:<br>ICC= 0.980 (0.96-0.99)<br>Slow speed:<br>ICC=0.960 (0.92-0.98)	-	-	-	-	Moderate evidence

		Fast speed:<br>ICC= 0.949 (0.89-0.98)<br>Left:<br>Normal speed:<br>ICC= 0.975 (0.95-0.99)<br>Slow speed:<br>ICC=0.938 (0.87-0.97)<br>Fast speed:<br>ICC=0.961 (0.92-0.98)					
	Maganja et al.,2020(15)	-	Misfit Shine:<br>SEM(%)= 18.3%<br>Fitbit One:<br>SEM(%)= 1.0%<br>Jawbone UP2:<br>SEM(%)= 23.5%<br>Garmin vívofit 2:<br>SEM(%)=7.3%<br>Fitbit Charge:<br>SEM(%)= 18.0%	-	-	-	
	Byun et al.,2016 (2)	ICC=0.76 (0.65, 0.84)<br>r=0.759	-	-	-	-	
	Contreras C et al.,2024(4)	ICC=0.99(0.99,0.99)	-	-	-	-	
	Kuntapun et al., 2020(9)	Smartphone-Body<br>ICC=0.970<br>Smartphone-Bag<br>ICC=0.928	-	-	-	-	
Cadence	Hartmann et al.,2009②(43)	Intra-rater<br>Gym floor:<br>ICC=0.91(0.79,0.96)<br>Gym floor with DT:<br>ICC=0.86(0.69,0.94)<br>Soft foam rubber:<br>ICC=0.95(0.86,0.98)<br>Soft foam rubber with DT:<br>ICC=0.92(0.82,0.97)<br>Inter-rater<br>Gym floor:	-	Intra-rater<br>Gym floor: 2.0%<br>Gym floor with DT: 2.8%<br>Soft foam rubber: 1.6%<br>Soft foam rubber with DT:2.2%	-	Intra-rater<br>±6.26 steps/min<br>±8.50 steps/min<br>±4.96 steps/min<br>±6.65 steps/min<br>Inter-rater<br>±5.72 steps/min<br>±6.91 steps/min<br>±4.58 steps/min<br>±7.73 steps/min	Moderate evidence

		ICC=0.92(0.81,0.96)<br>Gym floor with DT:<br>ICC=0.92(0.82,0.97)<br>Soft foam rubber:<br>ICC=0.94(0.86,0.98)<br>Soft foam rubber with DT:<br>ICC=0.92(0.80,0.96)		Inter-rater<br>Gym floor:1.8%<br>Gym floor with DT:2.3%<br>Soft foam rubber:1.4%<br>Soft foam rubber with DT:2.6%			
Step time	Byun et al.,2016(2)	ICC=0.78 (0.67, 0.85)<br>r=0.779	-	-	-	-	Moderate evidence
	Kuntapun et al., 2020(9)	Smartphone-Body<br>ICC=0.964<br>Smartphone-Bag<br>ICC=0.962	-	-	-	-	
	Hartmann et al.,2009②(43)	Intra-rater<br>Gym floor:<br>ICC=0.91(0.78,0.96)<br>Gym floor with DT:<br>ICC=0.86(0.68,0.94)<br>Soft foam rubber:<br>ICC=0.95(0.86,0.98)<br>Soft foam rubber with DT:<br>ICC=0.92(0.81,0.97)<br>Inter-rater<br>Gym floor:<br>ICC=0.91(0.81,0.96)<br>Gym floor with DT:<br>ICC=0.91(0.80,0.96)<br>Soft foam rubber:<br>ICC=0.95(0.87,0.98)<br>Soft foam rubber with DT:<br>ICC=0.90(0.77,0.96)	-	Intra-rater<br>Gym floor: 2.0%<br>Gym floor with DT: 2.8%<br>Soft foam rubber: 1.6%<br>Soft foam rubber with DT:2.3%<br>Inter-rater<br>Gym floor:1.9%<br>Gym floor with DT:2.4%<br>Soft foam rubber:1.4%<br>Soft foam rubber with	-	Intra-rater<br>±0.03<br>±0.04<br>±0.02<br>±0.03<br>Inter-rater<br>±0.03<br>±0.04<br>±0.02<br>±0.04	

				DT:2.9%			
	Motti Ader et al.,2021(44)	ICC=0.980	-	-	-	-	
Step time variability	Byun et al.,2016 (2)	ICC=0.78 (0.67, 0.85)<br>r=0.766	-	-	-	-	Limited evidence
	Hartmann et al.,2009②(43)	Intra-rater<br>Gym floor:<br>ICC=0.78(0.50,0.89)<br>Gym floor with DT:<br>ICC=0.46(0.04,0.75)<br>Soft foam rubber:<br>ICC=0.12(-0.31,0.54)<br>Soft foam rubber with DT:<br>ICC=0.73(0.44,0.89)<br>Inter-rater<br>Gym floor:<br>ICC=0.59(0.23,0.81)<br>Gym floor with DT:<br>ICC=0.72(0.44,0.87)<br>Soft foam rubber:<br>ICC=0.80(0.54,0.92)<br>Soft foam rubber with DT:<br>ICC=0.80(0.56,0.91)	-	Intra-rater<br>Gym floor: 13.9%<br>Gym floor with DT: 28.4%<br>Soft foam rubber: 31.8%<br>Soft foam rubber with DT:19.8%<br>Inter-rater<br>Gym floor:17.9%<br>Gym floor with DT:21.4%<br>Soft foam rubber:16.9%<br>Soft foam rubber with DT:17.9%	-	Intra-rater<br>±0.89<br>±2.57<br>±2.43<br>±1.77<br>Inter-rater<br>±1.16<br>±1.93<br>±1.35<br>±1.60	
	Motti Ader et al.,2021(44)	ICC=0.530	-	-	-	-	
Step time asymmetry	Byun et al.,2016 (2)	ICC=0.52 (0.34, 0.66)<br>r=0.523	-	-	-	-	Limited evidence
	Bautmans et al.,2019(41)	Intra-observer:<br>single walk:<br>ICC=0.49(0.21,0.69) old fall risk<br>ICC=0.61(0.37,0.77) old	Intra-observer:<br>single walk:<br>SEM=4.08 old fall risk<br>SEM=2.49 old	-	Intra-observer:<br>single walk:<br>1.48(-0.37, 3.32)<br>old fall risk<br>-0.14(-1.26,0.97) old	-	

		Mean of two walks<br>ICC=0.89(0.80,0.94) old fall risk<br>ICC=0.81(0.64,0.90) old<br>Inter-observer:<br>single walk:<br>ICC=0.54(0.28,0.73) old fall risk<br>ICC=0.80(0.65,0.89) old<br>Mean of two walks<br>ICC=0.85(0.72,0.92) old fall risk<br>ICC=0.78(0.59,0.88) old	Mean of two walks<br>SEM=2.16 old fall risk<br>SEM=2.43 old<br>Inter-observer:<br>single walk:<br>SEM=3.78 old fall risk<br>SEM=1.80 old<br>Mean of two walks<br>SEM=2.58 old fall risk<br>SEM=2.50 old		Mean of two walks<br>0.72(-0.26,1.69) old fall risk<br>0.34(-0.75, 1.42) old<br>Inter-observer:<br>single walk:<br>1.00(-0.73,2.74)<br>old fall risk<br>-0.12(-0.93,0.68) old<br>Mean of two walks<br>0.20(-0.99,1.38)<br>old fall risk<br>0.10(-0.10,1.22) old		
	Motti Ader et al.,2021(44)	ICC=0.131	-	-	-	-	
Stride time	Rantalainen et al.,2019(10)	ICC=0.96	-	-	10(0,20)		Moderate evidence
	Rantalainen et al.,2020(21)	Ankle:<br>ICC=0.95(0.90,0.98)<br>waist:<br>ICC=0.95(0.91,0.98)	-	-	Ankle:<br>10(0,20)<br>Waist:<br>10(0,20)	-	
	Motti Ader et al.,2021(44)	ICC=0.992	-	-	-	-	
Stride time SD	Rantalainen et al.,2019(10)	ICC=0.38	-	-	1.3(-2.7,5.2)	-	Limited evidence
	Rantalainen et al.,2020(21)	Ankle:<br>ICC=0.47(0.18,0.68)<br>Back:<br>ICC=0.66(0.43,0.81)	-	-	Ankle: 1.5(-2.3, 5.4)<br>Back: -1.6(-4.6, 1.3)	-	
Stride time variability	Rantalainen et al.,2020(21)	Ankle:<br>ICC=0.41(0.12,0.64)<br>Back:<br>ICC=0.58(0.32,0.76)	-	-	Ankle: 0.13(-0.24, 0.5)<br>Back: -0.18(-0.48, 0.11)	-	Limited evidence
	Hamacher et al.,2014(42)	Intra-day:<br>ICC (left/right) =0.859/0.809<br>Inter-day:	-	-	Intra-day:<br>Left:0.000<br>Right:0.069<br>Inter-day:	-	

		ICC (left/right) =0.542/0.450			Left: - 0.211<br>Right: -0.123		
	Motti Ader et al.,2021(44)	ICC=0.925	-	-	-	-	
Stride time asymmetry	Motti Ader et al.,2021(44)	ICC=0.487	-	-	-	-	Very limited evidence
Stance time	Rantalainen et al.,2019(10)	ICC=0.95	-	-	8(-1, 17)	-	Moderate evidence
	Hamacher et al.,2014(42)	Intra-day:<br>ICC (left/right) =0.994/0.994<br>Inter-day:<br>ICC (left/right) =0.841/0.861	-	-	Intra-day:<br>Left: -0.001<br>Right: -0.001<br>Inter-day:<br>Left: 0.000<br>Right: -0.008	-	
	Motti Ader et al.,2021(44)	ICC=0.989	-	-		-	
Stance time SD	Rantalainen et al.,2019(10)	ICC=0.65	-	-	0.6(-2,3.1)	-	Very limited evidence
Stance time variability	Hamacher et al.,2014(42)	Intra-day:<br>ICC (left/right) =0.926/0.897<br>Inter-day:<br>ICC (left/right) =0.337/0.361	-	-	Intra-day:<br>Left:0.115<br>Right:0.079<br>Inter-day:<br>Left: -0.365<br>Right: -0.281	-	Limited evidence
	Motti Ader et al.,2021(44)	ICC=0.806	-	-	-	-	
Stance time asymmetry	Motti Ader et al.,2021(44)	ICC=0.906	-	-	-	-	Very limited evidence
Swing time	Rantalainen et al.,2019(10)	ICC=0.96	-	-	1(-5,6)	-	Moderate evidence
	Hamacher et al.,2014(42)	Intra-day:<br>ICC (left/right) =0.989/0.993<br>Inter-day:<br>ICC (left/right)	-	-	Intra-day:<br>Left: 0.000<br>Right: -0.001<br>Inter-day:<br>Left: -0.005	-	

		=0.893/0.929			Right: -0.003		
	Motti Ader et al.,2021(44)	ICC=0.994	-	-	-	-	
Swing time SD	Rantalainen et al.,2019(10)	ICC=0.69	-	-	0.6(-2.6, 3.9)	-	Very limited evidence
Swing time variability	Hamacher et al.,2014(42)	Intra-day:<br>ICC (left/right) =0.928/0.862<br>Inter-day:<br>ICC (left/right) =0.350/0.604	-	-	Intra-day:<br>Left:0.000<br>Right:0.067<br>Inter-day:<br>Left: -0.195<br>Right: -0.127	-	Limited evidence
	Motti Ader et al.,2021(44)	ICC=0.933	-	-	-	-	
Swing time asymmetry	Motti Ader et al.,2021(44)	ICC=0.927	-	-	-	-	Very limited evidence
Step length	Byun et al.,2016 (2)	ICC=0.78 (0.67, 0.85)<br>r=0.779	-	-	-	-	Moderate evidence
	Contreras C et al.,2024(4)	Left: ICC=0.83(0.63,0.93)<br>Right: ICC=0.85(0.66,0.93)	-	-	-	-	
	Kuntapun et al., 2020(9)	Smartphone-Body<br>ICC=0.962<br>Smartphone-Bag<br>ICC=0.971	-	-	-	-	
	Hartmann et al.,2009②(43)	Intra-rater<br>Gym floor:<br>ICC=0.95(0.88,0.98)<br>Gym floor with DT:<br>ICC=0.92(0.78,0.96)<br>Soft foam rubber:<br>ICC=0.99(0.96,0.99)<br>Soft foam rubber with DT:<br>ICC=0.95(0.88,0.98)<br>Inter-rater<br>Gym floor:<br>ICC=0.96(0.90,0.98)<br>Gym floor with DT:	-	Intra-rater<br>Gym floor:1.9%<br>Gym floor with DT:2.3%<br>Soft foam rubber:0.9%<br>Soft foam rubber with DT:1.7%<br>Inter-rater<br>Gym	-	Intra-rater<br>±0.04<br>±0.04<br>±0.02<br>±0.03<br>Inter-rater<br>±0.03<br>±0.03<br>±0.02<br>±0.04	

		ICC=0.97(0.94,0.99)<br>Soft foam rubber:<br>ICC=0.98(0.94,0.99)<br>Soft foam rubber with DT:<br>ICC=0.95(0.89,0.98)		floor:1.7%<br>Gym floor with DT:1.6%<br>Soft foam rubber:1.1%<br>Soft foam rubber with DT:2.1%			
Step length variability	Hartmann et al.,2009②(43)	Intra-rater<br>Gym floor:<br>ICC=0.31(-0.10, 0.65)<br>Gym floor with DT:<br>ICC=0.37(-0.06,0.70)<br>Soft foam rubber:<br>ICC=0.21(-0.23,0.60)<br>Soft foam rubber with DT:<br>ICC=0.30(-0.15,0.65)<br>Inter-rater<br>Gym floor:<br>ICC=0.69(0.39,0.86)<br>Gym floor with DT:<br>ICC=0.71(0.41,0.87)<br>Soft foam rubber:<br>ICC=0.68(0.34,0.86)<br>Soft foam rubber with DT:<br>ICC=0.88(0.73,0.95)	-	Intra-rater<br>Gym floor: 30.3%<br>Gym floor with DT: 33.7%<br>Soft foam rubber: 32.3%<br>Soft foam rubber with DT:30.6%<br>Inter-rater<br>Gym floor:21.4%<br>Gym floor with DT:23.2%<br>Soft foam rubber:22.6%<br>Soft foam rubber with DT:12.4%	-	Intra-rater<br>±4.10<br>±5.27<br>±4.71<br>±4.55<br>Inter-rater<br>±2.80<br>±3.48<br>±3.20<br>±1.75	Very limited evidence
Stride length	Contreras C et al.,2024(4)	ICC=0.86(0.69,0.94)	-	-	-	-	Moderate evidence
	Rantalainen et al.,2019(10)	ICC=0.96	-	-	-0.01(-0.02,0.01)	-	

	Hamacher et al.,2014(42)	Intra-day:<br>ICC (left/right) =0.991/0.996<br>Inter-day:<br>ICC (left/right) =0.943/0.945	-	-	Intra-day:<br>Left: -0.001<br>Right: -0.001<br>Inter-day:<br>Left: 0.023<br>Right:0.033	-	
	Motti Ader et al.,2021(44)	ICC=0.993	-	-	-	-	
Stride length SD	Rantalainen et al.,2019(10)	ICC=0.45	-	-	0.0022(-0.0092,0.0135)	-	Very limited evidence
Stride length variability	Motti Ader et al.,2021(44)	ICC=0.925	-	-	-	-	Very limited evidence
Stride length asymmetry	Motti Ader et al.,2021(44)	ICC=0.990	-	-	-	-	Very limited evidence
Step width	Bäcklund et al.,2020(20)	-	-	-	-	two-hour interval: 0.20±0.44<br>one week interval： 0.03±0.39	Very limited evidence

ICC intraclass correlation coefficient; *LoA* limits of agreement; *r* pearson correlation coefficient; *MD* mean difference; *95%CI* 95% confidence interval; *NPL* normalized path length; *DT* dual task; *ICC* intraclass correlation coefficient; *LoA* limits of agreement; *r* pearson correlation coefficient; *MD* mean difference; *95%CI* 95% confidence interval; *AP* anterior-posterior; *ML* medio-lateral; *VT* vertical; Shaded represents that outcome have been included in the meta-analysis.

Supplementary Table 7: Reliability assessment for static balance outcomes.

	Study	Results					Evidence
		ICC/r (95%CI)	RMSE/SEM	CVs/CV%	Bias (95%CI)	LoA(95%CI)	

Mean acceleration	De Groote et al.,2021(23)	EO-ML: ICC=0.698(0.624,0.764)<br>EC-ML: ICC=0.832(0.785,0.872)<br>DT-ML: ICC=0.687(0.610,0.755)<br>ST-ML: ICC=0.759(0.697,0.814)<br>EO-AP: ICC=0.535(0.440,0.626)<br>EC-AP: ICC=0.626(0.541,0.704)<br>DT- AP: ICC=0.557(0.464,0.645)<br>ST-AP: ICC=0.585(0.495,0.669)	-	-	-	-	Very limited evidence
RMS acceleration	De Groote et al.,2021(23)	EO-ML: ICC=0.704(0.631,0.769)<br>EC-ML: ICC=0.792(0.736,0.840)<br>DT-ML: ICC=0.612(0.524,0.693)<br>ST-ML: ICC=0.769(0.708,0.822)<br>EO-AP: ICC=0.554(0.461,0.642)<br>EC-AP: ICC=0.646(0.564,0.721)<br>DT- AP: ICC=0.557(0.463,0.645)<br>ST-AP: ICC=0.608(0.521,0.688)	-	-	-	-	Moderate evidence
	Saunders et al.,2015(45)	Floor:<br>EO-AP: ICC=0.841(0.753,0.929)<br>EO-ML: ICC=0.874(0.804,0.944)<br>EC-AP: ICC=0.847(0.763,0.931)<br>EC-ML: ICC=0.972(0.956,0.988)<br>Mat<br>EO-AP: ICC=0.832(0.729,0.935)<br>EO-ML: ICC=0.870(0.794,0.946)<br>EC-AP: ICC=0.736(0.583,0.889)<br>EC-ML: ICC=0.901(0.839,0.963)	-	Floor:<br>EO-AP: 20.2%<br>EO-ML: 18.0%<br>EC-AP: 29.4%<br>EC-ML: 15.3%<br>Mat<br>EO-AP: 28.6%<br>EO-ML: 17.6%<br>EC-AP: 54.4%<br>EC-ML: 23.6%	-	-	
	Alqahtani et al.,2020(46)	Level:<br>EO-AP: ICC=0.81(0.67,0.89)<br>EO-ML: ICC=0.67(0.47,0.82)<br>EC-AP: ICC=0.58(0.33,0.76)<br>EC-ML: ICC=0.55(0.28,0.74)<br>Foam:<br>EO-AP: ICC=0.77(0.60,0.87)<br>EO-ML: ICC=0.55(0.28,0.73)<br>EC-AP: ICC=0.63(0.40,0.79)<br>EC-ML: ICC=0.52(0.25,0.72)	Level:<br>EO-AP:<br>SEM=1.00<br>EO-ML:<br>SEM=1.15<br>EC-AP:<br>SEM=1.58<br>EC-ML:<br>SEM=1.59<br>Foam:	-	-	-	

		Semi-tandem:<br>AP: ICC=0.41(0.11,0.64)<br>ML: ICC=0.83(0.71,0.91)<br>Tandem:<br>AP: ICC=0.47(0.18,0.68)<br>ML: ICC=0.71(0.51,0.84)	EO-AP:<br>SEM=1.28<br>EO-ML:<br>SEM=2.02<br>EC-AP:<br>SEM=2.41<br>EC-ML:<br>SEM=3.40<br>Semi-tandem:<br>AP: SEM=2.42<br>ML: SEM=0.86<br>Tandem:<br>AP: SEM=3.19<br>ML: SEM=1.43				
	Mancini et al., 2012(47)	Double leg stance：<br>ICC=0.71(0.35,0.89)	-	-	-	-	
	Koose et al., 2014(48)	Parallel stance:<br>EO-AP: 0.70(0.31,0.87)<br>EO-ML:0.91(0.79,0.96)<br>Semi-tandem stance:<br>EO-AP: 0.84(0.62,0.93)<br>EO-ML:0.89(0.73,0.95)	-	-	-	-	
	Pooranawatthanakul et al.,2023(24)	Double leg stance：<br>Firm EO: ICC=0.95(0.89,0.99)<br>Firm EC: ICC =0.79(0.68,0.88)<br>Foam EO: ICC =0.77(0.71,0.83)<br>Foam EC: ICC =0.82 (0.75,0.90)<br>Single leg stance:<br>Dominant EO:<br>ICC =0.83(0.76,0.91)<br>Non-dominant EO:<br>ICC =0.78(0.73,0.89)<br>Dominant EC:<br>ICC =0.76(0.71,0.82)<br>Non-dominant EC:<br>ICC =0.80(0.78,0.86)	Double leg stance：<br>Firm EO:<br>SEM=0.06<br>Firm EC:<br>SEM=0.29<br>Foam EO:<br>SEM=0.19<br>Foam EC:<br>SEM=0.28<br>Single leg stance:<br>Dominant EO:<br>SEM=0.51<br>Non-dominant EO:<br>SEM=0.78	-	-	-	

			Dominant EC: SEM=0.94<br>Non-dominant EC: SEM=0.98				
NPL	Alqahtani et al.,2020(46)	Level:<br>EO-AP: ICC=0.66(0.44,0.81)<br>EO-ML: ICC=0.61(0.37,0.78)<br>EC-AP: ICC=0.66(0.44,0.81)<br>EC-ML: ICC=0.79(0.64,0.88)<br>Foam:<br>EO-AP: ICC=0.71(0.51,0.84)<br>EO-ML: ICC=0.71(0.50,0.83)<br>EC-AP: ICC=0.82(0.67,0.90)<br>EC-ML: ICC=0.81(0.67,0.90)<br>Semi-tandem:<br>AP: ICC=0.35(0.04,0.60)<br>ML: ICC=0.71(0.51,0.84)<br>Tandem:<br>AP: ICC=0.65(0.41,0.80)<br>ML: ICC=0.73(0.54,0.85)	Level:<br>EO-AP: SEM=1.90<br>EO-ML: SEM=2.81<br>EC-AP: SEM=3.18<br>EC-ML: SEM=3.10<br>Foam:<br>EO-AP: SEM=1.87<br>EO-ML: SEM=3.55<br>EC-AP: SEM=2.63<br>EC-ML: SEM=4.97<br>Semi-tandem:<br>AP: SEM=4.13<br>ML: SEM=3.36<br>Tandem:<br>AP: SEM=3.71<br>ML: SEM=3.87	-	-	-	Limited evidence
	Mancini et al. , 2012(47)	ICC=0.89(0.72,0.96)	-	-	-	-	
Jerk	Mancini et al. , 2012(47)	ICC=0.87(0.67,0.95)	-	-	-	-	Very limited evidence
Distance	Mancini et al. , 2012(47)	ICC=0.70(0.34,0.88)	-	-	-	-	Very limited evidence
Displacement	Pedrero-Sánchez et	AP:ICC=0.84	-	-	-	-	Very

	al.,2023(49)	ML:ICC=0.74					limited evidence
Range	Mancini et al. , 2012(47)	ICC=0.74(0.41,0.90)	-	-	-	-	Very limited evidence
Mean velocity	Mancini et al. , 2012(47)	ICC=0.68(0.31,0.87)	-	-	-	-	Very limited evidence
Mean frequency	Mancini et al. , 2012(47)	ICC=0.60(0.17,0.84)	-	-	-	-	Very limited evidence
Area	Mancini et al. , 2012(47)	ICC=0.76(0.45,0.91)	-	-	-	-	Limited evidence
	Koose et al., 2014(48)	Parallel stance:<br>EO: ICC=0.80(0.53,0.91)<br>Semi-tandem stance:<br>EO: ICC=0.94(0.86,0.98)	-	-	-	-	
average Theta score	Peller et al.2022(50)	ICC=0.84	-	-	-	-	Very limited evidence

ICC intraclass correlation coefficient; *LoA* limits of agreement; *r* pearson correlation coefficient; *MD* mean difference; *95%CI* 95% confidence interval; *NPL* normalized path length; *TR* trunk rotation; *EO* eyes open; *EC* eyes closed; *ST* semi-tandem; *DT* dual task; *ICC* intraclass correlation coefficient; *LoA* limits of agreement; *r* pearson correlation coefficient; *MD* mean difference; *95%CI* 95% confidence interval; *AP* anterior-posterior; *ML* medio-lateral; *VT* vertical; Shaded represents that outcome have been included in the meta-analysis.

Supplementary Table8: Reliability assessment for sit to stand outcomes.

	Study	Results					Evidence
		ICC/r (95%CI)	RMSE/SEM	CVs/CV%	Bias (95%CI)	LoA(95%CI)	
Individual STS duration (fast)	Regterschot et al.,2014(51)	ICC=0.87	SEM=6.7%	-	-	-	Moderate evidence
	Zhang et al.,2014(52)	ICC=0.86	SEM=7.5%	-	-	-	
	Cerrito et al.,2015(29)	ICC=0.92	SEM=6.9%	-	-	-	
	Greene et al.,2022(53)	Torso sensor:<br>ICC=0.74(0.67,0.80)	-	-	-	-	

		Thigh sensor: ICC=0.50(0.35,0.62)					
	Marques et al.,2021(30)	ICC=0.97(0.93,0.98)	SEM=0.05s	1.85%	-	-	
CV of single STS duration	Greene et al.,2022(53)	Torso sensor: ICC=0.30(0.10,0.47) Thigh sensor: ICC=0.57(0.45,0.67)	-	-	-	-	Very limited evidence
Individual STS duration (normal)	Regterschot et al.,2014(51)	ICC=0.66	SEM=8.3%	-	-	-	Limited evidence
	Zhang et al.,2014(52)	ICC=0.77	SEM=10.8%	-	-	-	
Total STS duration (fast)	Regterschot et al.,2014(51)	ICC=0.73	SEM=14.4%	-	-	-	Limited evidence
	Zhang et al.,2014(52)	ICC=0.90	SEM=9.0%	-	-	-	
	Greene et al.,2022(53)	Torso sensor: ICC=0.86(0.82,0.90) Thigh sensor: ICC=0.86(0.81,0.89)	-	-	-	-	
Stand-up time	Greene et al.,2022(53)	Torso sensor: ICC=0.83(0.78,0.87) Thigh sensor: ICC=0.36(0.17,0.51)	-	-	-	-	Limited evidence
	Marques et al.,2021(30)	ICC=0.92(0.82,0.96)	SEM=0.05s	3.03%	-	-	
CV of individual stand-up	Greene et al.,2022(53)	Torso sensor: ICC=0.50(0.35,0.61) Thigh sensor:	-	-	-	-	Very limited evidence

time		ICC=0 (0,0.21)					
Sit time	Greene et al.,2022(53)	Torso sensor: ICC=0.58(0.46,0.68) Thigh sensor: ICC=0.39(0.21,0.53)	-	-	-	-	Very limited evidence
CV of Sit time	Greene et al.,2022(53)	Torso sensor: ICC=0.30(0.10,0.47) Thigh sensor: ICC=0.33(0.14,0.49)	-	-	-	-	Very limited evidence
Maximal acceleration	Regterschot et al.,2014(51)	Normal speed ICC=0.83 Fast speed ICC=0.85	Normal speed SEM=1.2% Fast speed SEM=1.6%	-	-	-	Very limited evidence
Maximal jerk	Regterschot et al.,2014(51)	Normal speed: ICC=0.80 Fast speed: ICC=0.46	Normal speed: SEM=14.5% Fast speed: SEM=18.7%	-	-	-	Limited evidence
	Zhang et al.,2014(52)	Normal speed: ICC=0.78 Fast speed: ICC=0.66	Normal speed: SEM=20.7% Fast speed: SEM=21.2%	-	-	-	
Maximal velocity	Regterschot et al.,2014(51)	Normal speed ICC=0.91 Fast speed ICC=0.86	Normal speed SEM=7.5% Fast speed SEM=8.7%	-	-	-	Limited evidence
	Zhang et al.,2014(52)	Normal speed ICC=0.86 Fast speed	Normal speed SEM=13.0% Fast speed	-	-	-	

		ICC=0.82	SEM=11.5%				
Peak power	Regterschot et al.,2014(51)	Normal speed<br>ICC=0.96<br>Fast speed<br>ICC=0.91	Normal speed<br>SEM=6.9%<br>Fast speed<br>SEM=9.9%	-	-	-	Moderate evidence
	Cerrito et al.,2015(29)	ICC=0.91	SEM=26.1%	-	-	-	
	Zhang et al.,2014(52)	Normal speed<br>ICC=0.85<br>Fast speed<br>ICC=0.63	Normal speed<br>SEM=11.0%<br>Fast speed<br>SEM=13.8%	-	-	-	

ICC intraclass correlation coefficient; *LoA* limits of agreement; *r* pearson correlation coefficient; *MD* mean difference; *95%CI* 95% confidence interval; *AP* anterior-posterior; *ML* medio-lateral; *VT* vertical; Shaded represents that outcome have been included in the meta-analysis.

Supplementary Table9: Reliability assessment for timed up and go test outcomes.

	Study	Results					Evidence
		ICC/r (95%CI)	RMSE/SEM	Bias	Bias (95%CI)	LoA(95%CI)	
TUGT duration (fast speed)	Zhang et al.,2014(52)	ICC=0.90	SEM=5.7%	-	-	-	Limited evidence
	Smith et al.,2016(54)	Single task:<br>ICC=0.960(0.912,0.987)<br>Motor task:<br>ICC=0.916(0.821,0.971)<br>Cognitive task:<br>ICC=0.851(0.703,0.947)	Single task:<br>SEM=0.08(0.85%)<br>Motor task:<br>SEM =0.04(0.36%)<br>Cognitive task:<br>SEM =0.23(2.00%)	-	-	-	
TUGT duration (preferred	Regterschot et al.,2014(51)	ICC=0.98	SEM=8.5%	-	-	-	Limited evidence
	Zhang et al.,2014(52)	Normal speed	Normal speed	-	-	-	

speed)		ICC=0.80	SEM=12.4%				
Maximal jerk	Zhang et al.,2014(52)	Normal speed<br>ICC=0.85<br>Fast speed<br>ICC=0.86	Normal speed<br>SEM=15.2%<br>Fast speed<br>SEM=12.6%	-	-	-	Very limited evidence
Peak power	Zhang et al.,2014(52)	Normal speed<br>ICC=0.88<br>Fast speed<br>ICC=0.88	Normal speed<br>SEM=10.6%<br>Fast speed<br>SEM=8.5%	-	-	-	Very limited evidence
Steps count	Magistro et al.,2018(8)	Left:<br>ICC=0.927 (0.85,097)<br>Right:<br>ICC=0.914 (0.82, 0.96)	-	-	-	-	Limited evidence
	Smith et al.,2016(54)	Single task:<br>ICC=0.892(0.776,0.962)<br>Motor task:<br>ICC=0.865(0.727,0.952)<br>Cognitive task:<br>ICC=0.849(0.699,0.946)	Single task:<br>SEM=0.18(1.4%)<br>Motor task:<br>SEM<br>=0.16(1.18%)<br>Cognitive task:<br>SEM<br>=0.26(1.81%)	-	-	-	

ICC intraclass correlation coefficient; *LoA* limits of agreement; *r* pearson correlation coefficient; *MD* mean difference; *95%CI* 95% confidence interval; *AP* anterior-posterior; *ML* medio-lateral; *VT* vertical; Shaded represents that outcome have been included in the meta-analysis.

Supplementary Table10: Reliability assessment for 6 minute walk test outcomes.

	Study	Results					Evidence
		ICC/r (95%CI)	RMSE/SEM	CVs/CV%	Bias (95%CI)	LoA(95%CI)	
Walking speed	Donath et al.,2016(38)	0% slope<br>Slow speed:<br>ICC=0.994(0.985, 0.998)<br>Normal speed	-	-	-	-	Limited evidence

		ICC=0.995(0.987,0.998)<br>Fast speed<br>ICC=0.965(0.917,0.985)<br>15% slope<br>Slow speed:<br>ICC=0.996(0.991,0.998)<br>Normal speed<br>ICC=0.994(0.985,0.997)<br>Fast speed<br>ICC=0.993(0.982,0.997)					
	Werner et al.,2023(55)	preferred speed:<br>ICC=0.88(0.74,0.95)	preferred speed:<br>SEM(SEM%) =<br>0.07(m/s) (5.5%)	-	preferred speed:<br>-0.04(-0.08,0.01)<br>(m/s)	-	
Cadence	Donath et al.,2016(38)	0% slope<br>Slow speed:<br>ICC=0.929(0.826, 0.971)<br>Normal speed<br>ICC=0.948(0.881,0.978)<br>Fast speed<br>ICC=0.949(0.883,0.978)<br>15% slope<br>Slow speed:<br>ICC=0.871(0.702,0.944)<br>Normal speed<br>ICC=0.908(0.783,0.961)<br>Fast speed<br>ICC=0.936(0.846,0.973)	-	-	-	-	Very limited evidence
Stride time	Donath et al.,2016(38)	0% slope<br>Slow speed:<br>ICC=0.932(0.832,0.972)<br>Normal speed<br>ICC=0.948(0.880,0.977)<br>Fast speed<br>ICC=0.951(0.886,0.979)<br>15% slope<br>Slow speed:<br>ICC=0.867(0.693,0.942)<br>Normal speed<br>ICC=0.896(0.758,0.955)	-	-	-	-	Very limited evidence

		Fast speed ICC=0.925(0.826,0.967)					
Stride length	Donath et al.,2016(38)	0% slope<br>Slow speed: ICC=0.738(0.354,0.894)<br>Normal speed ICC=0.802(0.543,0.914)<br>Fast speed ICC=0.839(0.629,0.930)<br>15% slope<br>Slow speed: ICC=0.922(0.820,0.966)<br>Normal speed ICC=0.940(0.859,0.975)<br>Fast speed ICC=0.950(0.879,0.979)	-	-	-	-	Limited evidence
	Werner et al.,2023(55)	preferred speed: ICC=0.93(0.84,0.97)	preferred speed: SEM(SEM%) = 2.2(cm) (3.3%)	-	preferred speed: 0.3(-1.2,2.0) (cm)	-	
Steps count	Burton et al., 2018(40)	Fitbit Flex(blue): ICC=0.79(0.57,0.90)<br>Fitbit Flex(black): ICC=0.87(0.73,0.94)<br>Fitbit Charge HR (purple): ICC=0.96(0.91,0.98)<br>Fitbit Charge HR (balck): ICC=0.92(0.84,0.96)	-	-	-	-	Very limited evidence
Unbiased autocorrelation coefficients	Grimpampi et al., 2015(56)	AP: ICC=0.93<br>ML: ICC=0.95<br>VT: ICC=0.93	-	-	-	-	Very limited evidence

ICC intraclass correlation coefficient; *LoA* limits of agreement; *r* pearson correlation coefficient; *MD* mean difference; *95%CI* 95% confidence interval; *AP* anterior-posterior; *ML* medio-lateral; *VT* vertical; Shaded represents that outcome have been included in the meta-analysis.

Supplementary Table11: Definition of selected gait spatiotemporal outcomes.

Parameter	Description	Definition
Walking speed	Distance covered per second during gait	Total gait path/ total time

Cadence	steps per minute	Total steps/total minute
Stride time	Time elapsed between two ipsilateral consecutive heel contact	$HC_{left/right}(i+1)-HC_{left/right}(i)$
Step time	Time elapsed between a heel contact and contralateral heel contact	$HC_{left/right}(i+1)-HC_{right/left}(i)$
Stance time	Time elapsed between a heel contact and ipsilateral successive toes off	$TO_{left/right}(i) - HC_{left/right}(i)$
Swing time	Time elapsed between a toes off and ipsilateral successive heel contact	$HC_{left/right}(i) - TO_{left/right}(i)$

1. Álvarez MN, Ruiz ARJ, Neira GG, Huertas-Hoyas E, Cerda MTE, Delgado LP, et al. Assessing falls in the elderly population using G-STRIDE foot-mounted inertial sensor. Scientific reports. 2023;13(1):9208.
2. Byun S, Han JW, Kim TH, Kim KW. Test-Retest Reliability and Concurrent Validity of a Single Tri-Axial Accelerometer-Based Gait Analysis in Older Adults with Normal Cognition. PLoS ONE. 2016;11(7):e0158956.
3. Byun S, Lee HJ, Han JW, Kim JS, Choi E, Kim KW. Walking-speed estimation using a single inertial measurement unit for the older adults. PLoS ONE. 2019;14(12):e0227075.
4. Contreras C, Stanley EC, Deschamps-Prescott C, Burnap S, Hopkins M, Browning B, et al. Evaluation of Smartphone Technology on Spatiotemporal Gait in Older and Diseased Adult Populations. LID - 10.3390/s24175839 [doi] LID - 5839. (1424-8220 (Electronic)).
5. Ensink C, Smulders K, Warnar J, Keijsers N. Validation of an algorithm to assess regular and irregular gait using inertial sensors in healthy and stroke individuals. PeerJ. 2023;11:e16641.
6. Hartmann A, Luzi S, Murer K, de Bie RA, de Bruin ED. Concurrent validity of a trunk tri-axial accelerometer system for gait analysis in older adults. Gait & posture. 2009;29(3):444-8.
7. Maggio M, Ceda GP, Ticinesi A, De Vita F, Gelmini G, Costantino C, et al. Instrumental and Non-Instrumental Evaluation of 4-Meter Walking Speed in Older Individuals. PLoS ONE. 2016;11(4):e0153583.
8. Magistro D, Brustio PR, Ivaldi M, Esliger DW, Zecca M, Rainoldi A, et al. Validation of the ADAMO Care Watch for step counting in older adults. PLoS ONE. 2018;13(2):e0190753.
9. Kuntapun J, Silsupadol P, Kamnardsiri T, Lugade V. Smartphone Monitoring of Gait and Balance During Irregular Surface Walking and Obstacle Crossing. Frontiers in sports and active living. 2020;2:560577.
10. Rantalainen T, Pirkola H, Karavirta L, Rantanen T, Linnamo V. Reliability and concurrent validity of spatiotemporal stride characteristics measured with an ankle-worn sensor among older individuals. Gait & posture. 2019;74:33-9.

11. Rogan S, de Bie R, de Bruin ED. Sensor-based foot-mounted wearable system and pressure sensitive gait analysis Agreement in frail elderly people in long-term care. ZEITSCHRIFT FUR GERONTOLOGIE UND GERIATRIE. 2017;50(6):488-97.
12. Rudisch J, Joellenbeck T, Vogt L, Cordes T, Klotzbier TJ, Vogel O, et al. Agreement and consistency of five different clinical gait analysis systems in the assessment of spatiotemporal gait parameters. GAIT & POSTURE. 2021;85:55-64.
13. Ruediger S, Stuckenschneider T, Abeln V, Askew CD, Wollseiffen P, Schneider S, et al. Validation of a widely used heart rate monitor to track steps in older adults. JOURNAL OF SPORTS MEDICINE AND PHYSICAL FITNESS. 2019;59(10):1622-7.
14. Foster JI, Williams KL, Timmer BHB, Brauer SG. Concurrent Validity of the Garmin Vivofit (R) 4 to Accurately Record Step Count in Older Adults in Challenging Environments. JOURNAL OF AGING AND PHYSICAL ACTIVITY. 2022;30(5):833-41.
15. Maganja SA, Clarke DC, Lear SA, Mackey DC. Formative Evaluation of Consumer-Grade Activity Monitors Worn by Older Adults: Test-Retest Reliability and Criterion Validity of Step Counts. JMIR formative research. 2020;4(8):e16537.
16. Phillips LJ, Petroski GF, Markis NE. A comparison of accelerometer accuracy in older adults. Res Gerontol Nurs. 2015;8(5):213-9.
17. Matikainen-Tervola E, Cronin N, Aartolahti E, Sihvonen S, Sansgiri S, Finni T, et al. Validity of IMU sensors for assessing features of walking in laboratory and outdoor environments among older adults. (1879-2219 (Electronic)).
18. Digo E, Panero E, Agostini V, Gastaldi L. Comparison of IMU set-ups for the estimation of gait spatio-temporal parameters in an elderly population. Proceedings of the Institution of Mechanical Engineers Part H, Journal of engineering in medicine. 2023;237(1):61-73.
19. Micó-Amigo ME, Kingma I, Ainsworth E, Walgaard S, Niessen M, Van Lummel RC, et al. A novel accelerometry-based algorithm for the detection of step durations over short episodes of gait in healthy elderly. Journal of NeuroEngineering and Rehabilitation. 2016;13(1).
20. Bäcklund T, Öhberg F, Johansson G, Grip H, Sundström N. Novel, clinically applicable method to measure step-width during the swing phase of gait. Physiological measurement. 2020;41(6):065005.
21. Rantalainen T, Karavirta L, Pirkola H, Rantanen T, Linnamo V. Gait Variability Using Waist- and Ankle-Worn Inertial Measurement Units in Healthy Older Adults. Sensors (Basel, Switzerland). 2020;20(10).
22. Cole MH, Van Den Hoorn W, Kavanagh JK, Morrison S, Hodges PW, Smeathers JE, et al. Concurrent validity of accelerations measured using a tri-axial inertial measurement unit while walking on firm, compliant and uneven surfaces. PLoS ONE. 2014;9(5).
23. De Groote F, Vandevyvere S, Vanhevel F, Orban de Xivry J-J. Validation of a smartphone embedded inertial measurement unit for measuring postural stability in older adults. Gait & posture. 2021;84:17-23.
24. Pooranawatthanakul K, Siriphorn A. Testing the validity and reliability of a new android application-based accelerometer balance assessment tool for community-dwelling older adults. Gait & posture. 2023;104:103-8.
25. Ozinga SJ, Alberts JL. Quantification of postural stability in older adults using mobile technology. EXPERIMENTAL BRAIN RESEARCH. 2014;232(12):3861-72.

26. Ferrari LA-O, Bochicchio GA-O, Bottari AA-O, Scarton AA-O, Lucertini FA-O, Pogliaghi SA-O. Construct Validity of a Wearable Inertial Measurement Unit (IMU) in Measuring Postural Sway and the Effect of Visual Deprivation in Healthy Older Adults. LID - 10.3390/bios14110529 [doi] LID - 529. (2079-6374 (Electronic)).
27. Adamowicz L, Karahanoglu FI, Cicalo C, Zhang H, Demanuele C, Santamaria M, et al. Assessment of Sit-to-Stand Transfers during Daily Life Using an Accelerometer on the Lower Back. SENSORS. 2020;20(22).
28. Bochicchio G, Ferrari L, Bottari A, Lucertini F, Scarton A, Pogliaghi S. Temporal, Kinematic and Kinetic Variables Derived from a Wearable 3D Inertial Sensor to Estimate Muscle Power during the 5 Sit to Stand Test in Older Individuals: A Validation Study. Sensors. 2023;23(10).
29. Cerrito A, Bichsel L, Radlinger L, Schmid S. Reliability and validity of a smartphone-based application for the quantification of the sit-to-stand movement in healthy seniors. Gait & posture. 2015;41(2):409-13.
30. Marques DL, Neiva HP, Pires IM, Zdravevski E, Mihajlov M, Garcia NM, et al. An Experimental Study on the Validity and Reliability of a Smartphone Application to Acquire Temporal Variables during the Single Sit-to-Stand Test with Older Adults. SENSORS. 2021;21(6).
31. Song Y, Begum M, Arthanat S, LaRoche DP. Validation of Smartphone Accelerometry for the Evaluation of Sit-To-Stand Performance and Lower-Extremity Function in Older Adults. Journal of aging and physical activity. 2022;30(1):3-11.
32. Regterschot GRH, Zhang W, Baldus H, Stevens M, Zijlstra W. Accuracy and concurrent validity of a sensor-based analysis of sit-to-stand movements in older adults. Gait Posture. 2016;45:198-203.
33. Chan MHM, Keung DTF, Lui SYT, Cheung RTH. A validation study of a smartphone application for functional mobility assessment of the elderly. Hong kong physiotherapy journal. 2016;35:1-4.
34. Fudickar S, Hellmers S, Lau S, Diekmann R, Bauer JM, Hein A. Measurement System for Unsupervised Standardized Assessment of Timed "Up & Go" and Five Times Sit to Stand Test in the Community-A Validity Study. Sensors (Basel, Switzerland). 2020;20(10).
35. Orange ST, Metcalfe JW, Liefeith A, Jordan AR. Validity of various portable devices to measure sit-to-stand velocity and power in older adults. Gait & posture. 2020;76:409-14.
36. Hellmers S, Izadpanah B, Dasenbrock L, Diekmann R, Bauer JM, Hein A, et al. Towards an Automated Unsupervised Mobility Assessment for Older People Based on Inertial TUG Measurements. Sensors (Basel, Switzerland). 2018;18(10).
37. Walgaard S, Faber GS, van Lummel RC, van Dieen JH, Kingma I. The validity of assessing temporal events, sub-phases and trunk kinematics of the sit-to-walk movement in older adults using a single inertial sensor. JOURNAL OF BIOMECHANICS. 2016;49(9):1933-7.
38. Donath L, Faude O, Lichtenstein E, Pagenstert G, Nüesch C, Mündermann A. Mobile inertial sensor based gait analysis: Validity and reliability of spatiotemporal gait characteristics in healthy seniors. Gait Posture. 2016;49:371-4.
39. Kobsar D, Olson C, Paranjape R, Barden JM. The validity of gait variability and fractal dynamics obtained from a single, body-fixed triaxial accelerometer. Journal of applied biomechanics. 2014;30(2):343-7.

40. Burton E, Hill KD, Lautenschlager NT, Thøgersen-Ntoumani C, Lewin G, Boyle E, et al. Reliability and validity of two fitness tracker devices in the laboratory and home environment for older community-dwelling people. BMC geriatrics. 2018;18(1):103.
41. Bautmans I, Jansen B, Van Keymolen B, Mets T. Reliability and clinical correlates of 3D-accelerometry based gait analysis outcomes according to age and fall-risk. Gait & posture. 2011;33(3):366-72.
42. Hamacher D, Hamacher D, Taylor WR, Singh NB, Schega L. Towards clinical application: repetitive sensor position re-calibration for improved reliability of gait parameters. Gait & posture. 2014;39(4):1146-8.
43. Hartmann A, Murer K, de Bie RA, de Bruin ED. Reproducibility of spatio-temporal gait parameters under different conditions in older adults using a trunk tri-axial accelerometer system. Gait & posture. 2009;30(3):351-5.
44. Motti Ader LG, Greene BR, McManus K, Caulfield B. Reliability of inertial sensor based spatiotemporal gait parameters for short walking bouts in community dwelling older adults. GAIT & POSTURE. 2021;85:1-6.
45. Saunders NW, Koutakis P, Kloos AD, Kegelmeyer DA, Dicke JD, Devor ST. Reliability and validity of a wireless accelerometer for the assessment of postural sway. Journal of applied biomechanics. 2015;31(3):159-63.
46. Alqahtani BA, Sparto PJ, Whitney SL, Greenspan SL, Perera S, Brach JS. Psychometric properties of instrumented postural sway measures recorded in community settings in independent living older adults. BMC geriatrics. 2020;20(1):82.
47. Mancini M, Salarian A, Carlson-Kuhta P, Zampieri C, King L, Chiari L, et al. ISway: A sensitive, valid and reliable measure of postural control. Journal of NeuroEngineering and Rehabilitation. 2012;9(1).
48. Kosse NM, Caljouw S, Vervoort D, Vuillerme N, Lamoth CJ. Validity and Reliability of Gait and Postural Control Analysis Using the Tri-axial Accelerometer of the iPod Touch. Annals of biomedical engineering. 2015;43(8):1935-46.
49. Pedrero-Sánchez JF, De-Rosario-Martínez H, Medina-Ripoll E, Garrido-Jaén D, Serra-Añó P, Mollà-Casanova S, et al. The Reliability and Accuracy of a Fall Risk Assessment Procedure Using Mobile Smartphone Sensors Compared with a Physiological Profile Assessment. Sensors (Basel, Switzerland). 2023;23(14).
50. Peller A, Garib R, Garbe E, Komforti D, Joffe C, Magras A, et al. Validity and reliability of the NIH Toolbox R Standing Balance Test As compared to the Biodex Balance System SD. Physiotherapy theory and practice. 2023;39(4):827-33.
51. Regterschot GRH, Zhang W, Baldus H, Stevens M, Zijlstra W. Test-retest reliability of sensor-based sit-to-stand measures in young and older adults. Gait & posture. 2014;40(1):220-4.
52. Zhang W, Regterschot GRH, Schaabova H, Baldus H, Zijlstra W. Test-Retest Reliability of a Pendant-Worn Sensor Device in Measuring Chair Rise Performance in Older Persons. SENSORS. 2014;14(5):8705-17.
53. Greene BR, Doheny EP, McManus K, Caulfield B. Estimating balance, cognitive function, and falls risk using wearable sensors and the sit-to-stand test. Wearable Technol. 2022;3.
54. Smith E, Walsh L, Doyle J, Greene B, Blake C. The reliability of the quantitative timed up and go test (QTUG) measured over five consecutive days under

single and dual-task conditions in community dwelling older adults. Gait & posture. 2016;43:239-44.
55. Werner C, Hezel N, Dongus F, Spielmann J, Mayer J, Becker C, et al. Validity and reliability of the Apple Health app on iPhone for measuring gait parameters in children, adults, and seniors. Scientific reports. 2023;13(1):5350.
56. Grimpampi E, Oesen S, Halper B, Hofmann M, Wessner B, Mazza C. Reliability of gait variability assessment in older individuals during a six-minute walk test. JOURNAL OF BIOMECHANICS. 2015;48(15):4185-9.
